# A Standardized Temporal Segmentation Framework and Annotation Resource Library in Robotic Surgery

**DOI:** 10.1016/j.mcpdig.2025.100257

**Published:** 2025-08-22

**Authors:** Busisiwe Mlambo, Mallory Shields, Simon Bach, Armin Bauer, Andrew Hung, Omar Yusef Kudsi, Felix Neis, John Lazar, Daniel Oh, Robert Perez, Seth Rosen, Naeem Soomro, Michael Stany, Mark Tousignant, Christian Wagner, Ken Whaler, Lilia Purvis, Benjamin Mueller, Sadia Yousaf, Casey Troxler, Alfred Song, Emily Summers, Kiran Bhattacharyya, Anthony Jarc

**Affiliations:** a Advanced Research and Analytics, Intuitive Surgical, Sunnyvale, CA; b Medical Safety and Innovation, Intuitive Surgical, Sunnyvale, CA; c Department of Surgery, Queen Elizabeth Hospital, Birmingham, United Kingdom; d Research Institute for Women’s Health, Department of Women’s Health, University Hospital of Tübingen, Germany; e Cedars-Sinai Medical Center, Los Angeles, CA; f Department of Surgery, Good Samaritan Medical Center, Brockton, MA; g Brigham and Women’s Hospital, Boston, MA; h Division of Thoracic Surgery, Ascension Saint Thomas Hospital/University of Tennessee Health Science Center, Nashville, TN; i University of Southern California, Los Angeles, CA; j Virtua Memorial Hospital, Hainesport, NJ; k Department of Surgery, Emory University School of Medicine, Atlanta, GA; l Department of Urogynecology, Newcastle upon Tyne Hospitals Trust, Newcastle, United Kingdom; m Department of Gynecologic Surgery and Oncology, Ascension St. Thomas, Nashville, TN; n Prostate Center Gronau, San Antonius Hospital, Gronau, Germany

## Abstract

**Objective:**

To develop and share the first clinical temporal annotation guide library for 10 robotic procedures accompanied with a standardized ontology framework for surgical video annotation.

**Patients and Methods:**

A standardized temporal annotation framework of surgical videos paired with consistent, procedure-specific annotation guides is critical to enable comparisons of surgical insights and facilitate large-scale insights for exceptional surgical practice. Existing ontologies and guidance not only provide foundational frameworks but also provide limited scalability in clinical settings. Building on these, we developed a temporal annotation framework with nested surgical phases, steps, tasks, and subtasks. Procedure-specific annotation resource guides consistent with this framework that define each surgical segment with formulaic start and stop parameters and surgical objectives were iteratively created across 7 years (January 1, 2018, to January 1, 2025) through global research collaborations with surgeon researchers and industry scientists.

**Results:**

We provide the first resource library of annotation guides for 10 common robotic procedures consistent with our proposed temporal annotation framework, enabling consistent annotations for clinicians and large-scale data comparisons with computer-readable examples. These have been used in over 13,000 annotated surgical cases globally, demonstrating reproducibility and broad applicability.

**Conclusion:**

This resource library and accompanying ontology framework provide critical structure for standardized temporal segmentation in robotic surgery. This framework has been applied globally in private studies examining surgical objective performance metrics, surgical education, workflow characterization, outcome prediction, algorithms for surgical activity recognition, and more. Adoption of these resources will unify clinical, academic, and industry efforts, ultimately catalyzing transformational advancements in surgical practice.

Surgical data science (SDS), a rapidly growing field, aims to improve patient outcomes by harnessing technological advancements in surgery and computer science. A subfield within data science, SDS combines data science and objective surgical data to uncover actionable insights that underlie surgical performance, surgical workflow, patient outcomes, and more.^1^ For these technologies to deliver value, a standardized framework with accompanying clinical guides that define clinically relevant surgical segments across granularities, including phase, step, task, and subtask, is critical (Figure 1A). Such resources would enable clinicians to annotate and communicate their surgical case workflows consistently across institutions and catalyze scalable studies in SDS, such as the quantification and prediction of forces exerted on tissue within a clinically relevant surgical segment throughout new surgeons’ learning curves. Without this foundation, the field lacks the standardization and turn-key resources needed for major discoveries. As such, a standardized, clinically relevant, and computer-readable segmentation framework and annotation resource library are required to describe surgery across its temporal features.

**Figure 1:**
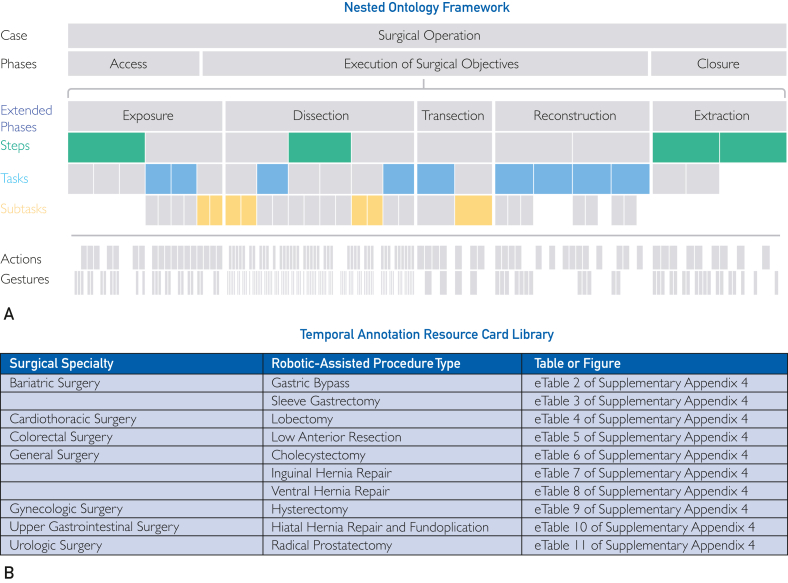
Nested temporal segmentation framework and procedures included in the temporal annotation resource card library. (A) The temporal annotation framework provided in this work is nested across extended phase (dark blue), step (green), task (light blue), and subtask (yellow) granularities and designed to be customizable to the needs of the use case. The pattern of the colored cells across the surgical case represents 1 such customized annotation approach. Also shown in gray are previous-phase recommendations, actions, and gestures, but these granularities are outside the scope of this work. (B) Surgical procedure types and their specialties that are provided in the procedure-specific annotation resource card library. The annotation resource card for each procedure type can be found in Supplemental Table 1 in Supplemental Appendix 4.

Early surgical temporal frameworks, ontologies, and foundational models exist, but they are insufficiently documented, focus on segments too small for procedural relevance or realistic annotation, are limited to 1 procedure type,^2^ or lack standardization. Pioneering studies in the “language of surgery” by the Hager group and others elegantly define and model surgical actions and gestures using surgical videos.^3^, ^4^, ^5^, ^6^, ^7^, ^8^ This work laid the foundation for detailed surgical movements, such as hand gestures with 1 or 2 joint motions, but they did not present a standardized framework with guidelines detailing the process of surgical segmentation for larger, procedurally relevant clinical segments, such as clinical steps, to achieve procedural objectives. Additionally, surgical process models (SPMs) have been developed for various use cases,^9^, ^10^, ^11^, ^12^ but they remain limited owing to a lack of available clinical data and inconsistent annotation processes across studies.^13^ A European initiative, the Ontology for representing Surgical Process Models Collaborative Action, seeks to develop a common ontology for SPMs through a network of research groups,^14^ but this is still in its infancy. Recent works to standardize surgical natural language and tools show exciting promise in adaptability and scalability, but these are vulnerable to the inherent reliability and consistency disadvantages of large language models.^15^^,^^16^ Although impactful, these and other efforts still require a clinically relevant standardized temporal segmentation framework that is applicable across procedure types.

In 2021, the Video Annotation Consensus project from the Society of Gastrointestinal and Endoscopic Surgeons (SAGES) artificial intelligence (AI) task force provided the first universal segmentation framework recommendations agnostic to surgical procedure, which recommended that clinically relevant segments be considered. This work was driven through Delphi consensus across experts in hospitals, surgeons, academia, and industry, and although it did not address specific clinical use cases, it laid the recommended foundation for the development of specific, validated ontologies with real surgical data across broad procedure types.^17^

We build on these efforts to describe a temporal segmentation framework for robotic-assisted surgery (RAS) with vertically nested extended phases, steps, tasks, and subtasks. Consistent with this framework, we provide the first library of 10 procedure type–specific surgical annotation resource guides with specific start and stop parameters for each segment (Figure 1B). Iterations of these annotation resource cards have been used to annotate thousands of surgical cases and compute context-aware objective performance indicators, enabling over 85 publications with insights into surgical experience and skill,^13^^,^^18^^,^^19^ training,^20^ workflows and efficiencies,^21^, ^22^, ^23^ complexities,^24^ patient factors and outcomes,^25^, ^26^, ^27^ and more.^28^ With these resources, the field can pursue clinical, academic, and industry collaborations with a common surgical language.

## Patients and Methods

### Development of a Nested and Customizable Ontology Framework

A nonredundant temporal framework was developed for surgical segments to be nested hierarchically within granularity levels, consisting of extended phases, steps, tasks, and subtasks (Figure 1A). Adapted from the temporal annotation framework proposed by the SAGES AI task force,^17^ all steps are nested within a surgical phase, each task is nested within a surgical step, and each subtask is nested within a task (Figure 1A). This nesting aligns all segmental boundaries such that a required start or stop parameter for any lower granularity segment is consistent with a start and/or stop parameter of a higher granularity segment through the completion of a specific surgical intent and action. Although gestures are critical building blocks of surgical procedures (Figure 1A), the scope of this work excluded them because of their specificity, significant effort to annotate, and limited procedural context individually (Figure 1A).

With this framework, annotations can be tailored to the granularity of specific project needs, research questions, and AI developments. This “sliding scale” balances granularity with annotation effort while maintaining consistent start and stop parameters and clinical intents for data set combination and consistency. In this way, clinical researchers interested in subtasks nested beneath the “Mobilization of the Splenic Flexure” task throughout a colorectal case can annotate 1 or more specific subtasks for their research, but they have the flexibility to annotate the remainder of the case at broader segment levels, such as step or extended phase. This provides a fully annotated case with the detail needed for the study without the effort to annotate the full case at high granularity. To visualize this, colored cells in Figure 1A provide an example of specific segments that can be annotated across granularities within a single case. Full ontology examples can be found in Supplemental Appendices 1 and 2 (available online at https://www.mcpdigitalhealth.org/ and as computer-readable .json files upon request).

### Procedure-Specific Temporal Annotation Resource Card Development

Within this nested framework, recommended segments for annotation for 10 commonly used robotic surgical procedures across bariatric, cardiothoracic, colorectal, general, gynecologic, upper gastrointestinal, and urologic specialties were established (Figure 1B). These guides have been iteratively developed for over 7 years (January 1, 2018, to January 1, 2025) in partnership with domain experts across industry, clinical surgery, and 12+ international research institutions.

Before the development of each procedure-specific annotation card, common variations of the procedure of interest are reviewed by surgeons and a professional annotation team to anticipate variabilities across populations of surgeons and techniques. Each annotation resource card provides the name, granularity level, surgical objective, and start/stop parameters for each recommended procedure type segment. To create each card, a standardized, 3-component equation is used. First is the identification of the overall surgical objective underlying each segment (extended phase, step, task, or subtask). Second, each segment is functionally named with the inclusion of a surgical intention through at least 1 surgical action, represented as a verb, such that each name describes what is to be completed during that segment. For example, “Gallbladder off Liver Bed” is not an acceptable segment name, but “Dissection of Gallbladder off Liver Bed” is. Third, ie, start and stop parameters are defined by a specific interaction between a surgical tool type and its target (patient anatomy or surgical material), which initiates or completes that surgical action(s) and intention. This equation considers the Ontology for representing Surgical Process Models ontology concept underlying tool type-target interactions,^14^ and the anatomic tissues identified consider the foundational models of anatomy ontology.^29^ Using this equation, the start parameter of the Dissection of Gallbladder off Liver Bed step in a cholecystectomy procedure is as follows: “First dissecting tool interaction with gallbladder with the intent to dissect the gallbladder off the liver bed.”

Once drafted, collaborating surgeon expert(s) review the resource card with industry scientists trained in surgical annotation and provide additional clinical context and recommendations for its next iteration. Surgical case videos are used to assess the card for completeness and clinical relevance. If needed, card revisions to clarify start/stop parameter definitions and capture all variations across surgical approaches are made.

### Interrater Variability Assessment

To assess interrater variability and ensure robust annotation reproducibility, an internal quality assessment is performed for each procedure types 1 to 4 times each year (detailed methodology and results found in Supplemental Appendix 3, available online at https://www.mcpdigitalhealth.org/).

## Results

### Definitions and Components of Extended Surgical Phases, Steps, Tasks, and Subtasks

#### Extended Surgical Phases

**Figure 2:**
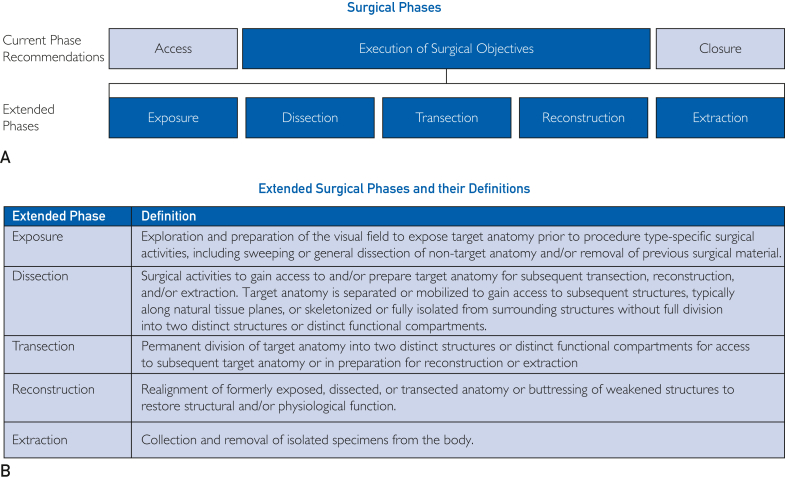
Extended surgical phases and their definitions. (A) Schematic of current-phase recommendations (above) and the proposed extended phases within the “Execution of Surgical Objectives” phase (blue, below). The extended phases, exposure, dissection, transection, reconstruction, and extraction provide standardized segmentations with additional clinical context agnostic to procedure type. (B) Definitions for each extended phase.

**Figure 3:**
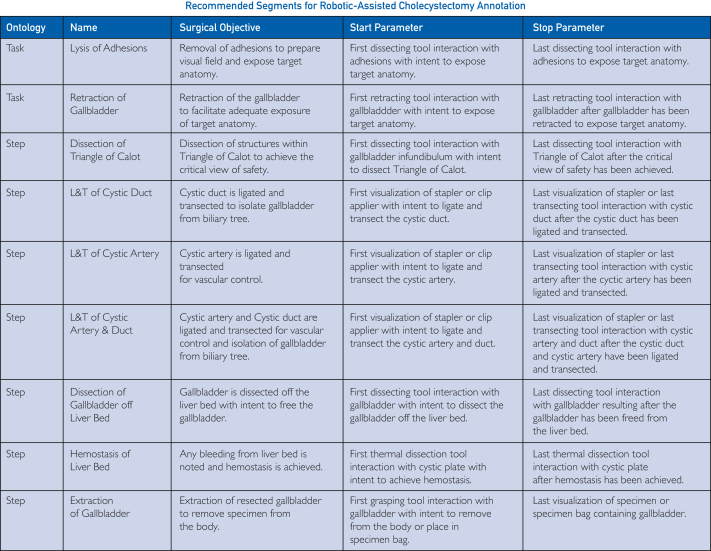
Temporal annotation resource card recommended segments specific to robotic-assisted cholecystectomy. For each recommended annotation segment, the table includes the ontological granularity level, the segment name, its surgical objective, and the start and stop parameters for each. L&T, ligation and transection.

**Figure 4:**
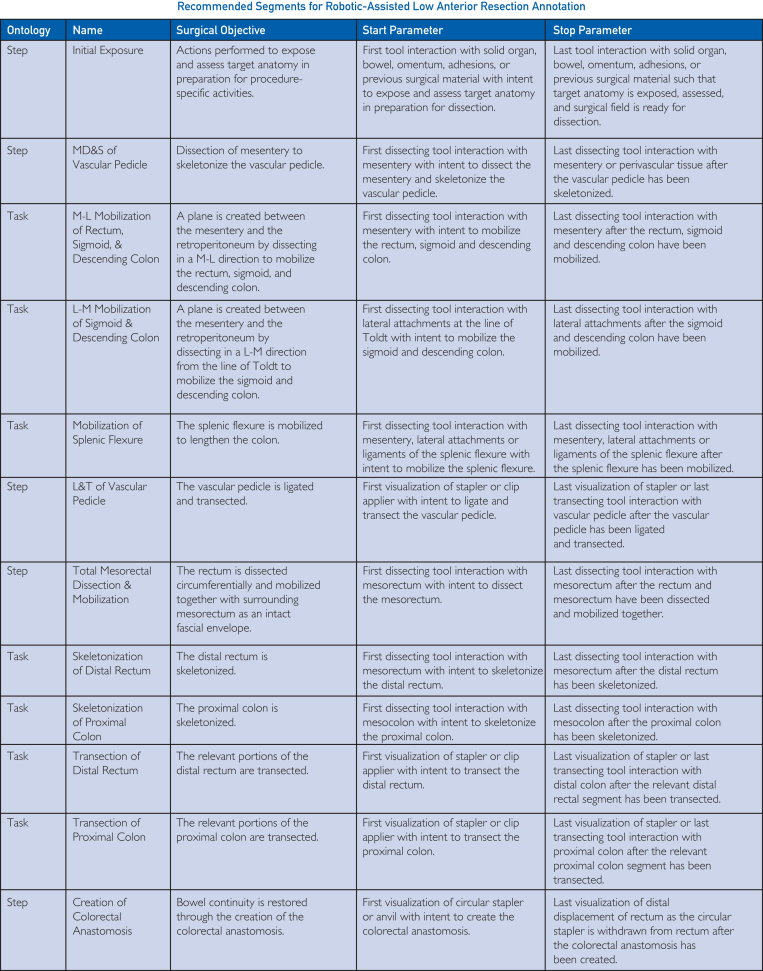
Temporal annotation resource card recommended segments specific to robotic-assisted low anterior resection. For each recommended annotation segment, the table includes the ontological granularity level, the segment name, its surgical objective, and the start and stop parameters for each. L-M, lateral to medial; L&T, ligation and transection; MD&S, mesenteric dissection and skeletonization; M-L, medial to lateral.

By recommendation of the SAGES AI Task Force, all surgeries consist of 3 procedurally agnostic phases: access, execution of surgical objectives, and closure.^17^ To cater to RAS and endoscopic video, the previously established access and closure phases were not used here. Instead, the following extended phases that encompass the previously proposed “Execution of Surgical Objectives” phase were established: exposure, dissection, transection, reconstruction, and extraction (Figures 1A and 2). Extended surgical phases are the broadest, least granular tier of temporal segmentation within the endoscopic video but provide more clinical context than Execution of Surgical Objectives alone. Each extended phase is defined in Figure 2B. Not all extended phases are required for every procedure, but if present, each will have at least 1 defined surgical step nested beneath it (Figure 1A). Extended phases may appear multiple times across the operation and in different orders, such that differences in workflows can be identified. Since each phase has at least 1 step nested beneath it and is applicable across surgical specialties and procedure types, start and stop parameters for extended phases are unique in that they are reliant on the start and stop parameters of the segments nested beneath them (Figures 3 and 4; Supplementary Appendices 1 and 2, and Supplemental Tables 2-11 in Supplemental Appendix 4, available online at https://www.mcpdigitalhealth.org/). Together, these extended phases constitute the core, procedurally agnostic and clinically relevant temporal segments of surgery throughout endoscopic video capture.

#### Surgical Steps

**Figure 5:**
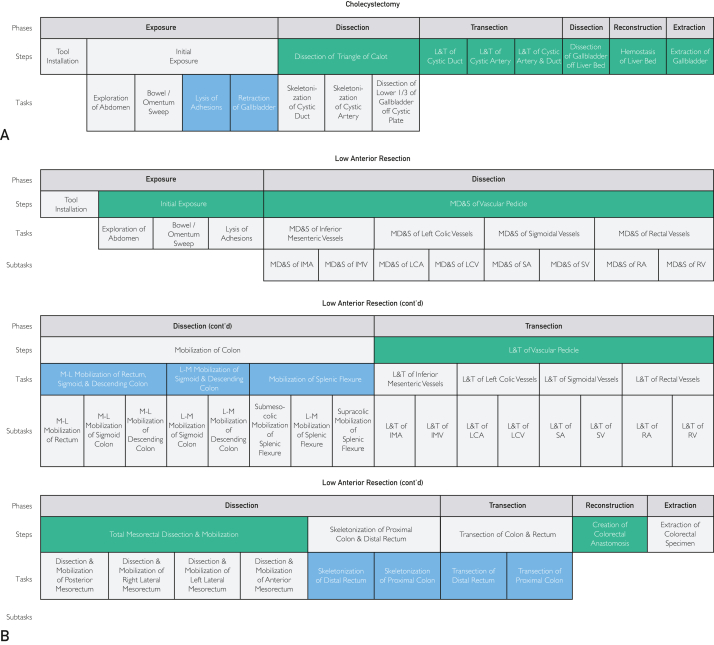
Nested temporal annotation frameworks for 2 common robotic procedures—cholecystectomy and low anterior resection (LAR). (A) Nested annotation framework for cholecystectomy. Each segment within cholecystectomy is delineated across extended phases (top row, dark blue), steps (middle row, green), and tasks (bottom row, blue). (B) Nested annotation framework for LAR. Like cholecystectomy, extended phases (top row, dark blue), steps (second row, green), and tasks (third row, blue) are represented. LAR also includes subtasks (bottom row, yellow). IMA, inferior mesenteric artery; IMV, inferior mesenteric vein; LCA, left colic artery; LCV, left colic vein; L-M, lateral to medial; L&T, ligation and transection; MD&S, mesenteric dissection and skeletonization; M-L, medial to lateral; RA, rectal artery; RV, rectal vein; SA, sigmoidal artery; SV, sigmoidal vein. Color-shaded cells depict currently recommended temporal segments for annotation, and light gray cells represent phase, step, task, or subtask delineations that have been established and nested for flexibility across use cases at scale.

Steps are the second hierarchical tier of temporal segmentation, with at least 1 step always present beneath a surgical phase if that surgical phase is present. Steps define specific surgical objectives with delineated start and stop parameters, which can be specialty or procedure-specific. Examples of surgical steps are the “Dissection of Triangle of Calot” within the “Dissection” phase of cholecystectomy cases and the “Mobilization of the Colon” step, also within the “Dissection” phase in colorectal cases (Figure 5). This definition does not require steps to be procedure-specific. As such, note that the Dissection of Triangle of Calot step is procedure-specific to cholecystectomy, but the “Mobilization of Colon” step can be used for several procedures within the colorectal specialty, such as low anterior resection (LAR) and right colectomy.

One or multiple steps may be needed to complete each surgical phase, and similar to phases, steps may be present multiple times and in different orders throughout an operation. Steps may be used to complete a surgical phase together, such as the Dissection of Triangle of Calot step and the Dissection of Gallbladder off Liver Bed step to encompass the Dissection phase of cholecystectomy (Figure 5A), but steps may also provide additional clinical context on surgical approach or workflow. For example, annotation of the single “Ligation and Transection of Cystic Artery and Duct” step would signify a different surgical approach than annotation of the “Ligation and Transection of Cystic Duct” step followed by the “Ligation and Transection of Cystic Artery” step separately. In the former case, the annotation indicates the duct and artery were dissected together as the surgical intent, whereas, in the latter example, the duct and artery were chronologically dissected distinctly from one another (Figure 5A).

#### Surgical Tasks and Subtasks

Tasks and subtasks are more granular hierarchical tiers of temporal segmentation. Tasks provide targeted objectives necessary to accomplish a surgical step. For example, tasks within the Dissection of Triangle of Calot step are the “Skeletonization of Cystic Duct,” “Skeletonization of Cystic Artery,” and the “Dissection of Lower One-Third of Gallbladder off Cystic Plate” (Figure 5A), which individually complete a targeted objective but together encompass the Dissection of Triangle of Calot step. Similarly, the “Mobilization of Splenic Flexure” task is an essential component of the Mobilization of Colon step in colorectal cases requiring mobilization of the splenic flexure for oncologic resection or when elongation of the colon is necessary to achieve a tension-free and well-vascularized anastomosis (Figure 5B).

Much like steps, tasks can be used to provide additional clinical context. For example, within the Mobilization of Colon step, the “Medial to Lateral Mobilization of Rectum, Sigmoid, and Descending Colon” and “Lateral to Medial Mobilization of Sigmoid and Descending Colon” tasks highlight different approaches to mobilize the colon and rectum (Figure 5B). Although tasks are critical components within this framework, they are not always required beneath each step. For example, there is no task nested beneath the Dissection of Gallbladder off Liver Bed step (Figure 5A) in cholecystectomy because no specific critical or clinically relevant combinations of surgical objectives with defined start/stop parameters are required to complete that step. However, tasks could be added beneath this step as frameworks evolve, such as the establishment of new techniques to complete that step.

Subtasks, the most granular tier of temporal annotation incorporated within our current framework, define detailed surgical activities encompassed within a surgical task. As such, additional clinical context into the approaches taken to accomplish surgical tasks, including considerations such as whether “Submesocolic Mobilization of Splenic Flexure,” “Supramesocolic Mobilization of Splenic Flexure,” or “Lateral to Medial Mobilization of Splenic Flexure” subtasks were used by the surgeon as different methods to execute the “Mobilization of Splenic Flexure” task can be captured (Figure 5B). Although subtasks provide an increased level of segmentation granularity, many procedures do not necessitate them, such as in the cholecystectomy example (Figure 5A). In fact, utilization of subtask annotations should be limited because the annotation effort required for such granular segmentation is increased owing to necessitated attention to finer details and nuances, which decreases annotation efficiency and increases interrater variability.

### Procedure-Specific Temporal Annotation Resource Card Library

Procedure-specific temporal annotation resource guide cards were established across bariatric, cardiothoracic, colorectal, general, gynecologic, upper gastrointestinal, and urologic surgical specialties. Each resource card defines the start and stop parameters for all identified and defined surgical segments within that procedure. However, it is not recommended that all surgical segments across granularities are annotated. As such, we provide segment annotation recommendations that balance clinical granularity and annotation effort. The recommended annotation segments for cholecystectomy and LAR can be found in Figures 3 and 4, respectively, with full resource cards for 10 procedure types with recommended segments highlighted available in Supplemental Appendix 4 (available online at https://www.mcpdigitalhealth.org/). Visual representations of all nested procedural segments with annotation recommendations are found in Figure 5A,B for cholecystectomy and LAR and in Supplemental Figures 2-9 (available online at https://www.mcpdigitalhealth.org/) for the remaining procedure types.

### Utilization and Validation

Between January 1, 2018, and January 1, 2025, these resource guides have been used across 13,465 cases with over 15,000 hours of surgical video assessed, supporting applications and insights into RAS workflow analysis, skill assessment, surgical outcomes predictions, and algorithm development.^28^ Interrater variability assessments for each procedure type are regularly completed to ensure annotation consistency and described in Supplemental Appendix 3.

## Discussion

This work outlines a universally applicable temporal segmentation framework for surgical annotation, which generates standardization for clinical communications and research and SDS. In addition, it provides the first temporal annotation resource guide library of 10 common robotic procedure types (Supplemental Appendix 4). With considerations for clinically relevant scenarios and scalability for both clinical and big data applications, this work provides pragmatic resources for a common language in surgery.

Although these resources are significant, more work will be needed in universal annotation frameworks in RAS. As such, our flexible annotation framework has been designed for future iteration and evolution. Much work has been done to identify and leverage surgical gestures,^3^, ^4^, ^5^, ^6^, ^7^, ^8^ and we anticipate the annotation of surgical gestures to one day be nested under subtasks and ascribed with start/stop parameters related to specific tool type-target interactions and actions. With the recent advantages in generative AI based on language models, foundational models are being pursued and show early promise.^15^^,^^16^

Beyond the temporal annotation framework presented in this study, a spatial annotation framework of anatomical features and critical surgical events are beyond the scope of this article. Much like temporal segmentation, guidelines for spatial and anatomical segmentations have been proposed, and data-driven efforts in this space have made much progress.^17^ However, a common spatial annotation language established with large volumes of clinical cases is still needed.^30^ Frameworks for annotation and automatic identification of critical events, such as specific organs,^31^ tissue damage and bleeding,^32^ critical views for surgical safety,^33^^,^^34^ and more, are areas of active research in the computer vision and AI space.^35^ With these breakthroughs, significant future work will be needed to join both spatial and temporal frameworks together as a comprehensive model of surgical components. Together, this work provides critical context for advances in intraoperative clinical decision support and education.

### Limitations

Although this work provides critical annotation resources to the surgical community, inherent limitations and challenges remain. We provide the first 10 temporal annotation resource cards for utilization in the field, but this is not a comprehensive library of robotic surgical procedures. Our group continues to expand on the establishment of new resource cards across procedure types, but there is much work to be done. Additionally, a “Goldie locks” problem exists where a perfect balance between annotation effort and clinical granularity is elusive, especially when considering the variable needs of researchers, data scientists, and clinicians in the SDS community. The flexibility of our proposed framework mitigates this to an extent and enables individual working groups to apply a sliding scale for “best fit” to their work, but it still may not provide the necessary detail for all use cases and is less adaptable than future foundational models that may address this limitation. Additionally, surgery is constantly evolving with advances in surgical approaches, techniques, instrumentation, and more. Therefore, the annotation frameworks set forth in this study will need to continue to iterate and adapt.

## Conclusion

We provide a temporal segmentation framework for RAS to enable clinical, academic, SDS, and industry teams to drive surgical insights and technological innovations collaboratively at scale. These resources will lead to transformational changes in surgical care that will ultimately improve patient outcomes.

## Potential Competing Interests

During this work and at the time of writing, Dr Mlambo, Dr Shields, Dr Oh, Dr Tousignant, Mr Whaler, Ms Purvis, Mr Mueller, Dr Yousaf, Ms Troxler, Dr Song, Ms Summers, Dr Bhattacharyya, and Dr Jarc were employees of Intuitive Surgical. Drs Bach, Rosen, and Kudsi have received research grant support from Intuitive Surgical for unrelated research efforts. Dr Hung has received consulting fees from Intuitive Surgical and Teleflex. Dr Kudsi has received grants from Gore and consulting fees from Gore, Bard, and Intuitive. Dr Lazar is a paid consultant for Intuitive Surgical, is on the Advisory Board of OTL, and has received honoraria as a lecturer for Medtronic and AstraZeneca. Dr Perez is an educational presenter and instructor for robotic-assisted surgery paid for by Intuitive Surgical. Dr Wagner has received contracts from Intuitive, Grena, BD Bard, Synektik, and Medicaroid; has received consultant fees from Intuitive Surgical and DGRU; has received payment for expert testimony from Intuitive Surgical; has participated on a safety monitoring board or advisory board at Intuitive Surgical and Medicaroid, and has a board role at DRGU and ERUS.

## Ethics Statement

The data utilized in this study are retrospective and were never paired to patient data for this effort (only protected health information-scrubbed videos were available to the research team for development of the segmentation framework and annotation resource guides), and fall within the bounds of WCG approved IRB study #1340365.
