# Supplementary material for: A Standardized Temporal Segmentation Framework and Annotation Resource Library in Robotic Surgery
Source: Mayo Clin Proc Digit Health. 2025 Aug 22;3(4):100257. doi: 10.1016/j.mcpdig.2025.100257 (PMC12492233; doi:10.1016/j.mcpdig.2025.100257)
Supplement: Supplementary Appendix 1 [file mmc1.pdf]

```
{
    "ontology": {
        "name": "SurgicalTemporalAnnotationOntology_cholecystectomy",
        "description": "A nested and customizable ontology for
annotating surgical activities in surgical videos.",
        "granularityLevels": [
            {
                "name": "Extended phase",
                "description": "The least granular tier of temporal
segmentation in the surgical procedure."
            },
            {
                "name": "Step",
                "description": "The second hierarchical tier of
temporal segmentation, which define specific surgical intentions
through completion of surgical actions."
            },
            {
                "name": "Task",
                "description": "The third hierarchical tier of
temporal segmentation, which define specific surgical intentions
through completion of surgical actions."
            }
        ],
        "Actions": [
            {
                "name": "Dissection",
                "description": "Using instruments to separate
anatomical structures."
            },
            {
                "name": "Transection",
                "description": "Using instruments to divide an
anatomical structure into two distinct structures."
            },
            {
                "name": "Ligation",
                "description": "Using instruments to tie off or
occlude anatomical structures, such as blood vessels."
            },
            {
                "name": "Retraction",
                "description": "Manipulation of tissue to improve
exposure."
            },
            {
                "name": "Mobilization",
                "description": "Using instruments to move or free
anatomical structures from surrounding tissue."
            },
```

```
            {
                "name": "Skeletonization",
                "description": "Using instruments to remove
surrounding tissue and expose underlying target anatomical
structures."
            },
            {
                "name": "Sweeping",
                "description": "Using instruments to gently move or
push anatomical structures, such as bowel or omentum, out of the way."
            },
            {
                "name": "Extraction",
                "description": "Using instruments to remove anatomical
structures, specimens, or other material from the body."
            },
            {
                "name": "Anastomosis",
                "description": "Using instruments to connect or join
anatomical structures, such as blood vessels or bowel segments."
            },
            {
                "name": "Hemostasis",
                "description": "Control of bleeding during surgery."
            },
            {
                "name": "Exploration",
                "description": "Using instruments to examine or
inspect anatomical structures."
            },
            {
                "name": "Installation",
                "description": "Insertion of instruments into the body
cavity."
            }
        ],
        "procedures": [
            {
                "name": "Cholecystectomy",
                "description": "Surgical removal of the gallbladder.",
                "Extended phases": [
                    {
                        "name": "Exposure",
                        "description": "Exploration and preparation of
the visual field with intent to expose target anatomy prior to
procedure type-specific surgical activities, including sweeping or
general dissection of non-target anatomy and/or removal of previous
surgical material.",
                        "start-parameter": {
                            "description": "Mirrors start parameter of
```

```
first nested chronological segment annotated beneath Exposure phase.",
                            "intent-to": [
                                "tool",
                                "action",
                                "anatomy"
                            ]
                        },
                        "stop-parameter": {
                            "description": "Mirrors stop parameter of
last nested chronological segment annotated beneath Exposure phase.",
                            "completion-of": [
                                "tool",
                                "action",
                                "anatomy"
                            ]
                        },
                        "steps": [
                            {
                                "name": "Tool Installation",
                                "description": "Installation of
robotic instruments through ports into the body cavity to prepare for
surgical activity.",
                                "start-parameter": {
                                    "description": "First
visualization of first tool as it enters the body cavity.",
                                    "intent-to": {
                                        "tool": [
                                            "tool"
                                        ],
                                        "action": [
                                            "visualize"
                                        ],
                                        "anatomy": [
                                            "body cavity"
                                        ]
                                    }
                                },
                                "stop-parameter": {
                                    "description": "Last tool movement
immediately after last tool is installed into body cavity.",
                                    "completion-of": {
                                        "tool": [
                                            "tool"
                                        ],
                                        "action": [
                                            "install"
                                        ],
                                        "anatomy": [
                                            "body cavity"
                                        ]
```

```
                                        }
                                    },
                              "tasks": []
                                {
                                    "name": "Initial Exposure",
                                    "description": "Actions performed to
expose and assess target anatomy in preparation for procedure-specific
activities.",
                                    "start-parameter": {
                                        "description": "First tool
interaction with solid organ, bowel, omentum, adhesions, or previous
surgical material with intent to expose and assess target anatomy in
preparation for dissection.",
                                        "intent-to": {
                                            "tool": [
                                                "tool"
                                            ],
                                            "action": [
                                                "expose",
                                                "assess"
                                            ],
                                            "anatomy": [
                                                "solid organ",
                                                "bowel",
                                                "omentum",
                                                "adhesions",
                                                "previous surgical
material"
                                            ]
                                        }
                                    },
                                    "stop-parameter": {
                                        "description": "Last tool
interaction with solid organ, bowel, omentum, adhesions, or previous
surgical material such that target anatomy is exposed, assessed, and
surgical field is ready for dissection.",
                                        "completion-of": {
                                            "tool": [
                                                "tool"
                                            ],
                                            "action": [
                                                "expose",
                                                "assess"
                                            ],
                                            "anatomy": [
                                                "solid organ",
                                                "bowel",
                                                "omentum",
                                                "adhesions",
                                                "previous surgical
```

```
material"
                                        ]
                                    }
                                },
                                "tasks": [
                                    {
                                        "name": "Exploration of
Abdomen",
                                        "description": "Exploration of
abdomen to assess pathology.",
                                        "start-parameter": {
                                            "description": "First
endoscope focus on abdominal anatomy with intent to explore anatomy
and assess pathology.",
                                            "intent-to": {
                                                "tool": [
                                                    "endoscope"
                                                ],
                                                "action": [
                                                    "explore",
                                                    "assess"
                                                ],
                                                "anatomy": [
                                                    "abdominal
anatomy"
                                                ]
                                            }
                                        },
                                        "stop-parameter": {
                                            "description": "End of
exploratory endoscope movements across abdomen after pathology is
assessed.",
                                            "completion-of": {
                                                "tool": [
                                                    "endoscope"
                                                ],
                                                "action": [
                                                    "move"
                                                ],
                                                "anatomy": [
                                                    "abdomen"
                                                ]
                                            }
                                        }
                                    },
                                    {
                                        "name": "Bowel / Omentum
Sweep",
                                        "description": "Sweeping of
bowel or omentum with intent to expose target anatomy.",
```

```
                                        "start-parameter": {
                                            "description": "First tool
interaction with bowel or omentum with intent to expose target
anatomy.",
                                            "intent-to": {
                                                "tool": [
                                                    "tool"
                                                ],
                                                "action": [
                                                    "expose"
                                                ],
                                                "anatomy": [
                                                    "bowel"
                                                    "omentum"
                                                ]
                                            }
                                        },
                                        "stop-parameter": {
                                            "description": "Last tool
interaction with bowel or omentum to expose target anatomy.",
                                            "completion-of": {
                                                "tool": [
                                                    "tool"
                                                ],
                                                "action": [
                                                    "expose"
                                                ],
                                                "anatomy": [
                                                    "bowel",
                                                    "omentum"
                                                ]
                                            }
                                        }
                                    },
                                    {
                                        "name": "Lysis of Adhesions",
                                        "description": "Removal of
adhesions to prepare visual field and expose target anatomy.",
                                        "start-parameter": {
                                            "description": "First
dissecting tool interaction with adhesions with intent to expose
target anatomy.",
                                            "intent-to": {
                                                "tool": [
                                                    "dissecting tool"
                                                ],
                                                "action": [
                                                    "dissect",
                                                ],
                                                "anatomy": [
```

```
                                                        "adhesions"
                                                    ]
                                                }
                                            },
                                            "stop-parameter": {
                                                "description": "Last
dissecting tool interaction with adhesions to expose target anatomy.",
                                                "completion-of": {
                                                    "tool": [
                                                        "dissecting tool"
                                                    ],
                                                    "action": [
                                                        "expose",

"dissect"
                                                    ],
                                                    "anatomy": [
                                                        "target anatomy",
                                                        "adhesions"
                                                    ]
                                                }
                                            }
                                        }
                                    ]
                                },
                                {
                                    "name": "Retraction of Gallbladder",
                                    "description": "Retraction of the
gallbladder to facilitate adequate exposure of target anatomy.",
                                    "start-parameter": {
                                        "description": "First retracting
tool interaction with gallbladder with intent to expose target
anatomy.",
                                        "intent-to": {
                                            "tool": [
                                                "retracting tool"
                                            ],
                                            "action": [
                                                "retract",
                                            ],
                                            "anatomy": [
                                                "gallbladder",
                                            ]
                                        }
                                    },
                                    "stop-parameter": {
                                        "description": "Last retracting
tool interaction with gallbladder after gallbladder has been retracted
to expose target anatomy.",
                                        "completion-of": {
```

```
                                "tool": [
                                    "retracting tool"
                                ],
                                "action": [
                                    "retract",
                                    "expose"
                                ],
                                "anatomy": [
                                    "gallbladder",
                                    "target anatomy"
                                ]
                            }
                        },
                    }
                ]
            },
            {
                "name": "Dissection",
                "description": "Surgical activities to gain
access to and/or prepare target anatomy for subsequent transection,
reconstruction, and/or extraction. Target anatomy is separated or
mobilized to gain access to subsequent structures, typically along
natural tissue planes, or skeletonized or fully isolated from
surrounding structures without full division into two distinct
structures or distinct functional compartments.",
                "start-parameter": {
                    "description": "Mirrors start parameter of
first nested chronological segment annotated beneath Dissection
phase.",
                    "intent-to": [
                        "tool",
                        "action",
                        "anatomy"
                    ]
                },
                "stop-parameter": {
                    "description": "Mirrors stop parameter of
last nested chronological segment annotated beneath Dissection
phase.",
                    "completion-of": [
                        "tool",
                        "action",
                        "anatomy"
                    ]
                },
                "steps": [
                    {
                        "name": "Dissection of Triangle of
Calot",
                        "description": "Dissection of
```

```
structures within Triangle of Calot to visualize the critical view of
safety.",
                                "start-parameter": {
                                    "description": "First dissecting
tool interaction with gallbladder infundibulum with intent to dissect
Triangle of Calot.",
                                    "intent-to": {
                                        "tool": [
                                            "dissecting tool"
                                        ],
                                        "action": [
                                            "dissect"
                                        ],
                                        "anatomy": [
                                            "gallbladder
infundibulum",

                                            "Triangle of Calot"
                                        ]
                                    }
                                },
                                "stop-parameter": {
                                    "description": "Last dissecting
tool interaction with Triangle of Calot after the Triangle of Calot
has been dissected and the critical view of safety has been
achieved.",
                                    "completion-of": {
                                        "tool": [
                                            "dissecting tool"
                                        ],
                                        "action": [
                                            "dissect"
                                        ],
                                        "anatomy": [
                                            "Triangle of Calot"
                                        ]
                                    }
                                },
                                "tasks": [
                                    {
                                        "name": "Skeletonization of
Cystic Duct",
                                        "description":
"Skeletonization of cystic duct from surrounding fibro-fatty
tissues.",
                                        "start-parameter": {
                                            "description": "First
dissecting tool interaction with fibro-fatty tissue in Triangle of
Calot with intent to skeletonize the cystic duct.",
                                            "intent-to": {
                                                "tool": [
```

"dissecting tool"
],
"action": [
"skeletonize"
],
"anatomy": [
"fibro-fatty
tissue in Triangle of Calot",
"cystic duct"
]
}
},
"stop-parameter": {
"description": "Last
dissecting tool interaction with fibro-fatty tissue in Triangle of
Calot after the cystic duct has been skeletonized.",
"completion-of": {
"tool": [
"dissecting tool"
],
"action": [
"skeletonize"
],
"anatomy": [
"fibro-fatty
tissue in Triangle of Calot",
]
}
}
},
{
"name": "Skeletonization of
Cystic Artery",
"description":
"Skeletonization of cystic artery from surrounding fibro-fatty
tissues.",
"start-parameter": {
"description": "First
dissecting tool interaction with fibro-fatty tissue in Triangle of
Calot with intent to skeletonize the cystic artery.",
"intent-to": {
"tool": [
"dissecting tool"
],
"action": [
"skeletonize"
],
"anatomy": [
"fibro-fatty
tissue in Triangle of Calot",

```
"cystic artery"
]
}
},
"stop-parameter": {
"description": "Last
dissecting tool interaction with fibro-fatty tissue in Triangle of
Calot after the cystic artery has been skeletonized.",
"completion-of": {
"tool": [
"dissecting tool"
],
"action": [
"skeletonize"
],
"anatomy": [
"fibro-fatty
tissue in Triangle of Calot",
]
}
}
},
{
"name": "Dissection of Lower
1/3 of Gallbladder off Cystic Plate",
"description": "Dissection of
the lower 1/3 of gallbladder from the cystic plate.",
"start-parameter": {
"description": "First
dissecting tool interaction with lower 1/3 of the gallbladder with
intent to dissect the gallbladder off the cystic plate.",
"intent-to": {
"tool": [
"dissecting tool"
],
"action": [
"dissect"
],
"anatomy": [
"lower 1/3 of
gallbladder"
]
}
},
"stop-parameter": {
"description": "Last
dissecting tool interaction with lower 1/3 of the gallbladder after
gallbladder has been dissected off the cystic plate.",
"completion-of": {
"tool": [
```

```
                                        "dissecting tool"
                                    ],
                                    "action": [
                                        "dissect"
                                    ],
                                    "anatomy": [
                                        "lower 1/3 of the
GB"
                                    ]
                                }
                            }
                        }
                    ]
                },
                {
                    "name": "Dissection of Gallbladder off
Liver Bed",
                    "description": "Gallbladder is
dissected off the liver bed with intent to free the gallbladder from
the liver bed.",
                    "start-parameter": {
                        "description": "First dissecting
tool interaction with gallbladder with intent to dissect the
gallbladder off the liver bed.",
                        "intent-to": {
                            "tool": [
                                "dissecting tool"
                            ],
                            "action": [
                                "dissect"
                            ],
                            "anatomy": [
                                "gallbladder"
                            ]
                        }
                    },
                    "stop-parameter": {
                        "description": "Last dissecting
tool interaction with gallbladder resulting after the gallbladder has
been dissected off the liver bed.",
                        "completion-of": {
                            "tool": [
                                "dissecting tool"
                            ],
                            "action": [
                                "dissect"
                            ],
                            "anatomy": [
                                "gallbladder"
                            ]
```

```
                                    }
                                },
                                "tasks": []
                            }
                        ]
                    },
                    {
                        "name": "Transection",
                        "description": "Permanent division of target
anatomy into two distinct structures or distinct functional
compartments for access to subsequent target anatomy or in preparation
for reconstruction or extraction.",
                        "start-parameter": {
                            "description": "Mirrors start parameter of
first nested chronological segment annotated beneath Transection
phase.",
                            "intent-to": [
                                "tool",
                                "action",
                                "anatomy"
                            ]
                        },
                        "stop-parameter": {
                            "description": "Mirrors stop parameter of
last nested chronological segment annotated beneath Transection
phase.",
                            "completion-of": [
                                "tool",
                                "action",
                                "anatomy"
                            ]
                        },
                        "steps": [
                            {
                                "name": "L&T of Cystic Duct",
                                "description": "Cystic duct is ligated
and transected to isolate gallbladder from biliary tree.",
                                "start-parameter": {
                                    "description": "First
visualization of stapler or clip applier with intent to ligate and
transect the cystic duct.",
                                    "intent-to": {
                                        "tool": [
                                            "stapler",
                                            "clip applier"
                                        ],
                                        "action": [
                                            "ligate",
                                            "transect"
                                        ],
```

```
                                        "anatomy": [
                                            "cystic duct"
                                        ]
                                    }
                                },
                                "stop-parameter": {
                                    "description": "Last visualization
of stapler or last transecting tool interaction with cystic duct after
it has been ligated and transected.",
                                    "completion-of": {
                                        "tool": [
                                            "stapler",
                                            "transecting tool"
                                        ],
                                        "action": [
                                            "ligate",
                                            "transect"
                                        ],
                                        "anatomy": [
                                            "cystic duct"
                                        ]
                                    }
                                },
                                "tasks": []
                            },
                            {
                                "name": "L&T of Cystic Artery",
                                "description": "Cystic artery is
ligated and transected for vascular control.",
                                "start-parameter": {
                                    "description": "First
visualization of stapler or clip applier with intent to ligate and
transect the cystic artery.",
                                    "intent-to": {
                                        "tool": [
                                            "stapler",
                                            "clip applier"
                                        ],
                                        "action": [
                                            "ligate",
                                            "transect"
                                        ],
                                        "anatomy": [
                                            "cystic artery"
                                        ]
                                    }
                                },
                                "stop-parameter": {
                                    "description": "Last visualization
of stapler or last transecting tool interaction with cystic artery
```

```
after it has been ligated and transected.",
                                    "completion-of": {
                                        "tool": [
                                            "stapler",
                                            "transecting tool"
                                        ],
                                        "action": [
                                            "ligate",
                                            "transect"
                                        ],
                                        "anatomy": [
                                            "cystic artery"
                                        ]
                                    }
                                },
                                "tasks": []
                            },
                            {
                                "name": "L&T of Cystic Artery & Duct",
                                "description": "Cystic artery and
cystic duct are ligated and transected for vascular control and
isolation of gallbladder from biliary tree.",
                                "start-parameter": {
                                    "description": "First
visualization of stapler or clip applier with intent to ligate and
transect the cystic artery and duct.",
                                    "intent-to": {
                                        "tool": [
                                            "stapler",
                                            "clip applier"
                                        ],
                                        "action": [
                                            "ligate",
                                            "transect"
                                        ],
                                        "anatomy": [
                                            "cystic artery",
                                            "cystic duct"
                                        ]
                                    }
                                },
                                "stop-parameter": {
                                    "description": "Last visualization
of stapler or last transecting tool interaction with cystic artery and
duct after they have been ligated and transected.",
                                    "completion-of": {
                                        "tool": [
                                            "stapler",
                                            "transecting tool"
                                        ],
```

"action": [
"ligate",
"transect"
],
"anatomy": [
"cystic artery",
"cystic duct"
]
}
},
"tasks": []
}
]
},
{
"name": "Reconstruction",
"description": "Realignment of formerly exposed, dissected, or transected anatomy or buttressing of weakened structures to restore structural and/or physiological function.",
"start-parameter": {
"description": "Mirrors start parameter of first nested chronological segment annotated beneath Reconstruction phase.",
"intent-to": [
"tool",
"action",
"anatomy"
]
},
"stop-parameter": {
"description": "Mirrors stop parameter of last nested chronological segment annotated beneath Reconstruction phase.",
"completion-of": [
"tool",
"action",
"anatomy"
]
},
"steps": [
{
"name": "Hemostasis of Liver Bed",
"description": "Any bleeding from liver bed is noted and hemostasis is achieved.",
"start-parameter": {
"description": "First thermal dissection tool interaction with cystic plate with intent to achieve hemostasis.",
"intent-to": {
"tool": [

"thermal dissection tool"
],
"action": [
"achieve hemostasis"
],
"anatomy": [
"cystic plate"
]
}
},
"stop-parameter": {
"description": "Last thermal dissection tool interaction with cystic plate after hemostasis has been achieved.",
"completion-of": {
"tool": [
"thermal dissection tool"
],
"action": [
"achieve hemostasis"
],
"anatomy": [
"cystic plate"
]
}
},
"tasks": []
}
]
},
{
"name": "Extraction",
"description": "Collection and removal of isolated specimens from the body.",
"start-parameter": {
"description": "Mirrors start parameter of first nested chronological segment annotated beneath Extraction phase.",
"intent-to": [
"tool",
"action",
"anatomy"
]
},
"stop-parameter": {
"description": "Mirrors stop parameter of last nested chronological segment annotated beneath Extraction phase.",
"completion-of": [
"tool",

```
                                "action",
                                "anatomy"
                            ]
                        },
                        "steps": [
                            {
                                "name": "Extraction of Gallbladder",
                                "description": "Extraction of resected
gallbladder to remove specimen from the body.",
                                "start-parameter": {
                                    "description": "First grasping
tool interaction with gallbladder with intent to remove from the body
or place in specimen bag.",
                                    "intent-to": {
                                        "tool": [
                                            "grasping tool"
                                        ],
                                        "action": [
                                            "extract",
                                            "place in specimen bag"
                                        ],
                                        "anatomy": [
                                            "gallbladder"
                                        ]
                                    }
                                },
                                "stop-parameter": {
                                    "description": "Last visualization
of specimen or specimen bag containing GB.",
                                    "completion-of": {
                                        "tool": [
                                            "visualization tool"
                                        ],
                                        "action": [
                                            "extract"
                                        ],
                                        "anatomy": [
                                            "gallbladder"
                                        ]
                                    }
                                },
                                "tasks": []
                            }
                        ]
                    }
                ]
            }
        ]
    }
}
```
