# Supplementary material for: A Standardized Temporal Segmentation Framework and Annotation Resource Library in Robotic Surgery
Source: Mayo Clin Proc Digit Health. 2025 Aug 22;3(4):100257. doi: 10.1016/j.mcpdig.2025.100257 (PMC12492233; doi:10.1016/j.mcpdig.2025.100257)
Supplement: Supplementary Appendix 2 [file mmc2.pdf]

```
{
    "ontology": {
        "name": "SurgicalAnnotationOntology_LowAnteriorResection",
        "description": "A nested and customizable ontology for
annotating surgical activities in surgical videos.",
        "granularityLevels": [
            {
                "name": "Extended Phase",
                "description": "The least granular tier of temporal
segmentation in the surgical procedure."
            },
            {
                "name": "Step",
                "description": "The second hierarchical tier of
temporal segmentation, which define specific surgical intentions
through completion of surgical actions."
            },
            {
                "name": "Task",
                "description": "The third hierarchical tier of
temporal segmentation, which define specific surgical intentions
through completion of surgical actions."
            },
            {
                "name": "Subtask",
                "description": "The fourth hierarchical tier of
temporal segmentation, which define specific surgical intentions
through completion of surgical actions."
            }
        ],
        "Actions": [
            {
                "name": "Dissection",
                "description": "Using instruments to separate
anatomical structures."
            },
            {
                "name": "Transection",
                "description": "Using instruments to divide an
anatomical structure into two distinct structures."
            },
            {
                "name": "Ligation",
                "description": "Using instruments to tie off or
occlude anatomical structures, such as blood vessels."
            },
            {
                "name": "Retraction",
                "description": "Manipulation of tissue to improve
exposure."
```

```
            },
            {
                "name": "Mobilization",
                "description": "Using instruments to move or free
anatomical structures from surrounding tissue."
            },
            {
                "name": "Skeletonization",
                "description": "Using instruments to remove
surrounding tissue and expose underlying anatomical structures."
            },
            {
                "name": "Sweeping",
                "description": "Using instruments to gently move or
push anatomical structures, such as bowel or omentum, out of the way."
            },
            {
                "name": "Extraction",
                "description": "Using instruments to remove anatomical
structures, specimens, or other material from the body."
            },
            {
                "name": "Anastomosis",
                "description": "Using instruments to connect or join
anatomical structures, such as blood vessels or bowel segments."
            },
            {
                "name": "Hemostasis",
                "description": "Control of bleeding during surgery."
            },
            {
                "name": "Exploration",
                "description": "Using instruments to examine or
inspect anatomical structures."
            },
            {
                "name": "Installation",
                "description": "Insertion of instruments into the body
cavity."
            }
        ],
        "procedures": [
            {
                "name": "Low Anterior Resection",
                "description": "Surgical resection of the rectum
typically for rectal cancer.",
                "phases": [
                    {
                        "name": "Exposure",
                        "description": "Exploration and preparation of
```

```
the visual field with intent to expose target anatomy prior to
procedure type-specific surgical activities, including sweeping or
general dissection of non-target anatomy and/or removal of previous
surgical material.",
                        "start-parameter": {
                            "description": "Mirrors start parameter of
first nested chronological segment annotated beneath Exposure phase.",
                            "intent-to": [
                                "action",
                                "anatomy",
                                "tool"
                            ]
                        },
                        "stop-parameter": {
                            "description": "Mirrors stop parameter of
last nested chronological segment annotated beneath Exposure phase.",
                            "completion-of": [
                                "action",
                                "anatomy",
                                "tool"
                            ]
                        },
                        "steps": [
                            {
                                "name": "Tool Installation",
                                "description": "Installation of
robotic instruments through ports into the body cavity to prepare for
surgical activity.",
                                "start-parameter": {
                                    "description": "First
visualization of first tool as it is installed into the body cavity.",
                                    "intent-to": {
                                        "tool": [
                                            "first tool"
                                        ],
                                        "action": [
                                            "visualize",
                                        ],
                                        "anatomy": [
                                            "body cavity"
                                        ]
                                    }
                                },
                                "stop-parameter": {
                                    "description": "Last tool movement
immediately after last tool is installed into body cavity.",
                                    "completion-of": {
                                        "tool": [
                                            "last tool"
                                        ],
```

```
                                        "action": [
                                            "install"
                                        ],
                                        "anatomy": [
                                            "body cavity"
                                        ]
                                    }
                                },
                                "tasks": []
                            },
                            {
                                "name": "Initial Exposure",
                                "description": "Actions performed to
expose and assess target anatomy in preparation for procedure-specific
activities.",
                                "start-parameter": {
                                    "description": "First tool
interaction with solid organ, bowel, omentum, adhesions, or previous
surgical material with intent to expose and assess target anatomy in
preparation for dissection.",
                                    "intent-to": {
                                        "tool": [
                                            "tool"
                                        ],
                                        "action": [
                                            "expose",
                                            "assess"
                                        ],
                                        "anatomy": [
                                            "solid organ",
                                            "bowel",
                                            "omentum",
                                            "adhesions",
                                            "previous surgical
material"
                                        ]
                                    }
                                },
                                "stop-parameter": {
                                    "description": "Last tool
interaction with solid organ, bowel, omentum, adhesions, or previous
surgical material such that target anatomy is exposed, assessed, and
surgical field is ready for dissection.",
                                    "completion-of": {
                                        "tool": [
                                            "tool"
                                        ],
                                        "action": [
                                            "expose",
                                            "assess"
```

```
                        ],
                        "anatomy": [
                            "solid organ",
                            "bowel",
                            "omentum",
                            "adhesions",
                            "previous surgical
material"
                        ]
                    }
                },
                "tasks": [
                    {
                        "name": "Exploration of
Abdomen",
                        "description": "Exploration of
abdomen to assess pathology.",
                        "start-parameter": {
                            "description": "First
endoscope focus on abdominal anatomy with intent to explore anatomy
and assess pathology.",
                            "intent-to": {
                                "tool": [
                                    "endoscope"
                                ],
                                "action": [
                                    "explore",
                                    "assess"
                                ],
                                "anatomy": [
                                    "abdominal
anatomy"
                                ]
                            }
                        },
                        "stop-parameter": {
                            "description": "End of
exploratory endoscope movements across abdomen after pathology is
assessed.",
                            "completion-of": {
                                "tool": [
                                    "endoscope"
                                ],
                                "action": [
                                    "move",
                                    "assess"
                                ],
                                "anatomy": [
                                    "abdomen"
                                ]
```

```
                                        }
                                    }
                                },
                                {
                                    "name": "Bowel / Omentum
Sweep",
                                    "description": "Sweeping of
bowel or omentum with intent to expose target anatomy.",
                                    "start-parameter": {
                                        "description": "First tool
interaction with bowel or omentum with intent to expose target
anatomy.",
                                        "intent-to": {
                                            "tool": [
                                                "tool"
                                            ],
                                            "action": [
                                                "expose"
                                            ],
                                            "anatomy": [
                                                "bowel",
                                                "omentum"
                                            ]
                                        }
                                    },
                                    "stop-parameter": {
                                        "description": "Last tool
interaction with bowel or omentum to expose target anatomy.",
                                        "completion-of": {
                                            "tool": [
                                                "tool"
                                            ],
                                            "action": [
                                                "expose"
                                            ],
                                            "anatomy": [
                                                "bowel",
                                                "omentum"
                                            ]
                                        }
                                    }
                                },
                                {
                                    "name": "Lysis of Adhesions",
                                    "description": "Removal of
adhesions to prepare visual field and expose target anatomy.",
                                    "start-parameter": {
                                        "description": "First
dissecting tool interaction with adhesions with intent to expose
target anatomy.",
```

```
                                            "intent-to": {
                                                "tool": [
                                                    "dissecting tool"
                                                ],
                                                "action": [
                                                    "dissect"
                                                ],
                                                "anatomy": [
                                                    "adhesions"
                                                ]
                                            }
                                        },
                                        "stop-parameter": {
                                            "description": "Last
dissecting tool interaction with adhesions to expose target anatomy.",
                                            "completion-of": {
                                                "tool": [
                                                    "dissecting tool"
                                                ],
                                                "action": [
                                                    "expose",

           "dissect"
                                                ],
                                                "anatomy": [
                                                    "target anatomy",
                                                    "adhesions"
                                                ]
                                            }
                                        }
                                    }
                                ]
                            }
                        ]
                    },
                    {
                        "name": "Dissection",
                        "description": "Surgical activities to gain
access to and/or prepare target anatomy for subsequent transection,
reconstruction, and/or extraction. Target anatomy is separated or
mobilized to gain access to subsequent structures, typically along
natural tissue planes, or skeletonized or fully isolated from
surrounding structures without full division into two distinct
structures or distinct functional compartments.",
                        "start-parameter": {
                            "description": "Mirrors start parameter of
first nested chronological segment annotated beneath Dissection
phase.",
                            "intent-to": [
                                "action",
```

```
                                "anatomy",
                                "tool"
                            ]
                        },
                        "stop-parameter": {
                            "description": "Mirrors stop parameter of
last nested chronological segment annotated beneath Dissection
phase.",
                            "completion-of": [
                                "action",
                                "anatomy",
                                "tool"
                            ]
                        },
                        "steps": [
                            {
                                "name": "MD&S of Vascular Pedicle",
                                "description": "Dissection of
mesentery to skeletonize the vascular pedicle.",
                                "start-parameter": {
                                    "description": "First dissecting
tool interaction with mesentery with intent to dissect the mesentery
and skeletonize the vascular pedicle.",
                                    "intent-to": {
                                        "tool": [
                                            "dissecting tool"
                                        ],
                                        "action": [
                                            "dissect",
                                            "skeletonize"
                                        ],
                                        "anatomy": [
                                            "mesentery",
                                            "vascular pedicle"
                                        ]
                                    }
                                },
                                "stop-parameter": {
                                    "description": "Last dissecting
tool interaction with mesentery or perivascular tissue after the
vascular pedicle has been skeletonized.",
                                    "completion-of": {
                                        "tool": [
                                            "dissecting tool"
                                        ],
                                        "action": [
                                            "dissect",
                                                                "skele
tonize"
                                        ],
```

```
                                        "anatomy": [
                                            "mesentery",
                                            "perivascular tissue",
                                            "vascular pedicle"
                                        ]
                                    }
                                },
                                "tasks": [
                                    {
                                        "name": "MD&S of Inferior
Mesenteric Vessels",
                                        "description": "Dissection of
mesentery to skeletonize the inferior mesenteric vessels",
                                        "start-parameter": {
                                            "description": "First
dissecting tool interaction with mesentery with intent to dissect the
mesentery and skeletonize the inferior mesenteric vessels.",
                                            "intent-to": {
                                                "tool": [
                                                    "dissecting tool"
                                                ],
                                                "action": [
                                                    "dissect",
                                                    "skeletonize"
                                                ],
                                                "anatomy": [
                                                    "mesentery",
                                                    "inferior
mesenteric vessels"
                                                ]
                                            }
                                        },
                                        "stop-parameter": {
                                            "description": "Last
dissecting tool interaction with mesentery or perivascular tissue
after the inferior mesenteric vessels have been skeletonized.",
                                            "completion-of": {
                                                "tool": [
                                                    "dissecting tool"
                                                ],
                                                "action": [
                                                    "dissect",

          "skeletonize"
                                                ],
                                                "anatomy": [
                                                    "mesentery",
                                                    "perivascular
tissue",
```

"inferior mesenteric vessels"
]
}
}
},
{
"name": "MD&S of Left Colic
Vessels",
"description": "Dissection of
mesentery to skeletonize the left colic vessels.",
"start-parameter": {
"description": "First
dissecting tool interaction with mesentery with intent to dissect the
mesentery and skeletonize the left colic vessels.",
"intent-to": {
"tool": [
"dissecting tool"
],
"action": [
"dissect",
"skeletonize"
],
"anatomy": [
"mesentery",
"left colic
vessels"
]
}
},
"stop-parameter": {
"description": "Last
dissecting tool interaction with mesentery or perivascular tissue
after the left colic vessel has been skeletonized.",
"completion-of": {
"tool": [
"dissecting tool"
],
"action": [
"dissect",
"skeletonize"
],
"anatomy": [
"mesentery",
"perivascular
tissue",
"left colic vessels"
]
}

```
                                    }
                                },
                                {
                                    "name": "MD&S of Sigmoidal
Vessels",
                                    "description": "Dissection of
mesentery to skeletonize the sigmoidal vessels.",
                                    "start-parameter": {
                                        "description": "First
dissecting tool interaction with mesentery with intent to dissect the
mesentery and skeletonize the sigmoidal vessels.",
                                        "intent-to": {
                                            "tool": [
                                                "dissecting tool"
                                            ],
                                            "action": [
                                                "dissect",
                                                "skeletonize"
                                            ],
                                            "anatomy": [
                                                "mesentery",
                                                "sigmoidal
vessels"
                                            ]
                                        }
                                    },
                                    "stop-parameter": {
                                        "description": "Last
dissecting tool interaction with mesentery or perivascular tissue
after the sigmoidal vessels have been skeletonized.",
                                        "completion-of": {
                                            "tool": [
                                                "dissecting tool"
                                            ],
                                            "action": [
                                                "dissect",
                                                "skeletonize"
                                            ],
                                            "anatomy": [
                                                "mesentery",
                                                "perivascular
tissue",
                                                "sigmoidal
vessels"
                                            ]
                                        }
                                    }
                                },
                                {
                                    "name": "MD&S of Rectal
```

Vessels",
"description": "Dissection of mesentery to skeletonize the rectal vessels",
"start-parameter": {
"description": "First dissecting tool interaction with mesentery with intent to dissect the mesentery and skeletonize the rectal vessels.",
"intent-to": {
"tool": [
"dissecting tool"
],
"action": [
"dissect",
"skeletonize"
],
"anatomy": [
"mesentery",
"rectal vessels"
]
}
},
"stop-parameter": {
"description": "Last dissecting tool interaction with mesentery or perivascular tissue after the rectal vessels have been skeletonized.",
"completion-of": {
"tool": [
"dissecting tool"
],
"action": [
"skeletonize",
"dissect"
],
"anatomy": [
"mesentery",
"perivascular tissue",
"rectal vessels"
]
}
}
}
]
},
{
"name": "Mobilization of Colon",
"description": "A plane is created to mobilize the colon from its retroperitoneal attachments.",
"start-parameter": {

```
                                    "description": "First dissecting
tool interaction with mesentery, lateral attachments or ligaments of
the colon with intent to mobilize the colon.",
                                    "intent-to": {
                                        "tool": [
                                            "dissecting tool"
                                        ],
                                        "action": [
                                            "mobilize",
                                            "dissect"
                                        ],
                                        "anatomy": [
                                            "mesentery",
                                            "lateral attachments",
                                            "ligaments of the colon"
                                        ]
                                    }
                                },
                                "stop-parameter": {
                                    "description": "Last dissecting
tool interaction with mesentery, lateral attachments or ligaments of
the colon after the colon has been mobilized.",
                                    "completion-of": {
                                        "tool": [
                                            "dissecting tool"
                                        ],
                                        "action": [
                                            "mobilize"
                                        ],
                                        "anatomy": [
                                            "mesentery",
                                            "lateral attachments",
                                            "ligaments of the colon"
                                        ]
                                    }
                                },
                                "tasks": [
                                    {
                                        "name": "M-L Mobilization of
Rectum, Sigmoid, & Descending Colon",
                                        "description": "A plane is
created between the mesentery and the retroperitoneum by dissecting in
a M-L direction to mobilize the rectum, sigmoid, and descending
colon.",
                                        "start-parameter": {
                                            "description": "First
dissecting tool interaction with mesentery with intent to mobilize the
rectum, sigmoid and descending colon.",
                                            "intent-to": {
                                                "tool": [
```

```
                                        "dissecting tool"
                                    ],
                                    "action": [
                                        "mobilize"
                                    ],
                                    "anatomy": [
                                        "mesentery",
                                        "rectum",
                                        "sigmoid",
                                        "descending colon"
                                    ]
                                }
                            },
                            "stop-parameter": {
                                "description": "Last
dissecting tool interaction with mesentery after the rectum, sigmoid
and descending colon have been mobilized.",
                                "completion-of": {
                                    "tool": [
                                        "dissecting tool"
                                    ],
                                    "action": [
                                        "mobilize"
                                    ],
                                    "anatomy": [
                                        "mesentery",
                                        "rectum",
                                        "sigmoid",
                                        "descending colon"
                                    ]
                                }
                            }
                        },
                        {
                            "name": "L-M Mobilization of
Sigmoid & Descending Colon",
                            "description": "A plane is
created between the mesentery and the retroperitoneum by dissecting in
a L-M direction from the line of Toldt to mobilize the sigmoid and
descending colon.",
                            "start-parameter": {
                                "description": "First
dissecting tool interaction with lateral attachments at the line of
Toldt with intent to mobilize the sigmoid and descending colon.",
                                "intent-to": {
                                    "tool": [
                                        "dissecting tool"
                                    ],
                                    "action": [
                                        "mobilize",
```

```
                                        "dissect"
                                    ],
                                    "anatomy": [
                                        "sigmoid",
                                        "descending colon"
                                    ]
                                }
                            },
                            "stop-parameter": {
                                "description": "Last
dissecting tool interaction with lateral attachments after the sigmoid
and descending colon have been mobilized.",
                                "completion-of": {
                                    "tool": [
                                        "dissecting tool"
                                    ],
                                    "action": [
                                        "mobilize"
                                    ],
                                    "anatomy": [
                                        "sigmoid",
                                        "descending colon"
                                    ]
                                }
                            }
                        },
                        {
                            "name": "Mobilization of
Splenic Flexure",
                            "description": "The splenic
flexure is mobilized to lengthen the colon for a tension-free
anastomosis.",
                            "start-parameter": {
                                "description": "First
dissecting tool interaction with mesentery, lateral attachments or
ligaments of the splenic flexure with intent to mobilize the splenic
flexure.",
                                "intent-to": {
                                    "tool": [
                                        "dissecting tool"
                                    ],
                                    "action": [
                                        "mobilize"
                                    ],
                                    "anatomy": [
                                        "mesentery",
                                        "lateral
attachments",
                                        "ligaments of the
splenic flexure"
```

]
}
},
"stop-parameter": {
"description": "Last
dissecting tool interaction with mesentery, lateral attachments or
ligaments of the splenic flexure after the splenic flexure has been
mobilized.",
"completion-of": {
"tool": [
"dissecting tool"
],
"action": [
"dissect"
],
"anatomy": [
"mesentery",
"lateral
attachments",

"ligaments of the

splenic flexure"
]
}
}
}
]
}
]
},
{
"name": "Transection",
"description": "Permanent division of target
anatomy into two distinct structures or distinct functional
compartments for access to subsequent target anatomy or in preparation
for reconstruction or extraction.",
"start-parameter": {
"description": "Mirrors start parameter of
first nested chronological segment annotated beneath Transection
phase.",
"intent-to": [
"action",
"anatomy",
"tool"
]
},
"stop-parameter": {
"description": "Mirrors stop parameter of
last nested chronological segment annotated beneath Transection
phase.",
"completion-of": [

```
                        "action",
                        "anatomy",
                        "tool"
                    ]
                },
                "steps": [
                    {
                        "name": "L&T of Vascular Pedicle",
                        "description": "The vascular pedicle
is ligated and transected.",
                        "start-parameter": {
                            "description": "First
visualization of stapler or clip applier with intent to ligate and
transect the vascular pedicle.",
                            "intent-to": {
                                "tool": [
                                    "stapler",
                                    "clip applier"
                                ],
                                "action": [
                                    "visualize",
                                    "ligate",
                                    "transect"
                                ],
                                "anatomy": [
                                    "vascular pedicle"
                                ]
                            }
                        },
                        "stop-parameter": {
                            "description": "Last visualization
of stapler or last transecting tool interaction with vascular pedicle
after the vascular pedicle has been ligated and transected.",
                            "completion-of": {
                                "tool": [
                                    "stapler",
                                    "transecting tool"
                                ],
                                "action": [
                                    "visualize",
                                    "ligate",
                                    "transect"
                                ],
                                "anatomy": [
                                    "vascular pedicle"
                                ]
                            }
                        },
                        "tasks": [
                            {
```

```
                                        "name": "L&T of Inferior
Mesenteric Vessels",
                                        "description": "The inferior
mesenteric vessels are ligated and transected.",
                                        "start-parameter": {
                                            "description": "First
visualization of stapler or clip applier with intent to ligate and
transect the inferior mesenteric vessels.",
                                            "intent-to": {
                                                "tool": [
                                                    "stapler",
                                                    "clip applier"
                                                ],
                                                "action": [
                                                    "visualize",
                                                    "ligate",
                                                    "transect"
                                                ],
                                                "anatomy": [
                                                    "inferior
mesenteric vessels"
                                                ]
                                            }
                                        },
                                        "stop-parameter": {
                                            "description": "Last
visualization of stapler or last transecting tool interaction with
inferior mesenteric vessels after the inferior mesenteric vessels have
been ligated and transected.",
                                            "completion-of": {
                                                "tool": [
                                                    "stapler",
                                                    "transecting tool"
                                                ],
                                                "action": [
                                                    "visualize",
                                                    "ligate",
                                                    "transect"
                                                ],
                                                "anatomy": [
                                                    "inferior
mesenteric vessels"
                                                ]
                                            }
                                        }
                                    },
                                    {
                                        "name": "L&T of Left Colic
Vessels",
                                        "description": "The left colic
```

```
vessels are ligated and transected.",
                                        "start-parameter": {
                                            "description": "First
visualization of stapler or clip applier with intent to ligate and
transect the left colic vessels.",
                                            "intent-to": {
                                                "tool": [
                                                    "stapler",
                                                    "clip applier"
                                                ],
                                                "action": [
                                                    "visualize",
                                                    "ligate",
                                                    "transect"
                                                ],
                                                "anatomy": [
                                                    "left colic
vessels"
                                                ]
                                            }
                                        },
                                        "stop-parameter": {
                                            "description": "Last
visualization of stapler or last transecting tool interaction with
left colic vessels after the left colic vessels have been ligated and
transected.",
                                            "completion-of": {
                                                "tool": [
                                                    "stapler",
                                                    "transecting tool"
                                                ],
                                                "action": [
                                                    "interact",
                                                    "visualize"
                                                ],
                                                "anatomy": [
                                                    "left colic
vessels"
                                                ]
                                            }
                                        }
                                    },
                                    {
                                        "name": "L&T of Sigmoidal
Vessels",
                                        "description": "The sigmoidal
vessels are ligated and transected.",
                                        "start-parameter": {
                                            "description": "First
visualization of stapler or clip applier with intent to ligate and
```

```
divide the sigmoidal vessels.",
                                            "intent-to": {
                                                "tool": [
                                                    "stapler",
                                                    "clip applier"
                                                ],
                                                "action": [
                                                    "visualize",
                                                    "ligate",
                                                    "divide"
                                                ],
                                                "anatomy": [
                                                    "sigmoidal
vessels"
                                                ]
                                            }
                                        },
                                        "stop-parameter": {
                                            "description": "Last
visualization of stapler or last transecting tool interaction with
sigmoidal vessels after the sigmoidal vessels have been ligated and
transected.",
                                            "completion-of": {
                                                "tool": [
                                                    "stapler",
                                                    "transecting tool"
                                                ],
                                                "action": [
                                                    "transect"
                                                ],
                                                "anatomy": [
                                                    "sigmoidal
vessels"
                                                ]
                                            }
                                        }
                                    },
                                    {
                                        "name": "L&T of Rectal
Vessels",
                                        "description": "The rectal
vessels are ligated and transected.",
                                        "start-parameter": {
                                            "description": "First
visualization of stapler or clip applier with intent to ligate and
transect the rectal vessels.",
                                            "intent-to": {
                                                "tool": [
                                                    "stapler",
                                                    "clip applier"
```

```
                                                ],
                                                "action": [
                                                    "visualize",
                                                    "ligate",
                                                    "transect"
                                                ],
                                                "anatomy": [
                                                    "rectal vessels"
                                                ]
                                            }
                                        },
                                        "stop-parameter": {
                                            "description": "Last
visualization of stapler or last transecting tool interaction with
rectal vessels after the rectal vessels have been ligated and
transected.",
                                            "completion-of": {
                                                "tool": [
                                                    "stapler",
                                                    "transecting tool"
                                                ],
                                                "action": [
                                                    "interact"
                                                ],
                                                "anatomy": [
                                                    "rectal vessels"
                                                ]
                                            }
                                        }
                                    }
                                ]
                            },
                            {
                                "name": "Total Mesorectal Dissection &
Mobilization",
                                "description": "The rectum is
dissected circumferentially and mobilized together with surrounding
mesorectum as an intact fascial envelope.",
                                "start-parameter": {
                                    "description": "First dissecting
tool interaction with rectum with intent to dissect and mobilize the
mesorectum.",
                                    "intent-to": {
                                        "tool": [
                                            "dissecting tool"
                                        ],
                                        "action": [
                                            "dissect",
                                            "mobilize"
                                        ],
```

```
                                        "anatomy": [
                                            "mesorectum"
                                        ]
                                    }
                                },
                                "stop-parameter": {
                                    "description": "Last dissecting
tool interaction with the rectum after the rectum has been dissected
and mobilized.",
                                    "completion-of": {
                                        "tool": [
                                            "dissecting tool"
                                        ],
                                        "action": [
                                            "dissect",
                                            "mobilize"
                                        ],
                                        "anatomy": [
                                            "mesorectum"
                                        ]
                                    }
                                },
                                "tasks": [
                                    {
                                        "name": "Dissection &
Mobilization of Posterior Mesorectum",
                                        "description": "Posterior
dissection to mobilize posterior mesorectum.",
                                        "start-parameter": {
                                            "description": "First
dissecting tool interaction with posterior mesorectum with intent to
dissect and mobilize posterior mesorectum.",
                                            "intent-to": {
                                                "tool": [
                                                    "dissecting tool"
                                                ],
                                                "action": [
                                                    "dissect",

          "mobilize"
                                                ],
                                                "anatomy": [
                                                    "posterior
mesorectum"
                                                ]
                                            }
                                        },
                                        "stop-parameter": {
                                            "description": "Last
dissecting tool interaction with the posterior mesorectum after the
```

posterior mesorectum has been dissected and mobilized.",
"completion-of": {
"tool": [
"dissecting tool"
],
"action": [
"dissect",
"mobilize"
],
"anatomy": [
"posterior
mesorectum"
]
}
}
},
{
"name": "Dissection &
Mobilization of Right Lateral Mesorectum",
"description": "Right lateral
dissection to mobilize right lateral mesorectum.",
"start-parameter": {
"description": "First
dissecting tool interaction with right lateral mesorectum with intent
to dissect right lateral mesorectum.",
"intent-to": {
"tool": [
"dissecting tool"
],
"action": [
"dissect",

"mobilize"
],
"anatomy": [
"right lateral
mesorectum"
]
}
},
"stop-parameter": {
"description": "Last
dissecting tool interaction with the right lateral mesorectum after
the right lateral mesorectum has been dissected and mobilized",
"completion-of": {
"tool": [
"dissecting tool"
],
"action": [
"dissect",

"mobilize"
],
"anatomy": [
"right lateral
mesorectum"
]
}
}
},
{
"name": "Dissection &
Mobilization of Left Lateral Mesorectum",
"description": "Left lateral
dissection to mobilize left lateral mesorectum.",
"start-parameter": {
"description": "First
dissecting tool interaction with left lateral mesorectum with intent
to dissect left lateral mesorectum.",
"intent-to": {
"tool": [
"dissecting tool"
],
"action": [
"dissect",

"mobilize"
],
"anatomy": [
"left lateral
mesorectum"
]
}
},
"stop-parameter": {
"description": "Last
dissecting tool interaction with the left lateral mesorectum after the
left lateral mesorectum has been dissected and mobilized.",
"completion-of": {
"tool": [
"dissecting tool"
],
"action": [
"dissect",

"mobilize"
],
"anatomy": [
"left lateral
mesorectum"

                                                ]
                                            }
                                        }
                                    },
                                    {
                                        "name": "Dissection &
Mobilization of Anterior Mesorectum",
                                        "description": "Anterior
dissection to mobilize anterior mesorectum.",
                                        "start-parameter": {
                                            "description": "First
dissecting tool interaction with anterior mesorectum with intent to
dissect anterior mesorectum.",
                                            "intent-to": {
                                                "tool": [
                                                    "dissecting tool"
                                                ],
                                                "action": [
                                                    "dissect"

          "mobilize"
                                                ],
                                                "anatomy": [
                                                    "anterior
mesorectum"
                                                ]
                                            }
                                        },
                                        "stop-parameter": {
                                            "description": "Last
dissecting tool interaction with the anterior mesorectum after the
anterior mesorectum has been dissected and mobilized.",
                                            "completion-of": {
                                                "tool": [
                                                    "dissecting tool"
                                                ],
                                                "action": [
                                                    "mobilize",
                                                    "Dissect"
                                                ],
                                                "anatomy": [
                                                    "anterior
mesorectum"
                                                ]
                                            }
                                        }
                                    }
                                ]
                            },
                            {

```
                                "name": "Skeletonization of Proximal
Colon & Distal Rectum",
                                "description": "The proximal colon and
distal rectum are skeletonized.",
                                "start-parameter": {
                                    "description": "First dissecting
tool interaction with mesocolon or mesorectum with intent to
skeletonize the proximal colon and distal rectum.",
                                    "intent-to": {
                                        "tool": [
                                            "dissecting tool"
                                        ],
                                        "action": [
                                            "skeletonize"
                                        ],
                                        "anatomy": [
                                            "mesocolon",
                                            "mesorectum",
                                            "proximal colon",
                                            "distal rectum"
                                        ]
                                    }
                                },
                                "stop-parameter": {
                                    "description": "Last dissecting
tool interaction with mesocolon or mesorectum after the proximal colon
and distal rectum have been skeletonized.",
                                    "completion-of": {
                                        "tool": [
                                            "dissecting tool"
                                        ],
                                        "action": [
                                            "skeletonize"
                                        ],
                                        "anatomy": [
                                            "mesocolon",
                                            "mesorectum",
                                            "proximal colon",
                                            "distal rectum"
                                        ]
                                    }
                                },
                                "tasks": [
                                    {
                                        "name": "Skeletonization of
Distal Rectum",

                                        "description": "The distal
rectum is skeletonized.",
                                        "start-parameter": {
                                            "description": "First
```

```
dissecting tool interaction with mesorectum with intent to skeletonize
the distal rectum.",
                                            "intent-to": {
                                                "tool": [
                                                    "dissecting tool"
                                                ],
                                                "action": [
                                                    "Skeletonize"
                                                ],
                                                "anatomy": [
                                                    "mesorectum",
                                                    "distal rectum"
                                                ]
                                            }
                                        },
                                        "stop-parameter": {
                                            "description": "Last
dissecting tool interaction with mesorectum after the distal rectum
has been skeletonized.",
                                            "completion-of": {
                                                "tool": [
                                                    "dissecting tool"
                                                ],
                                                "action": [
                                                    "skeletonize"
                                                ],
                                                "anatomy": [
                                                    "mesorectum",
                                                    "distal rectum"
                                                ]
                                            }
                                        }
                                    },
                                    {
                                        "name": "Skeletonization of
Proximal Colon",
                                        "description": "The mesocolon
is divided circumferentially to skeletonize the proximal colon.",
                                        "start-parameter": {
                                            "description": "First
dissecting tool interaction with mesocolon with intent to skeletonize
the proximal colon.",
                                            "intent-to": {
                                                "tool": [
                                                    "dissecting tool"
                                                ],
                                                "action": [
                                                    "skeletonize"
                                                ],
                                                "anatomy": [
```

```
                                        "mesocolon",
                                        "proximal colon"
                                    ]
                                }
                            },
                            "stop-parameter": {
                                "description": "Last
dissecting tool interaction with mesocolon after the proximal colon
has been skeletonized.",
                                "completion-of": {
                                    "tool": [
                                        "dissecting tool"
                                    ],
                                    "action": [
                                        "skeletonize"
                                    ],
                                    "anatomy": [
                                        "mesocolon",
                                        "proximal colon"
                                    ]
                                }
                            }
                        }
                    ]
                },
                {
                    "name": "Transection of Colon &
Rectum",
                    "description": "The relevant portions
of the colon and rectum are transected.",
                    "start-parameter": {
                        "description": "First
visualization of stapler or clip applier with intent to transect the
colon or rectum.",
                        "intent-to": {
                            "tool": [
                                "visualization tool"
                            ],
                            "action": [
                                "transect"
                            ],
                            "anatomy": [
                                "colon",
                                "rectum"
                            ]
                        }
                    },
                    "stop-parameter": {
                        "description": "Last visualization
of stapler or last transecting tool interaction with the colon or
```

```
rectum after the relevant colorectal segment has been transected.",
                                    "completion-of": {
                                        "tool": [
                                            "stapler",
                                            "transecting tool"
                                        ],
                                        "action": [
                                            "transect"
                                        ],
                                        "anatomy": [
                                            "colon",
                                            "rectum"
                                        ]
                                    }
                                },
                                "tasks": [
                                    {
                                        "name": "Transection of Distal
Rectum",
                                        "description": "The relevant
portions of the distal rectum are transected.",
                                        "start-parameter": {
                                            "description": "First
visualization of stapler or clip applier with intent to transect the
distal rectum.",
                                            "intent-to": {
                                                "tool": [
                                                    "stapler",
                                                    "clip applier"
                                                ],
                                                "action": [
                                                    "transect"
                                                ],
                                                "anatomy": [
                                                    "distal rectum"
                                                ]
                                            }
                                        },
                                        "stop-parameter": {
                                            "description": "Last
visualization of stapler or last transecting tool interaction after
the relevant distal rectal segment has been transected.",
                                            "completion-of": {
                                                "tool": [
                                                    "stapler",
                                                    "transecting tool"
                                                ],
                                                "action": [
                                                    "transect"
                                                ],
```

```
                                                "anatomy": [
                                                    "distal colon",
                                                    "distal rectum"
                                                ]
                                            }
                                        }
                                    },
                                    {
                                        "name": "Transection of
Proximal Colon",
                                        "description": "The relevant
portions of the proximal colon are transected.",
                                        "start-parameter": {
                                            "description": "First
visualization of stapler or clip applier with intent to transect the
proximal colon.",
                                            "intent-to": {
                                                "tool": [
                                                    "stapler",
                                                    "clip applier"
                                                ],
                                                "action": [
                                                    "transect"
                                                ],
                                                "anatomy": [
                                                    "proximal colon"
                                                ]
                                            }
                                        },
                                        "stop-parameter": {
                                            "description": "Last
visualization of stapler or last transecting tool interaction with
proximal colon after the relevant proximal colon segment has been
transected.",
                                            "completion-of": {
                                                "tool": [
                                                    "stapler",
                                                    "transecting tool"
                                                ],
                                                "action": [
                                                    "transect"
                                                ],
                                                "anatomy": [
                                                    "proximal colon"
                                                ]
                                            }
                                        }
                                    }
                                ]
                            }
```

```
                        ]
                    },
                    {
                        "name": "Reconstruction",
                        "description": "Realignment of formerly
exposed, dissected, or transected anatomy or buttressing of weakened
structures to restore structural and/or physiological function.",
                        "start-parameter": {
                            "description": "Mirrors start parameter of
first nested chronological segment annotated beneath Reconstruction
phase.",
                            "intent-to": [
                                "action",
                                "anatomy",
                                "tool"
                            ]
                        },
                        "stop-parameter": {
                            "description": "Mirrors stop parameter of
last nested chronological segment annotated beneath Reconstruction
phase.",
                            "completion-of": [
                                "action",
                                "anatomy",
                                "tool"
                            ]
                        },
                        "steps": [
                            {
                                "name": "Creation of Colorectal
Anastomosis",
                                "description": "Bowel continuity is
restored through the creation of the colorectal anastomosis.",
                                "start-parameter": {
                                    "description": "First
visualization of circular stapler or anvil with intent to create the
colorectal anastomosis.",
                                    "intent-to": {
                                        "tool": [
                                            "circular stapler",
                                            "anvil"
                                        ],
                                        "action": [
                                            "visualize",
                                                                    "anast
omose"
                                        ],
                                        "anatomy": [
                                            "colorectal anatomy"
                                        ]
```

```
                                    }
                                },
                                "stop-parameter": {
                                    "description": "Last visualization
of distal displacement of rectum as the circular stapler is withdrawn
from rectum after the colorectal anastomosis has been created.",
                                    "completion-of": {
                                        "tool": [
                                            "circular stapler"
                                        ],
                                        "action": [
                                            "anatomose",
                                                                    "withd
rawal"
                                        ],
                                        "anatomy": [
                                            "colorectal anastamosis",
                                                                    "rectu
m"
                                        ]
                                    }
                                },
                                "tasks": []
                            }
                        ]
                    },
                    {
                        "name": "Extraction",
                        "description": "Collection and removal of
isolated specimens from the body.",
                        "start-parameter": {
                            "description": "Mirrors start parameter of
first nested chronological segment annotated beneath Extraction
phase.",
                            "intent-to": [
                                "action",
                                "anatomy",
                                "tool"
                            ]
                        },
                        "stop-parameter": {
                            "description": "Mirrors start parameter of
first nested chronological segment annotated beneath Extraction
phase.",
                            "completion-of": [
                                "action",
                                "anatomy",
                                "tool"
                            ]
                        },
```

```
                        "steps": [
                            {
                                "name": "Extraction of Colorectal
Specimen",
                                "description": "Extraction of resected
colorectal segment to remove specimen from the body.",
                                "start-parameter": {
                                    "description": "First grasping
tool interaction with colorectal specimen with intent to remove from
the body or place in specimen bag.",
                                    "intent-to": {
                                        "tool": [
                                            "grasping tool"
                                        ],
                                        "action": [
                                            "remove",
                                            "place"
                                        ],
                                        "anatomy": [
                                            "colorectal specimen"
                                        ]
                                    }
                                },
                                "stop-parameter": {
                                    "description": "Last visualization
of colorectal specimen or specimen bag containing colorectal
specimen.",
                                    "completion-of": {
                                        "tool": [
                                            "visualization tool"
                                        ],
                                        "action": [
                                            "visualize"
                                        ],
                                        "anatomy": [
                                            "colorectal specimen",
                                            "specimen bag"
                                        ]
                                    }
                                },
                                "tasks": []
                            }
                        ]
                    }
                ]
            }
        ]
    }
}
```
