# Supplementary material for: A Standardized Temporal Segmentation Framework and Annotation Resource Library in Robotic Surgery
Source: Mayo Clin Proc Digit Health. 2025 Aug 22;3(4):100257. doi: 10.1016/j.mcpdig.2025.100257 (PMC12492233; doi:10.1016/j.mcpdig.2025.100257)
Supplement: Supplementary Appendix 3 [file mmc3.pdf]

Supplementary Appendix 3

eTable 1. Information on Utilization of Cases, Procedure Types, and Case Durations……....…....…..2

eFigure 1. Validation of Annotation Practices with Inter-rater Variability……………...………….……...3

Description of Methodology for eFigure1 ....…………………...………………….…..……..4

**Procedure type**	**Total Cases**	**Average Video Time (HH:MM:SS)**	**Summed Video Time (HHHH:MM:SS)**
Cholecystectomy	3930	00:37:05	2429:52:30
Low Anterior Resection	410	02:36:19	1068:09:50
Gastric Bypass	643	01:36:29	1033:27:07
Sleeve Gastrectomy	314	00:59:42	312:28:48
Lobectomy	1017	01:27:46	1487:57:12
Inguinal Hernia Repair	3113	00:57:32	2982:01:52
Ventral Hernia Repair	1248	01:04:18	1337:47:44
Hysterectomy	962	01:17:01	1234:45:02
Hiatal Hernia Repair and Fundoplication	309	01:43:45	534:52:15
Radical Prostatectomy	1519	02:31:41	3839:36:36:19
**Summed Totals**	**13465**		**15227:31:32**

eTable 1. Total cases and case times by procedure type that have been annotated through this work.

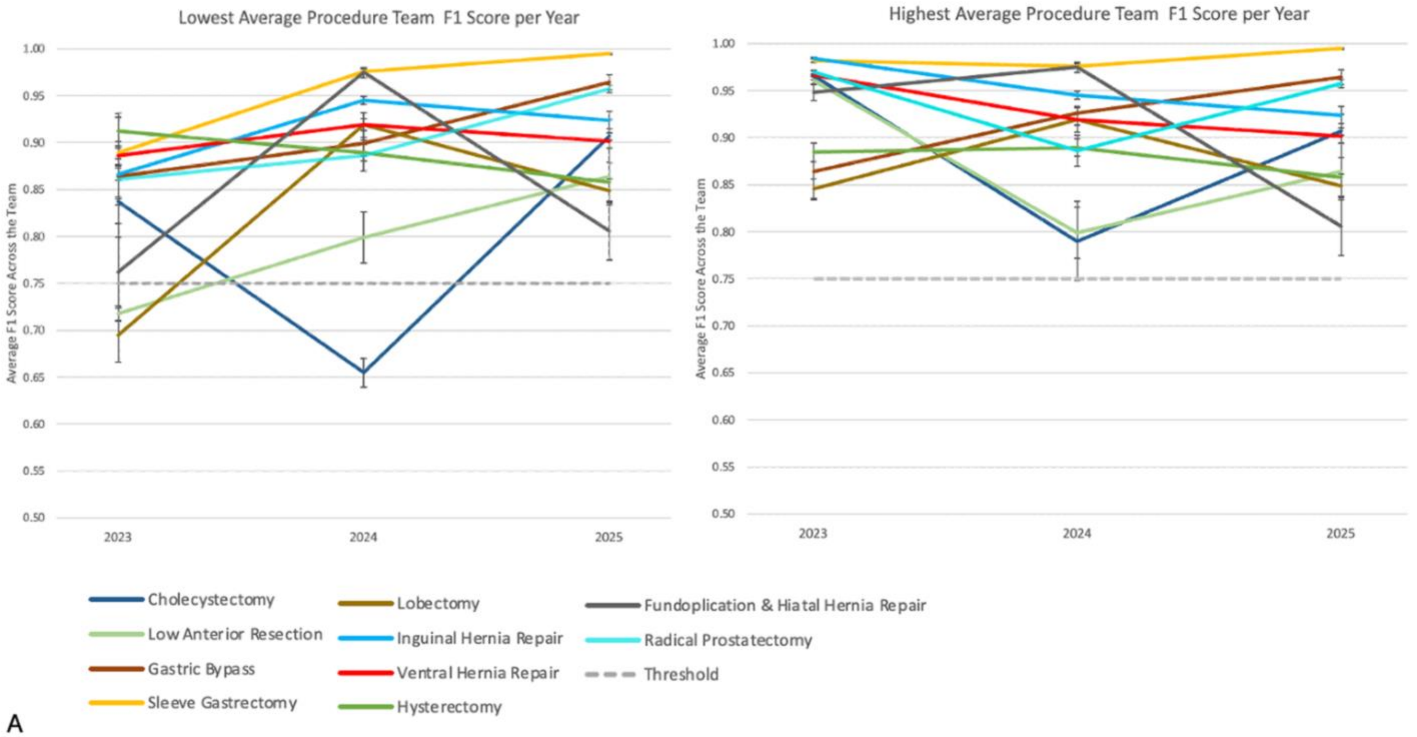

A

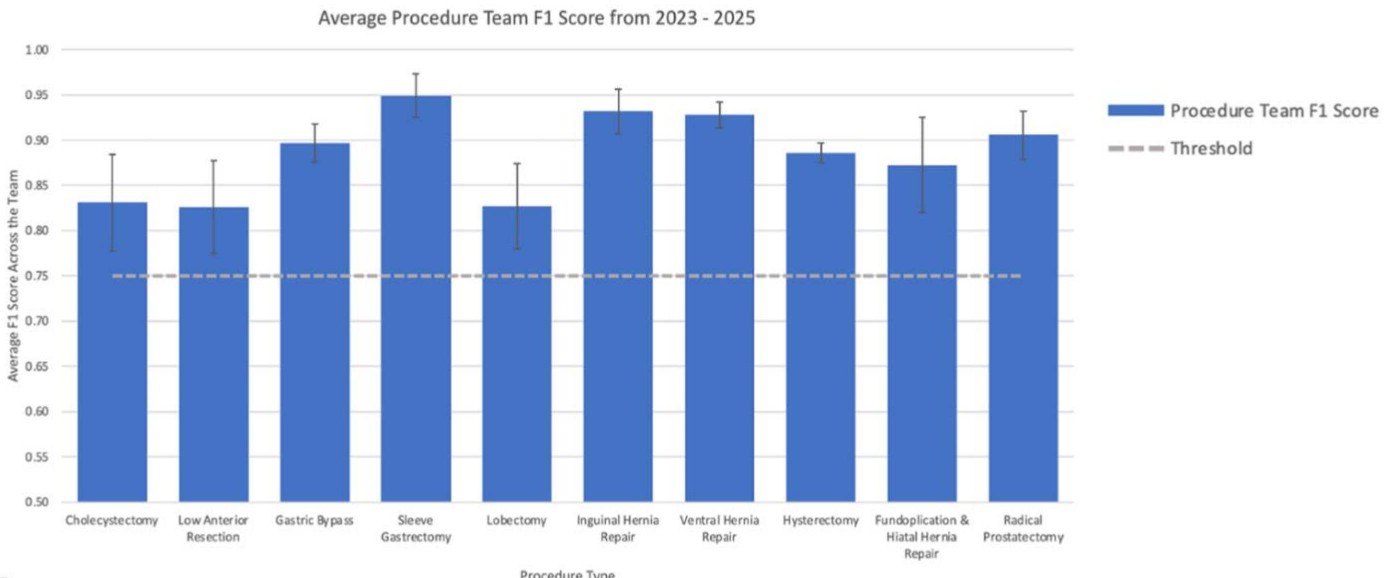

B

eFigure 1. Average procedure-specific annotation team F1 scores consistently remain above quality threshold. A) Lowest (left) and highest (right) average team F1 scores are plotted by year from 2023-2025. Up to 4 assessments are completed in any calendar year for each procedure-specific team, so both the lowest average F1 and highest are provided. Colors represent the 10 procedure types provided in the Annotation Resource Card library, and the grey dotted line represents the 0.75 quality threshold required without team retraining. B) Average F1 scores of all assessments are visualized by procedure type. Error bars for A, B represent standard error of the mean.

Description of Methodology:
Since 2023, all annotation teams (each specializing in specific procedure types) are asked to provide an optional quality assessment to ensure all annotations are aligned with the annotation cards. All teams are required to provide this assessment at least once annually. As such, up to four assessments may be completed for each procedure type team in any given calendar year. Each member of a procedure-specific team performs an individual annotation of a single selected case. The individual case is selected by the procedure-specific annotation team lead. Importantly, the selected case difficulty may vary from “standard” to “complex,” such that teams not only align on average cases but align on complex cases, as well. F1 scores for each case annotation are calculated for evaluation of rater-rater variability and as an average across the team. A threshold F1 score of 0.75 ensures annotation consistency. Regardless of score, after the annotation, each team compares their results as a group with in-depth discussion and video review for alignment while referencing the annotation card as ground truth. If any team scores below threshold (such as the cholecystectomy team in one 2024 assessment, and for which a particularly complex case was selected), all annotators on that team complete a retraining and realignment.
