# Supplementary material for: A Standardized Temporal Segmentation Framework and Annotation Resource Library in Robotic Surgery
Source: Mayo Clin Proc Digit Health. 2025 Aug 22;3(4):100257. doi: 10.1016/j.mcpdig.2025.100257 (PMC12492233; doi:10.1016/j.mcpdig.2025.100257)
Supplement: Supplementary Appendix 4 [file mmc4.pdf]

Supplementary Appendix 4: Temporal Annotation Resource Card Library

Table of Contents

BARIATRIC SURGERY, ROBOTIC-ASSISTED GASTRIC BYPASS 2

BARIATRIC SURGERY, ROBOTIC-ASSISTED SLEEVE GASTRECTOMY 6

CARDIOTHORACIC SURGERY, ROBOTIC-ASSISTED LOBECTOMY 8

COLORECTAL SURGERY, ROBOTIC-ASSISTED LOW ANTERIOR RESECTION 10

GENERAL SURGERY, ROBOTIC-ASSISTED CHOLECYSTECTOMY 16

GENERAL SURGERY, ROBOTIC-ASSISTED INGUINAL HERNIA REPAIR 19

GENERAL SURGERY, ROBOTIC-ASSISTED VENTRAL HERNIA REPAIR 21

GYNECOLOGIC SURGERY, ROBOTIC-ASSISTED HYSTERECTOMY 23

UPPER GASTROINTESTINAL SURGERY, ROBOTIC-ASSISTED HIATAL HERNIA REPAIR & FUNDOPLICATION 25

UROLOGIC SURGERY, ROBOTIC-ASSISTED RADICAL PROSTATECTOMY 29

Bariatric Surgery, Robotic-assisted Gastric Bypass, eTable 2

Ontology	Name	Surgical Objective	Start Parameter	Stop Parameter
Phase	Exposure	Exploration and preparation of the visual field with intent to expose target anatomy prior to procedure type-specific surgical activities, including sweeping or general dissection of non-target anatomy and/or removal of previous surgical material.	Mirrors start parameter of first nested chronological segment annotated beneath Exposure phase.	Mirrors stop parameter of last nested chronological segment annotated beneath Exposure phase.
Step	Tool Installation	Installation of robotic instruments through ports into the body cavity to prepare for surgical activity.	First visualization of first tool as it is installed into the body cavity.	Last tool movement immediately after last tool is installed into body cavity.
Step	Initial Exposure	Actions performed to expose and assess target anatomy in preparation for procedure-specific activities.	First tool interaction with solid organ, bowel, omentum, adhesions, or previous surgical material with intent to expose and assess target anatomy in preparation for dissection.	Last tool interaction with solid organ, bowel, omentum, adhesions, or previous surgical material such that target anatomy is exposed, assessed, and surgical field is ready for dissection.
Task	Exploration of Abdomen	Exploration of abdomen to assess pathology.	First endoscope focus on abdominal anatomy with intent to explore anatomy and assess pathology.	End of exploratory endoscope movements across abdomen after pathology is assessed.
Task	Bowel / Omentum Sweep	Sweeping of bowel or omentum with intent to expose target anatomy.	First tool interaction with bowel or omentum with intent to expose target anatomy.	Last tool interaction with bowel or omentum to expose target anatomy.
Task	Lysis of Adhesions	Removal of adhesions to prepare visual field and expose target anatomy.	First dissecting tool interaction with adhesions with intent to expose target anatomy.	Last dissecting tool interaction with adhesions to expose target anatomy.
Task	Retraction of Liver	Retraction of liver to facilitate adequate exposure of target anatomy.	First retracting tool interaction with liver with intent to expose target anatomy.	Last retracting tool interaction with liver after liver retraction to expose target anatomy.
Phase	Dissection	Surgical activities to gain access to and/or prepare target anatomy for subsequent transection, reconstruction, and/or extraction.*	Mirrors start parameter of first nested chronological segment annotated beneath Dissection phase.	Mirrors stop parameter of last nested chronological segment annotated beneath Dissection phase.
Step	Dissection of Gastrohepatic Ligament & Posterior Adhesions for Access to Lesser Curvature & Lesser Sac	Dissection of gastrohepatic ligament and posterior adhesions to obtain an adequate window for stapler access to the lesser curvature and lesser sac.	First dissecting tool interaction with intent to dissect the gastrohepatic ligament and posterior adhesions for access to the lesser curvature and lesser sac.	Last dissecting tool interaction with the gastrohepatic ligament or posterior adhesions after dissection of the gastrohepatic ligament and posterior adhesions such that access to the lesser curvature and lesser sac has been gained.
Step	Tunnelling Dissection of Posterior Adhesions & Gastrophrenic Ligament	Tunnelling dissection of posterior adhesions and gastrophrenic ligament to facilitate stapler pathway and exit.	First dissecting tool interaction with gastrophrenic ligament or posterior adhesions with intent to dissect the posterior adhesions and gastrophrenic ligament for facilitation of stapler passage.	Last dissecting tool interaction with the gastrophrenic ligament or posterior adhesions after the gastrophrenic ligament and posterior adhesions have been dissected such that a stapler pathway and exit have been established.

Task	Tunnelling Dissection of Posterior Adhesions	Tunneling dissection of posterior adhesions to facilitate stapler pathway.	First dissecting tool interaction with posterior adhesions with intent to dissect posterior adhesions to facilitate stapler pathway.	Last dissecting tool interaction with posterior adhesions after posterior adhesions have been dissected such that the stapler pathway has been established.
Task	Tunnelling Dissection of Gastrophrenic Ligament	Tunneling dissection of gastrophrenic ligament to facilitate stapler exit.	First dissecting tool interaction with gastrophrenic ligament with intent to dissect the gastrophrenic ligament to facilitate stapler exit.	Last dissecting tool interaction with gastrophrenic ligament after gastrophrenic ligament has been dissected such that stapler exit has been established.
Phase	Transection	Permanent division of target anatomy into two distinct structures or distinct functional compartments for access to subsequent target anatomy or in preparation for reconstruction or extraction.	Mirrors start parameter of first nested chronological segment annotated beneath Transection phase.	Mirrors stop parameter of last nested chronological segment annotated beneath Transection phase.
Step	Stapler Transection of Stomach	Linear stapler transection of the stomach.	First visualization of stapler with intent to transect the stomach.	Last visualization of stapler after stomach has been transected.
Phase	Reconstruction	Realignment of formerly exposed, dissected, or transected anatomy or buttressing of weakened structures to restore structural and/or physiological function.	Mirrors start parameter of first nested chronological segment annotated beneath Reconstruction phase.	Mirrors stop parameter of last nested chronological segment annotated beneath Reconstruction phase.
Step	Reinforcement of Gastric Staple Line	Suture reinforcement of gastric staple line to address or prevent bleeding.	First needle interaction with gastric staple line with intent to reinforce the gastric staple line.	Last cutting tool interaction with gastric staple line reinforcement suture after gastric staple line has been reinforced.
Task	Reinforcement of Gastric Pouch Staple Line	Suture reinforcement of gastric pouch staple line to address or prevent bleeding.	First needle interaction with gastric pouch staple line with intent to begin suture reinforcement.	Last cutting tool interaction with gastric pouch staple line reinforcement suture after gastric pouch staple line has been reinforced.
Task	Reinforcement of Gastric Remnant Staple Line	Suture reinforcement of gastric remnant staple line to address or prevent bleeding.	First needle interaction with gastric remnant staple line with intent to begin suture reinforcement.	Last cutting tool interaction with gastric remnant staple line reinforcement suture after gastric remnant staple line has been reinforced.
Step	Transection of Omentum	Transection of omentum into two separate compartments to create a tension-free passage (between the two separate compartments) for the alimentary limb as it passes anterior to the transverse colon and up to the gastric pouch.	First transecting tool interaction with greater omentum with intent to transect the omentum into two separate compartments.	Last transecting tool interaction with greater omentum after the omentum has been transected into two separate compartments.
Step	Measurement of Small Bowel	Measurement of small bowel to demarcate the optimal transection point.	First grasping tool interaction with small bowel with intent to measure and demarcate the optimal transection point.	Last grasping tool interaction with small bowel after the optimal transection point has been measured and demarcated.
Task	Measurement of PB Limb	Measurement of small bowel to demarcate the optimal transection point for the PB limb.	First grasping tool interaction with small bowel with intent to measure and demarcate the optimal transection point for the PB limb.	Last grasping tool interaction with small bowel after the optimal transection point for the PB limb has been measured and demarcated.

Task	Measurement of Alimentary Limb	Measurement of small bowel to demarcate the optimal transection point for the alimentary limb.	First grasping tool interaction with small bowel with intent to measure and demarcate the optimal transection point for the alimentary limb.	Last grasping tool interaction with small bowel after the optimal transection point for the alimentary limb has been measured and demarcated.
Step	Transection of Small Bowel	Small bowel is transected.	First visualization of stapler with intent to transect the small bowel.	Last visualization of stapler after the small bowel has been transected.
Step	Dissection of Small Bowel Mesentery	Dissection of small bowel mesentery.	First dissecting tool interaction with mesentery with intent to dissect the small bowel mesentery.	Last dissecting tool interaction with mesentery after the mesentery has been dissected.
Step	Tunnelling Dissection of Transverse Mesocolon	Tunnelling dissection of transverse mesocolon.	First dissecting tool interaction with transverse mesocolon with intent to facilitate path for bowel passage.	Last dissecting tool interaction with transverse mesocolon after path for bowel passage has been established.
Step	Creation of JJ** or GJ**	Anastomosis of PB and alimentary limbs to create the JJ, or alimentary limb and gastric pouch to create the GJ.	First visualization of stapler or needle interaction with bowel to place a stay suture with intent to create the JJ or GJ.	Last visualization of stapler or last cutting tool interaction with anastomotic creation suture after JJ or GJ has been created.
Task	Creation of JJ**	PB and alimentary limbs are anastomosed to create the JJ.	First visualization of stapler or needle interaction with bowel to place a stay suture with intent to create the JJ.	Last visualization of stapler or last cutting tool interaction with anastomotic creation suture after JJ has been created.
Task	Creation of GJ**	Alimentary limb and gastric pouch are anastomosed to create the GJ.	First visualization of stapler or needle interaction with bowel to place a stay suture with intent to create the GJ.	Last visualization of stapler or last cutting tool interaction with anastomotic creation suture after GJ has been created.
Task	Closure of Common JJ Enterotomy	Closure of the JJ enterotomy.	First visualization of stapler or first needle interaction with bowel with intent to close the common JJ enterotomy.	Last visualization of stapler or last cutting tool interaction with enterotomy closure suture after the common JJ enterotomy has been closed.
Task	Closure of Common GJ Enterotomy	Closure of the GJ enterotomy.	First visualization of stapler or first needle interaction with bowel with intent to close the common GJ enterotomy.	Last visualization of stapler or last cutting tool interaction with enterotomy closure suture after the common GJ enterotomy has been closed.
Step	Reinforcement of Anastomosis	Suture reinforcement to buttress the JJ and GJ with an additional layer of sutures.	First needle interaction with JJ or GJ with intent to reinforce the JJ or GJ with an additional suture layer.	Last cutting tool interaction with anastomotic reinforcement suture after JJ or GJ has been reinforced.
Task	Reinforcement of JJ	Suture reinforcement to buttress the JJ with an additional layer of sutures.	First needle interaction with JJ with intent to reinforce the JJ with an additional suture layer.	Last cutting tool interaction with anastomotic reinforcement suture after the JJ has been reinforced.
Task	Reinforcement of GJ	Suture reinforcement to buttress the GJ with an additional layer of sutures.	First needle interaction with GJ with intent to reinforce the GJ with an additional suture layer.	Last cutting tool interaction with anastomotic reinforcement suture after the GJ has been reinforced.
Step	Closure of Mesenteric Defect(s)	Closure of mesenteric defect(s) to prevent internal herniation of bowel.	First needle interaction with mesentery with intent to close the mesenteric defect.	Last cutting tool interaction with mesenteric defect closure suture after mesenteric defect has been closed.

Task	Closure of JJ Mesenteric Defect	Closure of JJ mesenteric defect to prevent internal herniation of bowel.	First needle interaction with mesentery underlying the JJ with intent to close the mesenteric defect.	Last cutting tool interaction with mesenteric defect closure suture after JJ mesenteric defect has been closed.
Task	Closure of Petersen's Space	Closure of Peterson's Space to prevent internal herniation of bowel.	First needle interaction with small bowel mesentery or transverse mesocolon with intent to close Petersen's space mesenteric defect.	Last cutting tool interaction with mesenteric defect closure suture after Petersen's space mesenteric defect has been closed.
Task	Closure of Transverse Mesocolic Defect	Closure of transverse mesocolic defect to prevent internal herniation of bowel.	First needle interaction with transverse mesocolic defect with intent to close the transverse mesocolic defect.	Last cutting tool interaction with mesenteric defect closure suture after transverse mesocolic defect has been closed.
Step	Suture of Omental Flap over GJ	Suturing of omental flap over the GJ to protect the anastomosis and contain potential leaks.	First needle interaction with omentum with intent to suture omental flap over the GJ.	Last cutting tool interaction with omental flap suture after omental flap has been placed and fixed.
Step	Repair of Inadvertent Bowel Enterotomy	Repair of unintentional bowel perforation.	First needle interaction with bowel with intent to repair the bowel enterotomy.	Last cutting tool interaction with enterotomy repair suture after the bowel enterotomy has been repaired.

eTable 2. Temporal annotation card specific to robotic-assisted gastric bypass. For each defined surgical segment, provided as its own row, the table includes the ontological granularity level, the segment name, its surgical objective, and the start and stop parameters for each. Shaded rows are the recommended annotation segments that balance clinical relevance and effort. *Indicates a truncated "Dissection" surgical objective, which is fully provided in Figure 2 of the manuscript. **Indicates hand sewn or stapled. Abbreviations: PB, pancreaticobiliary; GJ, gastrojejunostomy; JJ, jejunojejunostomy.

Bariatric Surgery, Robotic-assisted Sleeve Gastrectomy , eTable 3

Ontology	Name	Surgical Objective	Start Parameter	Stop Parameter
Phase	Exposure	Exploration and preparation of the visual field with intent to expose target anatomy prior to procedure type-specific surgical activities, including sweeping or general dissection of non-target anatomy and/or removal of previous surgical material.	Mirrors start parameter of first nested chronological segment annotated beneath Exposure phase.	Mirrors stop parameter of last nested chronological segment annotated beneath Exposure phase.
Step	Tool Installation	Installation of robotic instruments through ports into the body cavity to prepare for surgical activity.	First visualization of first tool as it is installed into the body cavity.	Last tool movement immediately after last tool is installed into body cavity.
Step	Initial Exposure	Actions performed to expose and assess target anatomy in preparation for procedure-specific activities.	First tool interaction with solid organ, bowel, omentum, adhesions, or previous surgical material with intent to expose and assess target anatomy in preparation for dissection.	Last tool interaction with solid organ, bowel, omentum, adhesions, or previous surgical material such that target anatomy is exposed, assessed, and surgical field is ready for dissection.
Task	Exploration of Abdomen	Exploration of abdomen to assess pathology.	First endoscope focus on abdominal anatomy with intent to explore anatomy and assess pathology.	End of exploratory endoscope movements across abdomen after pathology is assessed.
Task	Bowel / Omentum Sweep	Sweeping of bowel or omentum with intent to expose target anatomy.	First tool interaction with bowel or omentum with intent to expose target anatomy.	Last tool interaction with bowel or omentum to expose target anatomy.
Task	Lysis of Adhesions	Removal of adhesions to prepare visual field and expose target anatomy.	First dissecting tool interaction with adhesions with intent to expose target anatomy.	Last dissecting tool interaction with adhesions to expose target anatomy.
Task	Retraction of Liver	Retraction of liver to facilitate adequate exposure of target anatomy.	First retracting tool interaction with liver with intent to expose target anatomy.	Last retracting tool interaction with liver after liver has been retracted to expose target anatomy.
Phase	Dissection	Surgical activities to gain access to and/or prepare target anatomy for subsequent transection, reconstruction, and/or extraction.*	Mirrors start parameter of first nested chronological segment annotated beneath Dissection phase.	Mirrors stop parameter of last nested chronological segment annotated beneath Dissection phase.
Step	Dissection of Greater Omentum & Posterior Adhesions to Mobilize Stomach	Dissection of greater omentum (gastrocolic, gastrosplenic and gastrophrenic ligaments) and posterior adhesions to mobilize the stomach.	First dissecting tool interaction with greater omentum or posterior adhesions with intent to dissect the greater omentum and posterior adhesions and mobilize the stomach.	Last dissecting tool interaction with greater omentum or posterior adhesions after the greater omentum and posterior adhesions have been dissected and the stomach has been mobilized.
Task	Dissection of Greater Omentum to Mobilize Greater Curvature	Dissection of greater omentum to mobilize the greater curvature.	First dissecting tool interaction with greater omentum with intent to dissect the greater omentum and mobilize the greater curvature.	Last dissecting tool interaction with greater omentum after the greater omentum has been dissected and the greater curvature has been mobilized.

Subtask	Dissection of Gastrocolic Ligament	Dissection of gastrocolic ligament to free it from the greater curvature.	First dissecting tool interaction with gastrocolic ligament with intent to dissect the gastrocolic ligament and free it from the greater curvature.	Last dissecting tool interaction with gastrocolic ligament after the gastrocolic ligament has been dissected and freed from the greater curvature.
Subtask	Dissection of Gastrosplenic Ligament & Short Gastric Vessels	Dissection of gastrosplenic ligament which contains the short gastric vessels to free it from the greater curvature.	First dissecting tool interaction with gastrosplenic ligament with intent to dissect the gastrosplenic ligament and free it from the greater curvature.	Last dissecting tool interaction with gastrosplenic ligament after the gastrosplenic ligament has been dissected and freed from the greater curvature.
Subtask	Dissection of Gastrophrenic Ligament	Dissection of gastrophrenic ligament to free it from the greater curvature.	First dissecting tool interaction with gastrophrenic ligament with intent to dissect the gastrophrenic ligament and free it from the greater curvature.	Last dissecting tool interaction with gastrophrenic ligament after the gastrophrenic ligament has been dissected and freed from the greater curvature.
Task	Dissection of Posterior Adhesions	Dissection of posterior adhesions to mobilize the stomach.	First dissecting tool interaction with posterior adhesions with intent to dissect the posterior adhesions and mobilize the stomach.	Last dissecting tool interaction with posterior adhesions after the posterior adhesions have been dissected and the stomach has been mobilized.
Phase	Transection	Permanent division of target anatomy into two distinct structures or distinct functional compartments for access to subsequent target anatomy or in preparation for reconstruction or extraction.	Mirrors start parameter of first nested chronological segment annotated beneath Transection phase.	Mirrors stop parameter of last nested chronological segment annotated beneath Transection phase.
Step	Stapler Transection of Stomach	Linear stapler transection of the stomach.	First visualization of stapler with intent to transect the stomach.	Last visualization of stapler after the stomach has been transected.
Phase	Reconstruction	Realignment of formerly exposed, dissected, or transected anatomy or buttressing of weakened structures to restore structural and/or physiological function.	Mirrors start parameter of first nested chronological segment annotated beneath Reconstruction phase.	Mirrors stop parameter of last nested chronological segment annotated beneath Reconstruction phase.
Step	Reinforcement of Gastric Staple Line	Suture reinforcement of gastric staple line to address or prevent bleeding.	First needle interaction with gastric staple line with intent to reinforce the gastric staple line.	Last cutting tool interaction with gastric staple line reinforcement suture after gastric staple line has been reinforced.
Phase	Extraction	Collection and removal of isolated specimens from the body.	Mirrors start parameter of first nested chronological segment annotated beneath Extraction phase.	Mirrors stop parameter of last nested chronological segment annotated beneath Extraction phase.
Step	Extraction of Gastric Specimen	Extraction of resected gastric segment to remove specimen from the body.	First grasping tool interaction with gastric specimen with intent to remove from the body or place in specimen bag.	Last visualization of specimen or specimen bag containing gastric specimen.

eTable 3. Temporal annotation card specific to robotic-assisted sleeve gastrectomy. For each defined surgical segment, provided as its own row, the table includes the ontological granularity level, the segment name, its surgical objective, and the start and stop parameters for each. Shaded rows are the recommended annotation segments that balance clinical relevance and effort. *Indicates a truncated “Dissection” surgical objective, which is fully provided in Figure 2 of the manuscript.

Cardiothoracic Surgery, Robotic-assisted Lobectomy , eTable 4

Ontology	Name	Surgical Objective	Start Parameter	Stop Parameter
Phase	Exposure	Exploration and preparation of the visual field with intent to expose target anatomy prior to procedure type-specific surgical activities, including sweeping or general dissection of non-target anatomy and/or removal of previous surgical material.	Mirrors start parameter of first nested chronological segment annotated beneath Exposure phase.	Mirrors stop parameter of last nested chronological segment annotated beneath Exposure phase.
Step	Tool Installation	Installation of robotic instruments through ports into the body cavity to prepare for surgical activity.	First visualization of first tool as it is installed into the body cavity.	Last tool movement immediately after last tool is installed into body cavity.
Step	Initial Exposure	Actions performed to expose and assess target anatomy in preparation for procedure-specific activities.	First tool interaction with solid organ, bowel, omentum, adhesions, or previous surgical material with intent to expose and assess target anatomy in preparation for dissection.	Last tool interaction with solid organ, bowel, omentum, adhesions, or previous surgical material such that target anatomy is exposed, assessed, and surgical field is ready for dissection.
Task	Exploration of Thorax	Exploration of thorax to assess pathology.	First endoscope focus on thorax anatomy with intent to explore anatomy and assess pathology.	End of exploratory endoscope movements across thorax after pathology is assessed.
Task	Lysis of Adhesions	Removal of adhesions to prepare visual field and expose target anatomy.	First dissecting tool interaction with adhesions with intent to expose target anatomy.	Last dissecting tool interaction with adhesions to expose target anatomy.
Phase	Dissection	Surgical activities to gain access to and/or prepare target anatomy for subsequent transection, reconstruction, and/or extraction.*	Mirrors start parameter of first nested chronological segment annotated beneath Dissection phase.	Mirrors stop parameter of last nested chronological segment annotated beneath Dissection phase.
Step	Dissection of Inferior Pulmonary Ligament	Dissection of inferior pulmonary ligament.	First dissecting tool interaction with inferior pulmonary ligament with intent to dissect the inferior pulmonary ligament.	Last dissecting tool interaction with inferior pulmonary ligament after the inferior pulmonary ligament has been dissected.
Step	Dissection of Lymph Nodes	Dissection of lymph node(s) for staging purposes.	First dissecting tool interaction with lymph node or perinodal tissue with intent to dissect the lymph node(s).	Last dissecting tool interaction with lymph node or perinodal tissue after the lymph node(s) have been dissected.
Step	Dissection of Lung Fissure	Dissection of lung fissure.	First visualization of stapler or first dissecting tool interaction with lung fissure with intent to dissect the lung fissure.	Last visualization of stapler or last dissecting tool interaction with lung fissure after the lung fissure has been dissected.
Step	Skeletonization of Pulmonary Artery or Branches	Skeletonization of pulmonary artery or branches.	First dissecting tool interaction with perivascular tissue with intent to skeletonize the pulmonary artery and/or its branches.	Last dissecting tool interaction with perivascular tissue after the pulmonary artery and/or its branches have been skeletonized.
Step	Skeletonization of Pulmonary Vein or Tributaries	Skeletonization of pulmonary vein and/or its tributaries.	First dissecting tool interaction with perivascular tissue with intent to skeletonize the pulmonary vein and/or its tributaries.	Last dissecting tool interaction with perivascular tissue after the pulmonary vein and/or its tributaries have been skeletonized.

Step	Skeletonization of Lobar Bronchus	Skeletonization of lobar bronchus.	First dissecting tool interaction with peribronchial tissue with intent to skeletonize the lobar bronchus.	Last dissecting tool interaction with peribronchial tissue after the lobar bronchus has been skeletonized.
Phase	Transection	Permanent division of target anatomy into two distinct structures or distinct functional compartments for access to subsequent target anatomy or in preparation for reconstruction or extraction.	Mirrors start parameter of first nested chronological segment annotated beneath Transection phase.	Mirrors stop parameter of last nested chronological segment annotated beneath Transection phase.
Step	Transection of Pulmonary Artery or Branches	Pulmonary artery and its branches are transected.	First visualization of stapler or first transecting tool interaction with pulmonary artery or branches with intent to transect the pulmonary artery and/or its branches.	Last visualization of stapler or last transecting tool interaction with pulmonary artery or its branches after the pulmonary artery and/or its branches have been transected.
Step	Transection of Pulmonary Vein or Tributaries	Pulmonary vein and/or its tributaries are transected.	First visualization of stapler or first transecting tool interaction with pulmonary vein or its tributaries with intent to transect the pulmonary vein and/or its tributaries.	Last visualization of stapler or last transecting tool interaction with pulmonary vein and/ or its tributaries after the pulmonary vein and/or its tributaries have been transected.
Step	Transection of Lobar Bronchus	Lobar bronchus is transected.	First visualization of stapler or first transecting tool interaction with lobar bronchus with intent to transect the lobar bronchus.	Last visualization of stapler or last transecting tool interaction with lobar bronchus after the lobar bronchus has been transected.
Step	Wedge Transection	Transection of a wedge of lung tissue.	First visualization of stapler with intent to transect the wedge.	Last visualization of stapler after the wedge has been transected.
Phase	Extraction	Collection and removal of isolated specimens from the body.	Mirrors start parameter of first nested chronological segment annotated beneath Extraction phase.	Mirrors stop parameter of last nested chronological segment annotated beneath Extraction phase.
Step	Extraction of Lung Specimen	Extraction of resected lung segment or lobe to remove specimen from the body.	First grasping tool interaction with lung specimen with intent to remove from the body or place in specimen bag.	Last visualization of lung specimen or specimen bag containing lung specimen.
Step	Extraction of Lymph Nodes	Extraction of resected lymph node(s) to remove specimen from the body.	First grasping tool interaction with lymph node(s) with intent to remove from the body or place in specimen bag.	Last visualization of lymph node or specimen bag containing lymph node(s).

eTable 4. Temporal annotation card specific to robotic-assisted lobectomy. For each defined surgical segment, provided as its own row, the table includes the ontological granularity level, the segment name, its surgical objective, and the start and stop parameters for each. Shaded rows are the recommended annotation segments that balance clinical relevance and effort. *Indicates a truncated "Dissection" surgical objective, which is fully provided in Figure 2 of the manuscript.

Colorectal Surgery, Robotic-assisted Low Anterior Resection, eTable 5

Ontology	Name	Surgical Objective	Start Parameter	Stop Parameter
Phase	Exposure	Exploration and preparation of the visual field with intent to expose target anatomy prior to procedure type-specific surgical activities, including sweeping or general dissection of non-target anatomy and/or removal of previous surgical material.	Mirrors start parameter of first nested chronological segment annotated beneath Exposure phase.	Mirrors stop parameter of last nested chronological segment annotated beneath Exposure phase.
Step	Tool Installation	Installation of robotic instruments through ports into the body cavity.	First visualization of first tool as it is installed into the body cavity.	Last tool movement immediately after last tool is installed into body cavity.
Step	Initial Exposure	Actions performed to expose and assess target anatomy in preparation for procedure-specific activities.	First tool interaction with solid organ, bowel, omentum, adhesions, or previous surgical material with intent to expose and assess target anatomy in preparation for dissection.	Last tool interaction with solid organ, bowel, omentum, adhesions, or previous surgical material such that target anatomy is exposed, assessed, and surgical field is ready for dissection.
Task	Exploration of Abdomen	Exploration of abdomen to assess pathology.	First endoscope focus on abdominal anatomy with intent to explore anatomy and assess pathology.	End of exploratory endoscope movements across abdomen after pathology is assessed.
Task	Bowel / Omentum Sweep	Sweeping of bowel or omentum with intent to expose target anatomy.	First tool interaction with bowel or omentum with intent to expose target anatomy.	Last tool interaction with bowel or omentum to expose target anatomy.
Task	Lysis of Adhesions	Removal of adhesions to prepare visual field and expose target anatomy.	First dissecting tool interaction with adhesions with intent to expose target anatomy.	Last dissecting tool interaction with adhesions to expose target anatomy.
Phase	Dissection	Surgical activities to gain access to and/or prepare target anatomy for subsequent transection, reconstruction, and/or extraction*	Mirrors start parameter of first nested chronological segment annotated beneath Dissection phase.	Mirrors stop parameter of last nested chronological segment annotated beneath Dissection phase.
Step	MD&S of Vascular Pedicle	Dissection of mesentery to skeletonize the vascular pedicle.	First dissecting tool interaction with mesentery with intent to dissect the mesentery and skeletonize the vascular pedicle.	Last dissecting tool interaction with mesentery or perivascular tissue after the vascular pedicle has been skeletonized.
Task	MD&S of Inferior Mesenteric Vessels	Dissection of mesentery to skeletonize the inferior mesenteric vessels	First dissecting tool interaction with mesentery with intent to dissect the mesentery and skeletonize	Last dissecting tool interaction with mesentery or perivascular tissue after the inferior mesenteric vessels have been skeletonized.

			the inferior mesenteric vessels.	
Subtask	MD&S of IMA	Dissection of mesentery to skeletonize the IMA.	First dissecting tool interaction with mesentery with intent to dissect the mesentery and skeletonize the IMA.	Last dissecting tool interaction with mesentery or perivascular tissue after the IMA has been skeletonized.
Subtask	MD&S of IMV	Dissection of mesentery to skeletonize the IMV.	First dissecting tool interaction with mesentery with intent to dissect the mesentery and skeletonize the IMV.	Last dissecting tool interaction with mesentery or perivascular tissue after the IMV has been skeletonized.
Task	MD&S of Left Colic Vessels	Dissection of mesentery to skeletonize the left colic vessels.	First dissecting tool interaction with mesentery with intent to dissect the mesentery and skeletonize the left colic vessels.	Last dissecting tool interaction with mesentery or perivascular tissue after the left colic vessel has been skeletonized.
Subtask	MD&S of LCA	Dissection of mesentery to skeletonize the LCA.	First dissecting tool interaction with mesentery with intent to dissect the mesentery and skeletonize the LCA.	Last dissecting tool interaction with mesentery or perivascular tissue after the LCA has been skeletonized.
Subtask	MD&S of LCV	Dissection of mesentery to skeletonize the LCV.	First dissecting tool interaction with mesentery with intent to dissect the mesentery and skeletonize the LCV.	Last dissecting tool interaction with mesentery or perivascular tissue after the LCV has been skeletonized.
Task	MD&S of Sigmoidal Vessels	Dissection of mesentery to skeletonize the sigmoidal vessels.	First dissecting tool interaction with mesentery with intent to dissect the mesentery and skeletonize the sigmoidal vessels.	Last dissecting tool interaction with mesentery or perivascular tissue after the sigmoidal vessels have been skeletonized.
Subtask	MD&S of SA	Dissection of mesentery to skeletonize the SA.	First dissecting tool interaction with mesentery with intent to dissect the mesentery and skeletonize the SA.	Last dissecting tool interaction with mesentery or perivascular tissue after the SA has been skeletonized.
Subtask	MD&S of SV	Dissection of mesentery to skeletonize the SV.	First dissecting tool interaction with mesentery with intent to dissect the mesentery and skeletonize the SV.	Last dissecting tool interaction with mesentery or perivascular tissue after the SV has been skeletonized.
Task	MD&S of Rectal Vessels	Dissection of mesentery to skeletonize the rectal vessels	First dissecting tool interaction with mesentery with intent to dissect the mesentery and skeletonize the rectal vessels.	Last dissecting tool interaction with mesentery or perivascular tissue after the rectal vessels have been skeletonized.
Subtask	MD&S or RA	Dissection of mesentery to skeletonize the RA.	First dissecting tool interaction with mesentery with intent to dissect the mesentery and skeletonize the RA.	Last dissecting tool interaction with mesentery or perivascular tissue after the RA has been skeletonized.
Subtask	MD&S of RV	Dissection of mesentery to skeletonize the RV.	First dissecting tool interaction with mesentery with intent to dissect the mesentery and skeletonize the RV.	Last dissecting tool interaction with mesentery or perivascular tissue after the RV has been skeletonized.
Step	Mobilization of Colon	A plane is created to mobilize the colon from its retroperitoneal attachments.	First dissecting tool interaction with mesentery, lateral attachments or ligaments of the colon with	Last dissecting tool interaction with mesentery, lateral attachments or

			intent to mobilize the colon.	ligaments of the colon after the colon has been mobilized.
Task	M-L Mobilization of Rectum, Sigmoid, & Descending Colon	A plane is created between the mesentery and the retroperitoneum by dissecting in a M-L direction to mobilize the rectum, sigmoid, and descending colon.	First dissecting tool interaction with mesentery with intent to mobilize the rectum, sigmoid and descending colon.	Last dissecting tool interaction with mesentery after the rectum, sigmoid and descending colon have been mobilized.
Subtask	M-L Mobilization of Rectum	A plane is created between the mesentery and the retroperitoneum by dissecting in a M-L direction to mobilize the rectum.	First dissecting tool interaction with mesentery with intent to mobilize the rectum.	Last dissecting tool interaction with mesentery after the rectum has been mobilized.
Subtask	M-L Mobilization of Sigmoid Colon	A plane is created between the mesentery and the retroperitoneum by dissecting in a M-L direction to mobilize the sigmoid colon.	First dissecting tool interaction with mesentery with intent to mobilize the sigmoid colon.	Last dissecting tool interaction with mesentery after the sigmoid colon has been mobilized.
Subtask	M-L Mobilization of Descending Colon	A plane is created between the mesentery and the retroperitoneum by dissecting in a M-L direction to mobilize the descending colon.	First dissecting tool interaction with mesentery with intent to mobilize the descending colon.	Last dissecting tool interaction with mesentery after the descending colon has been mobilized.
Task	L-M Mobilization of Sigmoid & Descending Colon	A plane is created between the mesentery and the retroperitoneum by dissecting in a L-M direction from the line of Toldt to mobilize the sigmoid and descending colon.	First dissecting tool interaction with lateral attachments at the line of Toldt with intent to mobilize the sigmoid and descending colon.	Last dissecting tool interaction with lateral attachments after the sigmoid and descending colon have been mobilized.
Subtask	L-M Mobilization of Sigmoid Colon	A plane is created between the mesentery and the retroperitoneum by dissecting in a L-M direction from the line of Toldt to mobilize the sigmoid colon.	First dissecting tool interaction with lateral attachments at the line of Toldt with intent to mobilize the sigmoid colon.	Last dissecting tool interaction with lateral attachments after the sigmoid colon has been mobilized.
Subtask	L-M Mobilization of Descending Colon	A plane is created between the mesentery and the retroperitoneum by dissecting in a L-M direction from the line of Toldt to mobilize the descending colon.	First dissecting tool interaction with lateral attachments at the line of Toldt with intent to mobilize the descending colon.	Last dissecting tool interaction with lateral attachments after the descending colon has been mobilized.
Task	Mobilization of Splenic Flexure	The splenic flexure is mobilized to lengthen the colon.	First dissecting tool interaction with mesentery, lateral attachments or ligaments of the splenic flexure with intent to mobilize the splenic flexure.	Last dissecting tool interaction with mesentery, lateral attachments or ligaments of the splenic flexure after the splenic flexure has been mobilized.
Subtask	Submesocolic Mobilization of Splenic Flexure	The submesocolic approach is employed to mobilize the splenic flexure and lengthen the colon.	First dissecting tool interaction with mesentery of the splenic flexure with intent to mobilize the splenic flexure.	Last dissecting tool interaction with submesocolic mesentery of the splenic flexure after the splenic flexure has been mobilized.
Subtask	L-M Mobilization of Splenic Flexure	The L-M approach is employed to mobilize the splenic flexure and lengthen the colon.	First dissecting tool interaction with lateral attachments or ligaments of the splenic flexure with intent to mobilize the splenic flexure.	Last dissecting tool interaction with lateral attachments or ligaments of the splenic flexure after the splenic flexure has been mobilized.

Subtask	Supracolic Mobilization of Splenic Flexure	The supracolic approach is employed to mobilize the splenic flexure and lengthen the colon.	First dissecting tool interaction with mesentery or ligaments of the splenic flexure with intent to mobilize the splenic flexure.	Last dissecting tool interaction with supracolic mesentery or ligaments of the splenic flexure after the splenic flexure has been mobilized.
Phase	Transection	Permanent division of target anatomy into two distinct structures or distinct functional compartments for access to subsequent target anatomy or in preparation for reconstruction or extraction.	Mirrors start parameter of first nested chronological segment annotated beneath Transection phase.	Mirrors stop parameter of last nested chronological segment annotated beneath Transection phase.
Step	L&T of Vascular Pedicle	The vascular pedicle is ligated and transected.	First visualization of stapler or clip applier with intent to ligate and transect the vascular pedicle.	Last visualization of stapler or last transecting tool interaction with vascular pedicle after the vascular pedicle has been ligated and transected.
Task	L&T of Inferior Mesenteric Vessels	The inferior mesenteric vessels are ligated and transected.	First visualization of stapler or clip applier with intent to ligate and transect the inferior mesenteric vessels.	Last visualization of stapler or last transecting tool interaction with inferior mesenteric vessels after the inferior mesenteric vessels have been ligated and transected.
Subtask	L&T of IMA	The IMA is ligated and transected.	First visualization of stapler or clip applier with intent to ligate and transect the IMA.	Last visualization of stapler or last transecting tool interaction with IMA after the IMA has been ligated and transected.
Subtask	L&T of IMV	The IMV is ligated and transected.	First visualization of stapler or clip applier with intent to ligate and transect the IMV.	Last visualization of stapler or last transecting tool interaction with IMV after the IMV has been ligated and transected.
Task	L&T of Left Çolic Vessels	The left colic vessels are ligated and transected.	First visualization of stapler or clip applier with intent to ligate and transect the left colic vessels.	Last visualization of stapler or last transecting tool interaction with left colic vessels after the left colic vessels have been ligated and transected.
Subtask	L&T of LCA	The LCA is ligated and transected.	First visualization of stapler or clip applier with intent to ligate and transect the LCA.	Last visualization of stapler or last transecting tool interaction with LCA after the LCA has been ligated and divided.
Subtask	L&T of LCV	The LCV is ligated and transected.	First visualization of stapler or clip applier with intent to ligate and transect the LCV.	Last visualization of stapler or last transecting tool interaction with LCV after the LCV has been ligated and transected.
Task	L&T of Sigmoidal Vessels	The sigmoidal vessels are ligated and transected.	First visualization of stapler or clip applier with intent to ligate and divide the sigmoidal vessels.	Last visualization of stapler or last transecting tool interaction with sigmoidal vessels after the sigmoidal vessels have been ligated and transected.
Subtask	L&T of SA	The SA is ligated and transected.	First visualization of stapler or clip applier with intent to ligate and transect the SA.	Last visualization of stapler or last transecting tool interaction with SA after the

				SA has been ligated and transected.
Subtask	L&T of SV	The SV is ligated and transected.	First visualization of stapler or clip applier with intent to ligate and transect the SV.	Last visualization of stapler or last transecting tool interaction with SV after the SV has been ligated and transected.
Task	L&T of Rectal Vessels	The rectal vessels are ligated and transected.	First visualization of stapler or clip applier with intent to ligate and transect the rectal vessels.	Last visualization of stapler or last transecting tool interaction with rectal vessels after the rectal vessels have been ligated and transected.
Subtask	L&T of RA	The RA is ligated and transected.	First visualization of stapler or clip applier with intent to ligate and transect the RA.	Last visualization of stapler or last transecting tool interaction with RA after the RA has been ligated and transected.
Subtask	L&T of RV	The RV is ligated and transected.	First visualization of stapler or clip applier with intent to ligate and transect the RV.	Last visualization of stapler or last transecting tool interaction with RV after the RV has been ligated and transected.
Step	Total Mesorectal Dissection & Mobilization	The rectum is dissected circumferentially and mobilized together with surrounding mesorectum as an intact fascial envelope.	First dissecting tool interaction with mesorectum with intent to dissect the mesorectum.	Last dissecting tool interaction with mesorectum after the rectum and mesorectum have been dissected and mobilized together.
Task	Dissection & Mobilization of Posterior Mesorectum	Posterior dissection to mobilize posterior mesorectum.	First dissecting tool interaction with posterior mesorectum with intent to dissect the posterior mesorectum.	Last dissecting tool interaction with posterior mesorectum after the posterior mesorectum has been dissected and mobilized.
Task	Dissection & Mobilization of Right Lateral Mesorectum	Right lateral dissection to mobilize right lateral mesorectum.	First dissecting tool interaction with right lateral mesorectum with intent to dissect the right lateral mesorectum.	Last dissecting tool interaction with right lateral mesorectum after the right lateral mesorectum has been dissected and mobilized.
Task	Dissection & Mobilization of Left Lateral Mesorectum	Left lateral dissection to mobilize left lateral mesorectum.	First dissecting tool interaction with left lateral mesorectum with intent to dissect the left lateral mesorectum.	Last dissecting tool interaction with left lateral mesorectum after the left lateral mesorectum has been dissected and mobilized.
Task	Dissection & Mobilization of Anterior Mesorectum	Anterior dissection to mobilize anterior mesorectum.	First dissecting tool interaction with anterior mesorectum with intent to dissect the anterior mesorectum.	Last dissecting tool interaction with anterior mesorectum after the anterior mesorectum has been dissected and mobilized.
Step	Skeletonization of Proximal Colon & Distal Rectum	The proximal colon and distal rectum are skeletonized.	First dissecting tool interaction with mesocolon or mesorectum with intent to skeletonize the proximal colon and distal rectum.	Last dissecting tool interaction with mesocolon or mesorectum after the proximal colon and distal rectum have been skeletonized.
Task	Skeletonization of Distal Rectum	The distal rectum is skeletonized.	First dissecting tool interaction with mesorectum with intent to skeletonize the distal rectum.	Last dissecting tool interaction with mesorectum after the distal rectum has been skeletonized.

Task	Skeletonization of Proximal Colon	The proximal colon is skeletonized.	First dissecting tool interaction with mesocolon with intent to skeletonize the proximal colon.	Last dissecting tool interaction with mesocolon after the proximal colon has been skeletonized.
Step	Transection of Colon & Rectum	The relevant portions of colon and rectum are transected.	First visualization of stapler or clip applier with intent to transect the colon or rectum.	Last visualization of stapler or last transecting tool interaction with colon or rectum after the relevant colorectal segment has been transected.
Task	Transection of Distal Rectum	The relevant portions of the distal rectum are transected.	First visualization of stapler or clip applier with intent to transect the distal rectum.	Last visualization of stapler or last transecting tool interaction with distal colon after the relevant distal rectal segment has been transected.
Task	Transection of Proximal Colon	The relevant portions of the proximal colon are transected.	First visualization of stapler or clip applier with intent to transect the proximal colon.	Last visualization of stapler or last transecting tool interaction with proximal colon after the relevant proximal colon segment has been transected.
Phase	Reconstruction	Realignment of formerly exposed, dissected, or transected anatomy or buttressing of weakened structures to restore structural and/or physiological function.	Mirrors start parameter of first nested chronological segment annotated beneath Reconstruction phase.	Mirrors stop parameter of last nested chronological segment annotated beneath Reconstruction phase.
Step	Creation of Colorectal Anastomosis	Bowel continuity is restored through the creation of the colorectal anastomosis.	First visualization of circular stapler or anvil with intent to create the colorectal anastomosis.	Last visualization of distal displacement of rectum as the circular stapler is withdrawn from rectum after the colorectal anastomosis has been created.
Phase	Extraction	Collection and removal of isolated specimens from the body.	Mirrors start parameter of first nested chronological segment annotated beneath Extraction phase.	Mirrors stop parameter of last nested chronological segment annotated beneath Extraction phase.
Step	Extraction of Colorectal Specimen	Extraction of resected colorectal segment to remove specimen from the body.	First grasping tool interaction with colorectal specimen with intent to remove from the body or place in specimen bag.	Last visualization of colorectal specimen or specimen bag containing colorectal specimen.

eTable 5. Temporal annotation card specific to robotic-assisted low anterior resection. For each defined surgical segment, provided as its own row, the table includes the ontological granularity level, the segment name, its surgical objective, and the start and stop parameters for each. Shaded rows are the recommended annotation segments that balance clinical relevance and effort. *Indicates a truncated "Dissection" surgical objective, which is fully provided in Figure 2 of the manuscript. Abbreviations: MD&S, mesenteric dissection and skeletonization; IMA, inferior mesenteric artery; IMV, inferior mesenteric vein; LCA, left colic artery; LCV, left colic vein; SA, sigmoid artery; SV, sigmoid vein; RA, rectal artery; RV, rectal vein; M-L, medial to lateral; L-M, lateral to medial; L&T, ligation and transection.

General Surgery, Robotic-assisted Cholecystectomy, eTable 6

Ontology	Name	Surgical Objective	Start Parameter	Stop Parameter
Phase	Exposure	Exploration and preparation of the visual field with intent to expose target anatomy prior to procedure type-specific surgical activities, including sweeping or general dissection of non-target anatomy and/or removal of previous surgical material.	Mirrors start parameter of first nested chronological segment annotated beneath Exposure phase.	Mirrors stop parameter of last nested chronological segment annotated beneath Exposure phase.
Step	Tool Installation	Installation of robotic instruments through ports into the body cavity to prepare for surgical activity.	First visualization of first tool as it is installed into the body cavity.	Last tool movement immediately after last tool is installed into body cavity.
Step	Initial Exposure	Actions performed to expose and assess target anatomy in preparation for procedure-specific activities.	First tool interaction with solid organ, bowel, omentum, adhesions, or previous surgical material with intent to expose and assess target anatomy in preparation for dissection.	Last tool interaction with solid organ, bowel, omentum, adhesions, or previous surgical material such that target anatomy is exposed, assessed, and surgical field is ready for dissection.
Task	Exploration of Abdomen	Exploration of abdomen to assess pathology.	First endoscope focus on abdominal anatomy with intent to explore anatomy and assess pathology.	End of exploratory endoscope movements across abdomen after pathology is assessed.
Task	Bowel / Omentum Sweep	Sweeping of bowel or omentum with intent to expose target anatomy.	First tool interaction with bowel or omentum with intent to expose target anatomy.	Last tool interaction with bowel or omentum to expose target anatomy.
Task	Lysis of Adhesions	Removal of adhesions to prepare visual field and expose target anatomy.	First dissecting tool interaction with adhesions with intent to expose target anatomy.	Last dissecting tool interaction with adhesions to expose target anatomy.
Task	Retraction of Gallbladder	Retraction of the gallbladder to facilitate adequate exposure of target anatomy.	First retracting tool interaction with gallbladdder with intent to expose target anatomy.	Last retracting tool interaction with gallbladder after gallbladder has been retracted to expose target anatomy.
Phase	Dissection	Surgical activities to gain access to and/or prepare target anatomy for subsequent transection, reconstruction, and/or extraction.*	Mirrors start parameter of first nested chronological segment annotated beneath Dissection phase.	Mirrors stop parameter of last nested chronological segment annotated beneath Dissection phase.
Step	Dissection of Triangle of Calot	Dissection of structures within Triangle of Calot to visualize the critical view of safety.	First dissecting tool interaction with gallbladder infundibulum with intent to dissect Triangle of Calot.	Last dissecting tool interaction with Triangle of Calot after the Triangle of Calot has been dissected such that the critical view of safety is visible.
Task	Skeletonization of Cystic Duct	Skeletonization of cystic duct from surrounding fibro-fatty tissues.	First dissecting tool interaction with fibro-fatty tissue in the Triangle of Calot with intent to skeletonize the cystic duct.	Last dissecting tool interaction with fibro-fatty tissue in the Triangle of Calot after the cystic duct has been skeletonized.

Task	Skeletonization of Cystic Artery	Skeletonization of cystic artery from surrounding fibro-fatty tissues.	First dissecting tool interaction with fibro-fatty tissue in the Triangle of Calot with intent to skeletonize the cystic artery.	Last dissecting tool interaction with fibro-fatty tissue in the Triangle of Calot after the cystic artery has been skeletonized.
Task	Dissection of Lower 1/3 of Gallbladder off Cystic Plate	Dissection of the lower 1/3 of gallbladder from the cystic plate.	First dissecting tool interaction with lower 1/3 of the gallbladder with intent to dissect the gallbladder off the cystic plate.	Last dissecting tool interaction with lower 1/3 of the gallbladder after the gallbladder has been dissected off the cystic plate.
Phase	Transection	Permanent division of target anatomy into two distinct structures or distinct functional compartments for access to subsequent target anatomy or in preparation for reconstruction or extraction.	Mirrors start parameter of first nested chronological segment annotated beneath Transection phase.	Mirrors stop parameter of last nested chronological segment annotated beneath Transection phase.
Step	L&T of Cystic Duct	Cystic duct is ligated and transected to isolate gallbladder from biliary tree.	First visualization of stapler or clip applier with intent to ligate and transect the cystic duct.	Last visualization of stapler or last transecting tool interaction with cystic duct after the cystic duct has been ligated and transected.
Step	L&T of Cystic Artery	Cystic artery is ligated and transected for vascular control.	First visualization of stapler or clip applier with intent to ligate and transect the cystic artery.	Last visualization of stapler or last transecting tool interaction with cystic artery after the cyctic artery has been ligated and transected.
Step	L&T of Cystic Artery & Duct	Cystic artery and cystic duct are ligated and transected for vascular control and isolation of gallbladder from biliary tree.	First visualization of stapler or clip applier with intent to ligate and transect the cystic artery and duct.	Last visualization of stapler or last transecting tool interaction with cystic artery and duct after the cystic duct and cystic artery have been ligated and transected.
Step	Dissection of Gallbladder off Liver Bed	Gallbladder is dissected off the liver bed with intent to free the gallbladder from the liver bed.	First dissecting tool interaction with gallbladder with intent to dissect the gallbladder off the liver bed.	Last dissecting tool interaction with gallbladder resulting after the gallbladder has been dissected off the liver bed.
Phase	Reconstruction	Realignment of formerly exposed, dissected, or transected anatomy or buttressing of weakened structures to restore structural and/or physiological function.	Mirrors start parameter of first nested chronological segment annotated beneath Reconstruction phase.	Mirrors stop parameter of last nested chronological segment annotated beneath Reconstruction phase.
Step	Hemostasis of Liver Bed	Any bleeding from liver bed is noted and hemostasis is achieved.	First thermal dissection tool interaction with cystic plate with intent to achieve hemostasis.	Last thermal dissection tool interaction with cystic plate after hemostasis has been achieved.
Phase	Extraction	Collection and removal of isolated specimens from the body.	Mirrors start parameter of first nested chronological segment annotated beneath Extraction phase.	Mirrors stop parameter of last nested chronological segment annotated beneath Extraction phase.
Step	Extraction of Gallbladder	Extraction of resected gallbladder to remove specimen from the body.	First grasping tool interaction with gallbladder with intent to remove from the body or place in specimen bag.	Last visualization of specimen or specimen bag containing gallbladder.

eTable 6. Temporal annotation card specific to robotic-assisted cholecystectomy. For each defined surgical segment, provided as its own row, the table includes the ontological granularity level, the segment name, its surgical objective, and the start and stop parameters for each. Shaded rows are the recommended annotation segments that balance clinical relevance and effort. *Indicates a truncated "Dissection" surgical objective, which is fully provided in Figure 2 of the manuscript. Abbreviations: L&T, ligation and transection.

General Surgery, Robotic-assisted Inguinal Hernia Repair , eTable 7

Ontology	Name	Surgical Objective	Start Parameter	Stop Parameter
Phase	Exposure	Exploration and preparation of the visual field with intent to expose target anatomy prior to procedure type-specific surgical activities, including sweeping or general dissection of non-target anatomy and/or removal of previous surgical material.	Mirrors start parameter of first nested chronological segment annotated beneath Exposure phase.	Mirrors stop parameter of last nested chronological segment annotated beneath Exposure phase.
Step	Tool Installation	Installation of robotic instruments through ports into the body cavity to prepare for surgical activity.	First visualization of first tool as it is installed into the body cavity.	Last tool movement immediately after last tool is installed into body cavity.
Step	Initial Exposure	Actions performed to expose and assess target anatomy in preparation for procedure-specific activities.	First dissecting tool interaction with solid organ, bowel, omentum, adhesions, or previous surgical material with intent to expose and assess target anatomy in preparation for dissection.	Last tool interaction with solid organ, bowel, omentum, adhesions, or previous surgical material such that target anatomy is exposed, assessed, and surgical field is ready for dissection.
Task	Exploration of Pelvis	Exploration of pelvic region to assess pathology.	First endoscope focus on pelvic anatomy with intent to explore anatomy and assess pathology.	End of exploratory endoscope movements across pelvis after pelvis has been explored and pathology is assessed.
Task	Bowel / Omentum Sweep	Sweeping of bowel or omentum with intent to expose target anatomy.	First tool interaction with bowel or omentum with intent to expose target anatomy.	Last tool interaction with bowel or omentum to expose target anatomy.
Task	Lysis of Adhesions	Removal of adhesions to prepare visual field and expose target anatomy.	First dissecting tool interaction with adhesions with intent to expose target anatomy.	Last dissecting tool interaction with adhesions to expose target anatomy.
Task	Clearance of Existing Mesh**	Clearance of mesh from previous hernia repair.	First dissecting tool interaction with existing mesh with intent to clear the existing mesh from the surgical field.	Last dissecting tool interaction with existing mesh after it is cleared from the surgical field.
Phase	Dissection	Surgical activities to gain access to and/or prepare target anatomy for subsequent transection, reconstruction, and/or extraction. *	Mirrors start parameter of first nested chronological segment annotated beneath Dissection phase.	Mirrors stop parameter of last nested chronological segment annotated beneath Dissection phase.
Step	Incision of Peritoneum	The peritoneum is incised to facilitate peritoneal flap exploration.	First dissecting tool interaction with peritoneum with intent to make a peritoneal incision.	Last dissecting tool interaction with peritoneum after the peritoneal incision has been made.
Step	Exploration of Peritoneal Flap & Reduction of Hernia**	Exploration of peritoneal flap and reduction of hernia.	First dissecting tool interaction with peritoneal flap or hernia with intent to explore the peritoneal flap and reduce the hernia.	Last dissecting tool interaction with peritoneal flap or hernia after peritoneal flap has been explored and the hernia has been reduced.
Task	Exploration of Peritoneal Flap**	Exploration of the peritoneal flap.	First dissecting tool interaction with peritoneal flap tissue with intent to explore the peritoneal flap.	Last dissecting tool interaction with peritoneal flap after the peritoneal flap has been explored.
Task	Reduction of Hernia**	The hernia is reduced.	First dissecting tool interaction with hernia with intent to reduce the hernia.	Last dissecting tool interaction with hernia after the hernia has been reduced.
Phase	Reconstruction	Realignment of formerly exposed, dissected, or transected anatomy or buttressing of weakened structures to	Mirrors start parameter of first nested chronological segment	Mirrors stop parameter of last nested chronological segment annotated beneath Reconstruction phase.

		restore structural and/or physiological function.	annotated beneath Reconstruction phase.	
Step	Measurement of Hernia Defect**	Tape measurement of hernia defect to determine mesh sizing.	First grasping tool interaction with tape measure with intent to measure the hernia defect.	Last grasping tool interaction with tape measure after hernia defect has been measured.
Step	Placement & Fixation of Mesh over Myopectineal Orifice**	Mesh is placed over the myopectineal orifice to reinforce weakened myopectineal orifice and fixed with a tacking device, glue or suture to prevent folding or migration.	First grasping tool interaction with mesh with intent to place and fix the mesh over the myopectineal orifice.	Last grasping tool interaction with mesh, tack or glue or last cutting tool interaction with mesh fixation suture after mesh has been fixed over the myopectineal orifice.
Task	Placement of Mesh over Myopectineal Orifice**	Mesh is placed over the myopectineal orifice to reinforce weakened myopectineal orifice.	First grasping tool interaction with mesh with intent to place the mesh over the myopectineal orifice.	Last grasping tool interaction with mesh after mesh has been placed over the myopectineal orifice.
Task	Fixation of Mesh over Myopectineal Orifice**	Mesh is fixed over the myopectineal orifice to prevent folding or migration.	First visualization of tacking or glueing device or needle interaction with mesh with intent to fix the mesh over the myopectineal orifice.	Last visualization of tacking or glueing device or last cutting tool interaction with mesh fixation suture after mesh has been fixed over the myopectineal orifice.
Step	Closure of Peritoneum**	Peritoneum is reapproximated to close the peritoneum.	First needle interaction with peritoneum with intent to close the peritoneum.	Last cutting tool interaction with peritoneal closure suture after the peritoneum has been closed.

eTable 7. Temporal annotation card specific to robotic-assisted inguinal hernia repair. For each defined surgical segment, provided as its own row, the table includes the ontological granularity level, the segment name, its surgical objective, and the start and stop parameters for each. Shaded rows are the recommended annotation segments that balance clinical relevance and effort. *Indicates a truncated “Dissection” surgical objective, which is fully provided in Figure 2 of the manuscript. ** indicates right, left, and bilateral options.

General Surgery, Robotic-assisted Ventral Hernia Repair, eTable 8

Ontology	Name	Surgical Objective	Start Parameter	Stop Parameter
Phase	Exposure	Exploration and preparation of the visual field with intent to expose target anatomy prior to procedure type-specific surgical activities, including sweeping or general dissection of non-target anatomy and/or removal of previous surgical material.	Mirrors start parameter of first nested chronological segment annotated beneath Exposure phase.	Mirrors stop parameter of last nested chronological segment annotated beneath Exposure phase.
Step	Tool Installation	Installation of robotic instruments through ports into the body cavity to prepare for surgical activity	First visualization of first tool as it is installed into the body cavity.	Last tool movement immediately after last tool is installed into body cavity.
Step	Initial Exposure	Actions performed to expose and assess target anatomy in preparation for procedure-specific activities.	First tool interaction with solid organ, bowel, omentum, adhesions, or previous surgical material with intent to expose and assess target anatomy in preparation for dissection.	Last tool interaction with solid organ, bowel, omentum, adhesions, or previous surgical material such that target anatomy is exposed, assessed, and surgical field is ready for dissection.
Task	Exploration of Abdomen	Exploration of abdomen to assess pathology.	First endoscope focus on abdominal anatomy with intent to explore anatomy and assess pathology.	End of exploratory endoscope movements across abdomen after pathology is assessed.
Task	Bowel / Omentum Sweep	Sweeping of bowel or omentum with intent to expose target anatomy.	First tool interaction with bowel or omentum with intent to expose target anatomy.	Fast tool interaction with bowel or omentum to expose target anatomy.
Task	Lysis of Adhesions	Removal of adhesions to prepare visual field and expose target anatomy.	First dissecting tool interaction with adhesions with intent to expose target anatomy.	Last dissecting tool interaction with adhesions to expose target anatomy.
Task	Excision of Existing Mesh	Clearance of mesh from previous hernia repair.	First dissecting tool interaction with existing mesh with intent to clear the existing mesh from the surgical field.	Last dissecting tool interaction with existing mesh after it is cleared from the surgical field.
Phase	Dissection	Surgical activities to gain access to and/or prepare target anatomy for subsequent transection, reconstruction, and/or extraction.*	Mirrors start parameter of first nested chronological segment annotated beneath Dissection phase.	Mirrors stop parameter of last nested chronological segment annotated beneath Dissection phase.
Step	Incision of Peritoneum	The peritoneum is incised to facilitate peritoneal flap exploration.	First dissecting tool interaction with peritoneum with intent to make a peritoneal incision.	Last dissecting tool interaction with peritoneum after the peritoneal incision has been made.
Step	Exploration of Peritoneal Flap & Reduction of Hernia	Exploration of peritoneal flap and reduction of hernia.	First dissecting tool interaction with peritoneal flap or hernia with intent to explore the peritoneal flap and reduce the hernia.	Last dissecting tool interaction with peritoneal flap or hernia after peritoneal flap has been explored and the hernia has been reduced.
Task	Exploration of Peritoneal Flap	Exploration of the peritoneal flap.	First dissecting tool interaction with peritoneal flap tissue with intent to explore the peritoneal flap.	Last dissecting tool interaction with peritoneal flap tissue after the peritoneal flap has been explored.
Task	Reduction of Hernia	The hernia is reduced.	First dissecting tool interaction with hernia to reduce the hernia.	Last dissecting tool interaction with hernia after the hernia has been reduced.
Phase	Reconstruction	Realignment of formerly exposed, dissected, or transected anatomy or	Mirrors start parameter of first nested chronological segment	Mirrors stop parameter of last nested chronological segment annotated beneath Reconstruction phase.

		buttressing of weakened structures to restore structural and/or physiological function.	annotated beneath Reconstruction phase.	
Step	Creation of Fascial Flap	Mobilization of posterior rectus fascia to create fascial flap.	First dissecting tool interaction with peritoneum with intent to incise peritoneum and posterior rectus fascia to create fascial flap.	Last dissecting tool interaction with posterior rectus fascia after the fascial flap is created.
Task	Creation of Ipsilateral Fascial Flap	Mobilization of ipsilateral posterior rectus fascia to create ipsilateral fascial flap.	First dissecting tool interaction with peritoneum with intent to incise peritoneum and posterior rectus fascia to create ipsilateral facial flap.	Last dissecting tool interaction with posterior rectus fascia after the ipsilateral fascial flap is created.
Task	Creation of Contralateral Fascial Flap	Mobilization of contralateral posterior rectus fascia to create contralateral fascial flap.	First dissecting tool interaction with peritoneum with intent to incise peritoneum and posterior rectus fascia to create contralateral facial flap.	Last dissecting tool interaction with posterior rectus fascia after the contralateral fascial flap is created.
Task	Creation of Circumferential Fascial Flap	Circumferential mobilization of posterior rectus fascia to create circumferential fascial flap.	First dissecting tool interaction with peritoneum with intent to incise peritoneum and posterior rectus fascia to create circumferential facial flap.	Last dissecting tool interaction with posterior rectus fascia after the circumferential fascial flap is created.
Step	Measurement of Hernia Defect	Tape measurement of hernia defect to determine mesh sizing.	First grasping tool interaction with tape measure with intent to measure the hernia defect.	Last grasping tool interaction with tape measure after hernia defect has been measured.
Step	Closure of Hernia Defect	Primary suture closure of fascia to repair hernia defect.	First needle interaction with hernia defect with intent to close the hernia defect.	Last cutting tool interaction with hernia closure suture after hernia defect is closed.
Step	Placement & Fixation of Mesh over Abdominal Wall	Mesh is placed over the abdominal wall to reinforce weakened abdominal wall and fixed with a tacking device, glue or suture to prevent folding or migration.	First grasping tool interaction with mesh with intent to place and fix the mesh over the abdominal wall.	Last grasping tool interaction with mesh, tack or glue or last cutting tool interaction with mesh fixation suture after mesh has been fixed over the abdominal wall.
Task	Placement of Mesh over Abdominal Wall	Mesh is placed over the abdominal wall to reinforce weakened abdominal wall.	First grasping tool interaction with mesh with intent to place the mesh over the abdominal wall.	Last grasping tool interaction with mesh after mesh has been placed over the abdominal wall.
Task	Fixation of Mesh over Abdominal Wall	Mesh is fixed over the abdominal wall to prevent folding or migration.	First visualization of tacking or glueing device or needle interaction with mesh with intent to fix the mesh over the abdominal wall	Last visualization of tacking or glueing device or last cutting tool interaction with mesh fixation suture after the mesh has been fixed over the abdominal wall.
Step	Closure of Peritoneum	Peritoneum is reapproximated to close the peritoneum.	First needle interaction with peritoneum with intent to close the peritoneum.	Last cutting tool interaction with peritoneal closure suture after the peritoneum has been closed.

eTable 8. Temporal annotation card specific to robotic-assisted ventral hernia repair. For each defined surgical segment, provided as its own row, the table includes the ontological granularity level, the segment name, its surgical objective, and the start and stop parameters for each. Shaded rows are the recommended annotation segments that balance clinical relevance and effort. *Indicates a truncated “Dissection” surgical objective, which is fully provided in Figure 2 of the manuscript.

Gynecologic Surgery, Robotic-assisted Hysterectomy , eTable 9

Ontology	Name	Surgical Objective	Start Parameter	Stop Parameter
Phase	Exposure	Exploration and preparation of the visual field with intent to expose target anatomy prior to procedure type-specific surgical activities, including sweeping or general dissection of non-target anatomy and/or removal of previous surgical material	Mirrors start parameter of first nested chronological segment annotated beneath Exposure phase.	Mirrors stop parameter of last nested chronological segment annotated beneath Exposure phase.
Step	Tool Installation	Installation of robotic instruments through ports into the body cavity to prepare for surgical activity.	First visualization of first tool as it is installed into the body cavity.	Last tool movement immediately after last tool is installed into body cavity.
Step	Initial Exposure	Actions performed to expose and assess target anatomy in preparation for procedure-specific activities.	First tool interaction with solid organ, bowel, omentum, adhesions, or previous surgical material with intent to expose and assess target anatomy in preparation for dissection.	Last tool interaction with solid organ, bowel, omentum, adhesions, or previous surgical material such that target anatomy is exposed, assessed, and surgical field is ready for dissection.
Task	Exploration of Pelvis	Exploration of pelvic region to assess pathology.	First endoscope focus on pelvic anatomy with intent to explore anatomy and assess pathology.	End of exploratory endoscope movements across pelvis after pathology is assessed.
Task	Bowel / Omentum Sweep	Sweeping of bowel or omentum to adequately expose target anatomy.	First tool interaction with bowel or omentum with intent to expose target anatomy.	Last tool interaction with bowel or omentum to expose target anatomy.
Task	Lysis of Adhesions	Removal of adhesions to prepare visual field and expose target anatomy.	First dissecting tool interaction with adhesions with intent to expose target anatomy.	Last dissecting tool interaction with adhesions to expose target anatomy.
Task	Mobilization of Colon or Rectum to Expose the Uterus	Mobilization of rectum, sigmoid or descending colon as needed to adequately expose the uterus.	First dissecting tool interaction with colon or rectum with intent to mobilize rectum and expose the uterus.	Last dissecting tool interaction with colon or rectum after the uterus has been exposed.
Phase	Dissection	Surgical activities to gain access to and/or prepare target anatomy for subsequent transection, reconstruction, and/or extraction.	Mirrors start parameter of first nested chronological segment annotated beneath Dissection phase.	Mirrors stop parameter of last nested chronological segment annotated beneath Dissection phase.
Step	Dissection of Vesicouterine Pouch to Mobilize Bladder	Dissection of the vesicouterine pouch to mobilize bladder.	First dissecting tool interaction with vesicouterine pouch with intent to mobilize bladder.	Last dissecting tool interaction with vesicouterine pouch after the bladder has been mobilized.
Phase	Transection	Permanent division of target anatomy into two distinct structures or distinct functional compartments for access to subsequent target anatomy or in preparation for reconstruction or extraction.	Mirrors start parameter of first nested chronological segment annotated beneath Transection phase.	Mirrors stop parameter of last nested chronological segment annotated beneath Transection phase.
Step	D&T of Adnexal Structures	Selective dissection and transection of ovaries, fallopian tubes, broad and/or parametrial ligaments.	First dissecting tool interaction with adnexal structures with intent to dissect and transect the relevant adnexal structures.	Last dissecting tool interaction with adnexal structures after the relevant adnexa have been dissected and transected.
Step	D&T of Uterine Vessels* *	Dissection and transection of the uterine vessels to gain vascular control.	First dissecting tool interaction with vessels or perivascular tissue with intent to dissect and transect the uterine vessels.	Last dissecting tool interaction with vessels after the uterine vessels have been dissected and transected.
Step	Transection of Cervicovaginal Junction to Free the Uterus	Transection of the cervicovaginal junction with colpotomy to free the uterus from its inferior attachments.	First colpotomy tool interaction with cervicovaginal junction with intent to transect the cervicovaginal junction and free	Last colpotomy tool interaction with cervicovaginal junction after the cervicovaginal junction has been

			the uterus from its inferior attachments.	transected and the uterus has been freed from its inferior attachments.
Phase	Extraction	Collection and removal of isolated specimens from the body.	Mirrors start parameter of first nested chronological segment annotated beneath Extraction phase.	Mirrors stop parameter of last nested chronological segment annotated beneath Extraction phase.
Step	Extraction of the Uterus & Adnexal Structures	Extraction of resected uterus and adnexal structures to remove specimen from the body.	First grasping tool interaction with uterus or adnexal structures with intent to place in specimen bag.	Last visualization of uterus or last adnexal structure or specimen bag containing uterus and adnexal structures.
Phase	Reconstruction	Realignment of formerly exposed, dissected, or transected anatomy or buttressing of weakened structures to restore structural and/or physiological function.	Mirrors start parameter of first nested chronological segment annotated beneath Reconstruction phase.	Mirrors stop parameter of last nested chronological segment annotated beneath Reconstruction phase.
Step	Closure of the Vaginal Cuff	Suture closure of the vaginal cuff.	First needle interaction with vaginal cuff with intent to close the vaginal cuff.	Last cutting tool interaction with vaginal cuff closure suture after the vaginal cuff has been closed.
Step	Dissection of Lymph Nodes	Dissection of lymph nodes for staging purposes.	First dissecting tool interaction with lymph node or perinodal tissue with intent to dissect the lymph node(s).	Last dissecting tool interaction with lymph node or perinodal tissue after the lymph node(s) have been dissected.
Task	Dissection of Pelvic Lymph Nodes	Dissection of pelvic lymph nodes for staging purposes.	First dissecting tool interaction with pelvic lymph node or perinodal tissue with intent to dissect the lymph node(s).	Last dissecting tool interaction with pelvic lymph node or perinodal tissue after the pelvic lymph node(s) have been dissected.
Task	Dissection of Para-aortic Lymph Nodes	Dissection of para-aortic lymph nodes for staging purposes.	First dissecting tool interaction with para-aortic lymph node or perinodal tissue with intent to dissect the lymph node(s).	Last dissecting tool interaction with para-aortic lymph node or perinodal tissue after the para-aortic lymph node(s) have been dissected.
Step	Extraction of Lymph Nodes	Extraction of resected lymph node(s) to remove specimens from the body.	First grasping tool interaction with lymph node(s) with intent to remove from the body or place in specimen bag.	Last visualization of lymph node or specimen bag containing lymph node(s).

eTable 9. Temporal annotation card specific to robotic-assisted hysterectomy. For each defined surgical segment, provided as its own row, the table includes the ontological granularity level, the segment name, its surgical objective, and the start and stop parameters for each. Shaded rows are the recommended annotation segments that balance clinical relevance and effort. *Indicates a truncated “Dissection” surgical objective, which is fully provided in Figure 2 of the manuscript. ** Indicates right and left options. Abbreviations: D&T, dissection and transection.

Upper Gastrointestinal Surgery, Robotic-assisted Hiatal Hernia Repair & Fundoplication, eTable 10

Ontology	Name	Surgical Objective	Start Parameter	Stop Parameter
Phase	Exposure	Exploration and preparation of the visual field with intent to expose target anatomy prior to procedure type-specific surgical activities, including sweeping or general dissection of non-target anatomy and/or removal of previous surgical material.	Mirrors start parameter of first nested chronological segment annotated beneath Exposure phase.	Mirrors stop parameter of last nested chronological segment annotated beneath Exposure phase.
Step	Tool Installation	Installation of robotic instruments through ports into the body cavity to prepare for surgical activity.	First visualization of first tool as it is installed into the body cavity.	Last tool movement immediately after last tool is installed into body cavity.
Step	Initial Exposure	Actions performed to expose and assess target anatomy in preparation for procedure-specific activities.	First tool interaction with solid organ, bowel, omentum, adhesions, or previous surgical material with intent to expose and assess target anatomy in preparation for dissection.	Last tool interaction with solid organ, bowel, omentum, adhesions, or previous surgical material such that target anatomy is exposed, assessed, and surgical field is ready for dissection.
Task	Exploration of Abdomen	Exploration of abdomen to assess pathology.	First endoscope focus on abdominal anatomy with intent to explore anatomy and assess pathology.	End of exploratory endoscope movements across abdomen after pathology is assessed.
Task	Bowel / Omentum Sweep	Sweeping of bowel or omentum to adequately expose target anatomy.	First tool interaction with bowel or omentum with intent to expose target anatomy.	Last tool interaction with bowel or omentum to expose target anatomy.
Task	Lysis of Adhesions	Removal of adhesions to prepare visual field and expose target anatomy.	First dissecting tool interaction with adhesions with intent to expose target anatomy.	Last dissecting tool interaction with adhesions to expose target anatomy.
Task	Retraction of Liver	Retraction of liver to facilitate adequate exposure of target anatomy.	First retracting tool interaction with liver with intent to expose target anatomy.	Last retracting tool interaction with liver after liver has been retracted to expose target anatomy.
Step	Reduction of Major Viscera into Abdominal Cavity	Major viscera herniated into the chest [stomach, large bowel, additional organ(s)] are reduced back into the abdominal cavity.	First grasping tool interaction with herniated viscera with intent to reduce herniated viscera.	Last grasping tool interaction with herniated viscera after herniated viscera have been reduced.
Task	Reduction of Stomach	Herniated stomach is reduced back into the abdomen.	First grasping tool interaction with herniated stomach with intent to reduce herniated stomach.	Last grasping tool interaction with herniated stomach after herniated stomach has been reduced.
Task	Reduction of Large Bowel	Herniated large bowel is reduced back into the abdomen.	First grasping tool interaction with herniated large bowel with intent to reduce herniated large bowel.	Last grasping tool interaction with herniated large bowel after herniated large bowel has been reduced.
Task	Reduction of Additional Organs	Additional herniated organ(s) is/are reduced back into the abdomen.	First grasping tool interaction with herniated organ with intent to reduce additional herniated organ(s).	Last grasping tool interaction with herniated organ after herniated additional organ(s) have been reduced.
Phase	Dissection	Surgical activities to gain access to and/or prepare target anatomy for subsequent transection, reconstruction, and/or extraction.*	Mirrors start parameter of first nested chronological segment annotated beneath Dissection phase.	Mirrors stop parameter of last nested chronological segment annotated beneath Dissection phase.

Step	Mobilization of Hiatal Hernia	Dissection of the diaphragmatic crura, posterior adhesions and the mediastinum from the hiatal hernia to mobilize the hiatal hernia.	First dissecting tool interaction with diaphragmatic crura, posterior adhesions, mediastinum or hiatal hernia with intent to mobilize the hiatal hernia.	Last dissecting tool interaction with diaphragmatic crura, posterior adhesions, mediastinum or hiatal hernia after the hiatal hernia has been mobilized.
Task	Dissection of Gastrohepatic Ligament & Phrenoesophageal Membrane to Access Diaphragmatic Crus	Dissection of gastrohepatic ligament and phrenoesophageal membrane to access the diaphragmatic crus.	First dissecting tool interaction with gastrohepatic ligament or phrenoesophageal membrane with intent to access the diaphragmatic crus.	Last dissecting tool interaction with gastrohepatic ligament or phrenoesophageal membrane after the diaphragmatic crus has been accessed.
Task	Dissection of Right Diaphragmatic Crus to Mobilize Hiatal Hernia	Dissection of right diaphragmatic crus to mobilize the hiatal hernia from the right diaphragmatic crus.	First dissecting tool interaction with right diaphragmatic crus or hiatal hernia with intent to mobilize the hiatal hernia from the right diaphragmatic crus.	Last dissecting tool interaction with right diaphragmatic crus or hiatal hernia after the hiatal hernia has been mobilized from the right diaphragmatic crus.
Task	Dissection of Left Diaphragmatic Crus to Mobilize Hiatal Hernia	Dissection of left diaphragmatic crus to mobilize the hiatal hernia from the left diaphragmatic crus.	First dissecting tool interaction with left diaphragmatic crus or hiatal hernia with intent to mobilize the hiatal hernia from the left diaphragmatic crus.	Last dissecting tool interaction with left diaphragmatic crus or hiatal hernia after the hiatal hernia has been mobilized from the left diaphragmatic crus.
Task	Dissection of Posterior Adhesions to Mobilize Hiatal Hernia	Dissection of adhesions posterior to the esophagus and stomach to mobilize the hiatal hernia from the adhesionsposterior to the esophagus and stomach.	First dissecting tool interaction with adhesions posterior to the stomach or esophagus or hiatal hernia with intent to mobilize the hiatal hernia from adhesions posterior to the esophagus and stomach.	Last dissecting tool interaction with adhesions posterior to the stomach or esophagus or hiatal hernia after the hiatal hernia has been mobilized from adhesions posterior to the esophagus and stomach.
Task	Dissection of Mediastinum to Mobilize Hiatal Hernia	Dissection of mediastinum to mobilize the hiatal hernia.	First dissecting tool interaction with mediastinum with intent to mobilize the hiatal hernia from the mediastinum.	Last dissecting tool interaction with mediastinum after the hiatal hernia has been mobilized from the mediastinum.
Subtask	Limited Dissection of Mediastinum to Mobilize Hiatal Hernia	Limited dissection of less than 5cm into the mediastinum to mobilize the hiatal hernia.	First dissecting tool interaction with mediastinum with intent to mobilize the hiatal hernia from the mediastinum.	Last dissecting tool interaction with mediastinum after the hiatal hernia has been mobilized from the mediastinum.
Subtask	Extended Dissection of Mediastinum to Mobilize Hiatal Hernia	Extended dissection of over 5cm into the mediastinum to mobilize the hiatal hernia.	First dissecting tool interaction with mediastinum with intent to mobilize the hiatal hernia from the mediastinum.	Last dissecting tool interaction with mediastinum after an extended dissection has been performed to mobilize the hiatal hernia from the mediastinum.
Step	Reduction of Hiatal Hernia	Reduction of the hiatal hernia.	First dissecting tool interaction with hiatal hernia with intent to reduce the hiatal hernia.	Last dissecting tool interaction with hiatal hernia after the hiatal hernia has been reduced.
Phase	Reconstruction	Realignment of formerly exposed, dissected, or transected anatomy or buttressing of weakened structures to restore structural and/or physiological function.	Mirrors start parameter of first nested chronological segment annotated beneath Reconstruction phase.	Mirrors stop parameter of last nested chronological segment annotated beneath Reconstruction phase.
Step	Creation of Relaxing Incision	Placement of relaxing incision when adequate closure cannot be achieved with posterior suturing due to the size of the defect.	First dissecting tool interaction with diaphragmatic crura with intent to place a relaxing incision.	Last dissecting tool interaction with diaphragmatic crura after the relaxing incision has been placed.
Step	Construction of Cruraplasty for Closure of Esophageal Hiatal Defect	Construction of cruraplasty for posterior reapproximation and suturing of the left and right crura to close the hiatal defect.	First needle interaction with left or right crura to construct a cruraplasty with intent to close the hiatal defect.	Last cutting tool interaction with cruraplasty sutures after the hiatal defect is closed.
Step	Placement & Fixation of Mesh over Cruraplasty	Mesh is placed over the cruraplasty to reinforce the reconstructed	First grasping tool interaction with mesh with intent to place	Last grasping tool interaction with mesh, tack or glue or last cutting tool

		diaphragmatic hiatus and fixed with a tacking device, glue or suture to prevent folding or migration.	and fix the mesh over the cruraplasty.	interaction with mesh fixation suture after mesh has been fixed over the cruraplasty.
Task	Placement of Mesh over Cruraplasty	Mesh is placed over the cruraplasty to reinforce the reconstructed diaphragmatic hiatus.	First grasping tool interaction with mesh with intent to place the mesh over the cruraplasty.	Last grasping tool interaction with mesh after mesh has been placed over the cruraplasy.
Task	Fixation of Mesh over Cruraplasty	Mesh is fixed over the cruraplasty to prevent folding or migration.	First visualization of tacking or glueing device or first needle interaction with mesh with intent to fix the mesh over the cruraplasty.	Last visualization of tacking or glueing device or last cutting tool interaction with mesh fixation suture after mesh has been fixed over the cruraplasty.
Step	Takedown of Previous Fundoplication	Revision procedures where previous fundoplication is taken down.	First dissecting tool interaction with adhesions, suture material, staples or previous fundoplication with intent to takedown the previous fundoplication.	Last dissecting tool interaction with adhesions, suture material, staples or gastric fundus after the previous fundoplication has been taken down.
Task	Lysis of Adhesions on Previous Fundoplication	Lysis of adhesions to facilitate takedown of previous fundoplication.	First dissecting tool interaction with adhesions on previous fundoplication with intent to takedown the adhesions.	Last dissecting tool interaction with adhesions after the adhesions on the previous fundoplication have been taken down.
Task	Removal of Suture Material or Staples on Previous Fundoplication	Removal of suture material or staples on previous fundoplication to facilitate take down of the fundoplication.	First dissecting tool interaction with suture materials or staples on previous fundoplication with intent to remove the suture materials or staples.	Last dissecting tool interaction with suture materials or staples on previous fundoplication after the suture material or staples on previous fundoplication have been removed.
Task	Unfolding of Previous Fundoplication Wrap	Unfolding of previous fundoplication wrap.	First dissecting tool interaction with previous fundoplication wrap with intent to unfold the previous fundoplication wrap.	Last dissecting tool interaction with previous fundoplication wrap after the previous fundoplication wrap has been unfolded.
Step	Mobilization of Gastric Fundus	Dissection of gastrosplenic ligament, short gastric vessels and posterior adhesions to mobilize the gastric fundus.	First dissecting tool interaction with gastrosplenic ligament or posterior adhesions with intent to mobilize the gastric fundus.	Last dissecting tool interaction with gastrosplenic ligament or posterior adhesions after the gastric fundus has been mobilized.
Task	Dissection of Gastrosplenic Ligament & Short Gastric Vessels	Dissection of gastrosplenic ligament which contains the short gastric vessels to free it from the greater curvature.	irst dissecting tool interaction with gastrosplenic ligament with intent to dissect the gastrosplenic ligament and free it from the greater curvature.	Last dissecting tool interaction with gastrosplenic ligament after the gastrosplenic ligament has been dissected and freed from the greater curvature.
Task	Dissection of Adhesions Posterior to Gastric Fundus	Dissection of adhesions posterior to the gastric fundus to mobilize the gastric fundus.	First dissecting tool interaction with adhesions posterior to the gastric fundus with intent to mobilize the gastric fundus.	Last dissecting tool interaction with adhesions posterior to the gastric fundus after the gastric fundus has been mobilized.
Step	Dissection of Remnant Tissue in Preparation for Fundoplication	Dissection of remnant tissue including fibrofatty tissue, adhesions and phrenicoesophageal membrane in preparation for construction of fundoplication.	First dissecting tool interaction with remnant tissue with intent to dissect remnant tissue in preparation for construction of fundoplication.	Last dissecting tool interaction with remnant tissue after the remnant tissue has been dissected in preparation for construction of fundoplication.
Step	Placement of Marking Stitch on Posterior Wall of Stomach	Placement of a loose stitch on the posterior gastric wall to mark the proposed site for the first stitch of the fundoplication.	First needle interaction with posterior wall of the stomach with intent to place a marking stitch.	Last cutting tool interaction with marking stitch sutures after marking stitch has been placed on the posterior wall of the stomach.
Step	Construction of Fundoplication Wrap	Construction of fundoplication wrap using the gastric fundus.	First grasping tool interaction with gastric fundus with intent to construct a fundoplication wrap.	Last grasping tool interaction with gastric fundus after fundoplication wrap has been constructed.
Task	Construction of 360 Degree Posterior Fundoplication Wrap	Construction of 360-degree poster fundoplication wrap using the gastric fundus.	First grasping tool interaction with gastric fundus with intent to construct a 360-degree fundoplication wrap.	Last grasping tool interaction with gastric fundus after 360-degree posterior fundoplication wrap has been constructed.

Task	Construction of 270 Degree Posterior Fundoplication Wrap	Construction of 270-degree posterior fundoplication wrap using the gastric fundus.	First grasping tool interaction with gastric fundus with intent to construct a 270-degree posterior fundoplication wrap.	Last grasping tool interaction with gastric fundus after 270-degree posterior fundoplication wrap has been constructed.
Task	Construction of 180 – 200 Degree Anterior Fundoplication Wrap	Construction of 180-200- degree anterior fundoplication wrap using the gastric fundus.	First grasping tool interaction with gastric fundus with intent to construct a 180 - 200-degree anterior fundoplication wrap.	Last grasping tool interaction with gastric fundus after 180-200-degree anterior fundoplication wrap has been constructed.
Step	Placement of Securing & Anchoring Sutures to Fundoplication	Securing sutures are placed between the fundoplication wrap and the esophagus to secure the fundoplication wrap and anchoring sutures are placed between the fundoplication and the diaphragmatic crura to anchor the fundoplication.	First needle interaction with fundoplication wrap with intent to secure the fundoplication wrap.	Last cutting tool interaction with fundoplication securing or anchoring sutures after fundoplication is secured or anchored.
Task	Placement of Securing Sutures to Fundoplication Wrap	Securing sutures are placed between the fundoplication wrap and the esophagus to secure the fundoplication.	First needle interaction with fundoplication wrap with intent to secure the fundoplication.	Last cutting tool interaction with fundoplication securing sutures after fundoplication wrap is secured.
Task	Placement of Anchoring Sutures between Fundoplication and Diaphragm	Anchoring sutures are placed between the fundoplication and the diaphragmatic crura to anchor the fundoplication.	First needle interaction with fundoplication with intent to anchor the fundoplication.	Last cutting tool interaction with fundoplication anchoring sutures after fundoplication is anchored.

eTable 10. Temporal annotation card specific to robotic-assisted hiatal hernia repair and fundoplication. For each defined surgical segment, provided as its own row, the table includes the ontological granularity level, the segment name, its surgical objective, and the start and stop parameters for each. Shaded rows are the recommended annotation segments that balance clinical relevance and effort. *Indicates a truncated “Dissection” surgical objective, which is fully provided in Figure 2 of the manuscript.

Urologic Surgery, Robotic-assisted Radical Prostatectomy, eTable 11

Ontology	Name	Surgical Objective Start Parameter	Start Parameter	Stop Parameter
Phase	Exposure	Exploration and preparation of the visual field with intent to expose target anatomy prior to procedure type-specific surgical activities, including sweeping or general dissection of non-target anatomy and/or removal of previous surgical material	Mirrors start parameter of first nested chronological segment annotated beneath Exposure phase.	Mirrors stop parameter of last nested chronological segment annotated beneath Exposure phase.
Step	Tool Installation	Installation of robotic instruments through ports into the body cavity to prepare for surgical activity.	First visualization of first tool as it is installed into the body cavity.	Last tool movement immediately after last tool is installed into body cavity.
Step	Initial Exposure	Actions performed to expose and assess target anatomy in preparation for procedure-specific activities.	First tool interaction with solid organ, bowel, omentum, adhesions, or previous surgical material with intent to expose and assess target anatomy in preparation for dissection.	Last tool interaction with solid organ, bowel, omentum, adhesions, or previous surgical material such that target anatomy is exposed, assessed, and surgical field is ready for dissection.
Task	Exploration of Pelvis	Exploration of pelvic region to assess pathology.	First endoscope focus on pelvic anatomy with intent to explore anatomy and assess pathology.	End of exploratory endoscope movements across pelvis after pathology is assessed.
Task	Bowel / Omentum Sweep	Sweeping of bowel or omentum with intent to expose target anatomy.	First tool interaction with bowel or omentum with intent to expose target anatomy.	Last tool interaction with bowel or omentum to expose target anatomy.
Task	Lysis of Adhesions	Removal of adhesions to prepare visual field and expose target anatomy.	First dissecting tool interaction with adhesions with intent to expose target anatomy.	Last dissecting tool interaction with adhesions to expose target anatomy.
Task	Mobilization of Rectum or Colon to Expose the Retropubic Space	Creation of a plane between the mesentery and retroperitoneal structures to mobilize the rectum, descending or sigmoid colon and expose the retropubic space.	First dissecting tool interaction with mesentery or lateral attachments with intent to mobilize rectum, sigmoid or descending colon and expose the retropubic space.	Last dissecting tool interaction with mesentery or lateral attachments after rectum, sigmoid or descending colon have been mobilized and the retropubic space has been exposed.
Phase	Dissection	Surgical activities to gain access to and/or prepare target anatomy for subsequent transection, reconstruction, and/or extraction.*	Mirrors start parameter of first nested chronological segment annotated beneath Dissection phase.	Mirrors stop parameter of last nested chronological segment annotated beneath Dissection phase.
Step	Dissection of Preperitoneal Space to Mobilize Bladder & Defat Prostate	Dissection of preperitoneal space beginning at umbilical ligaments to mobilize bladder and defat the prostate.	First dissecting tool interaction with umbilical ligament with intent to drop the bladder and defat the prostate.	Last dissecting tool interaction with periprostatic fat after the bladder has been dropped and prostate has been defatted.
Step	Dissection of Endopelvic Fascia to Access the Prostate	Dissection of the endopelvic fascia to access the prostate.	First dissecting tool interaction with endopelvic fascia with intent to dissect the endopelvic fascia and access the prostate.	Last dissecting tool interaction with prostatic or endopelvic fascia after the fascia has been dissected and prostate accessed.
Phase	Transection	Permanent division of target anatomy into two distinct structures or distinct functional compartments for access to subsequent target anatomy or in preparation for reconstruction or extraction.	Mirrors start parameter of first nested chronological segment annotated beneath Transection phase.	Mirrors stop parameter of last nested chronological segment annotated beneath Transection phase.

Step	D&T of Bladder Neck	The bladder neck is dissected and transected.	First dissecting or transecting tool interaction with bladder neck with intent to dissect the bladder neck.	Last dissecting or transecting tool interaction with bladder neck after the bladder neck has been transected.
Task	D&T of Anterior Bladder Neck	The anterior bladder neck is dissected and transected.	First dissecting tool interaction with anterior bladder neck with intent to dissect and transect the anterior bladder neck.	Last dissecting tool interaction with anterior bladder neck after the anterior bladder neck has been dissected and transected.
Task	D&T of Posterior Bladder Neck	The posterior bladder neck is dissected and transected to free prostate specimen superiorly.	First dissecting tool interaction with posterior bladder neck with intent to dissect and transect the posterior bladder neck.	Last transecting tool interaction with posterior bladder neck after the posterior bladder neck has been dissected and transected.
Step	D&T of Prostatic Pedicle**	The prostatic pedicle is dissected and transected.	First dissecting tool interaction with prostatic pedicle with intent to dissect and transect the prostatic pedicle.	Last dissecting tool interaction with prostatic pedicle after the prostatic pedicle has been dissected and transected.
Step	D&T of Seminal Vesicles & Vas Deferens***	The seminal vesicles and Vas Deferens are dissected and transected.	First dissecting tool interaction with seminal vesicles and vas deferens with intent to dissect and transect the seminal vesicles and vas deferens.	Last dissecting tool interaction with seminal vesicles and vas deferens after the seminal vesicles and vas deferens have been dissected and transected.
Step	Dissection of Denonvillier's Fascia***	Dissection of Denonvillier's Fascia to separate the prostate from the rectum.	First dissecting tool interaction with Denonvillier's fascia with intent to separate the prostate from the rectum.	Last dissecting tool interaction with Denonvillier's fascia after the prostate has been separated from the rectum.
Step	Apical Dissection & Transection of Urethra	Dissection of the apex of the prostate to facilitate transection of the urethra and free the prostate inferiorly.	First dissecting tool interaction with prostate with intent to orientate it for apical dissection and urethral transection.	Last dissecting tool interaction with prostate after apical dissection is completed and urethra is transected.
Phase	Reconstruction	Realignment of formerly exposed, dissected, or transected anatomy or buttressing of weakened structures to restore structural and/or physiological function.	Mirrors start parameter of first nested chronological segment annotated beneath Reconstruction phase.	Mirrors stop parameter of last nested chronological segment annotated beneath Reconstruction phase.
Step	Ligation of Dorsal Venous Complex	Suture ligation of dorsal venous complex to minimize risk of bleeding.	First needle interaction with perivascular tissue of the dorsal venous complex with intent to ligate the dorsal venous complex.	Last cutting tool interaction with dorsal venous complex stitch suture after the dorsal venous complex stitch has been placed and dorsal venous complex has been ligated.
Step	Reinforcement of Rhabdosphincter	Rocco stitch to reinforce the rhabdosphincter.	First needle interaction with bladder or urethra with intent to reinforce the rhabdosphincter with the Rocco stitch.	Last cutting tool interaction with Rocco stitch suture after the Rocco stitch has been completed and the rhabdosphincter has been reinforced.
Step	Creation of Vesicourethral Anastomosis	Creation of a vesicourethral anastomosis to reestablish continuity of the urinary tract between bladder and urethra.	First needle interaction with bladder or urethra with intent to anastomose the urethra and the bladder.	Last cutting tool interaction with vesicourethral anastomotic suture after the anastomosis has been created.
Step	Dissection of Lymph Nodes	Dissection of lymph nodes for staging purposes.	First dissecting tool interaction with lymph node or perinodal tissue with intent to dissect the lymph node.	Last dissecting tool interaction with lymph node or perinodal tissue after the lymph node(s) have been dissected.
Task	Limited Dissection of Lymph Nodes	Limited dissection of lymph nodes for staging purposes.	First dissecting tool interaction with lymph node or perinodal tissue with intent to perform a limited dissection of lymph node(s).	Last dissecting tool interaction with lymph node or perinodal tissue after the lymph node(s) have been dissected.
Task	Extended Dissection of Lymph Nodes	Extended dissection of lymph nodes for staging purposes.	First dissecting tool interaction with lymph node or perinodal tissue with intent to perform an extended dissection of lymph node(s).	Last dissecting tool interaction with lymph node or perinodal tissue after the lymph node(s) have been dissected.
Phase	Extraction	Collection and removal of isolated specimens from the body.	Mirrors start parameter of first nested chronological segment annotated beneath Extraction phase.	Mirrors stop parameter of last nested chronological segment annotated beneath Extraction phase.

Step	Extraction of Prostate	Extraction of resected prostate to remove specimen from the body.	First grasping tool interaction with prostate specimen with intent to remove from the body or place in specimen bag.	Last visualization of prostate specimen or specimen bag containing prostate.
Step	Extraction of Lymph Nodes	Extraction of resected lymph node(s) to remove specimen from the body.	First grasping tool interaction with lymph node(s) with intent to remove from the body or place in specimen bag	Last visualization of lymph node or specimen bag containing lymph node(s).

eTable 11. Temporal annotation card specific to robotic-assisted radical prostatectomy. For each defined surgical segment, provided as its own row, the table includes the ontological granularity level, the segment name, its surgical objective, and the start and stop parameters for each. Shaded rows are the recommended annotation segments that balance clinical relevance and effort. *Indicates a truncated "Dissection" surgical objective, which is fully provided in Figure 2 of the manuscript. ** indicates right and left options. *** indicates Anterior approach / Posterior approach. Abbreviations: D&T, dissection and transection.
