# Supplementary material for: A Standardized Temporal Segmentation Framework and Annotation Resource Library in Robotic Surgery
Source: Mayo Clin Proc Digit Health. 2025 Aug 22;3(4):100257. doi: 10.1016/j.mcpdig.2025.100257 (PMC12492233; doi:10.1016/j.mcpdig.2025.100257)
Supplement: Supplementary Figures 2 [file mmc5.pdf]

## Gastric Bypass

| | | | | | | | | | | | | | | | | | | | | | | | | | | | | |
|---|---|---|---|---|---|---|---|---|---|---|---|---|---|---|---|---|---|---|---|---|---|---|---|---|---|---|---|---|
| **Phases** | Exposure | | | | | Dissection | | | Transection | Reconstruction | | Transection | | | | Dissection | | Reconstruction | | | | | | | | | | |
| **Steps** | Tool Installation | Initial Exposure | | | | Dissection of Gastrohepatic Ligament & Posterior Adhesions for Access to Lesser Curvature & Lesser Sac | Tunnelling Dissection of Posterior Adhesions & Gastrophrenic Ligament | | Stapler Transection of Stomach | Reinforcement of Gastric Staple Line | | Transection of Omentum | Measurement of Small Bowel | | Transection of Small Bowel | Dissection of Small Bowel Mesentery | Tunnelling Dissection of Transerve Mesocolon | Creation of JJ** or GJ** | | | | Reinforcement of Anastomosis | | Closure of Mesenteric Defect(s) | | | Placement & Fixation of Omental Flap over GJ | Repair of Inadvertent Bowel Enterotomy |
| **Tasks** | | Exploration of Abdomen | Bowel / Omentum Sweep | Lysis of Adhesions | Retraction of Liver | | Tunnelling Dissection of Posterior Adhesions | Tunnelling Dissection of Gastrophrenic Ligament | | Reinforcement of Gastric Pouch Staple Line | Reinforcement of Gastric Remnant Staple Line | | Measurement of PB Limb | Measurement of Alimentary Limb | | | | Creation of JJ** | Creation of GJ** | Closure of Common JJ Enterotomy | Closure of Common GJ Enterotomy | Reinforcement of JJ | Reinforcement of GJ | Closure of JJ Mesenteric Defect | Closure of Petersen's Space | Closure of Transverse Mesocolic Defect | | |

eFigure 2. Temporal annotation card specific to robotic-assisted gastric bypass. For each defined surgical segment, provided as its own row, the table includes the ontological granularity level, the segment name, its surgical objective, and the start and stop parameters for each. Shaded rows are the recommended annotation segments that balance clinical relevance and effort. **Indicates hand sewn or stapled. Abbreviations: PB, pancreaticobiliary; GJ, gastrojejunostomy; JJ, jejunojejunostomy.
