# Supplementary material for: A Standardized Temporal Segmentation Framework and Annotation Resource Library in Robotic Surgery
Source: Mayo Clin Proc Digit Health. 2025 Aug 22;3(4):100257. doi: 10.1016/j.mcpdig.2025.100257 (PMC12492233; doi:10.1016/j.mcpdig.2025.100257)
Supplement: Supplementary Figures 3 [file mmc6.pdf]

Sleeve Gastrectomy

**Phases**	Exposure					Dissection				Transection	Reconstruction	Extraction
**Steps**	Tool Installation	Initial Exposure				Dissection of Greater Omentum & Posterior Adhesions to Mobilize Stomach				Stapler Transection of Stomach	Reinforcement of Gastric Staple Line	Extraction of Gastric Specimen
**Tasks**		Exploration of Abdomen	Bowel / Omentum Sweep	Lysis of Adhesions	Retraction of Liver	Dissection of Greater Omentum to Mobilize Greater Curvature			Dissection of Posterior Adhesions			
**Subtasks**						Dissection of Gastrocolic Ligament	Dissection of Gastrosplenic Ligament & Short Gastric Vessels	Dissection of Gastrophrenic Ligament				

eFigure 3. Temporal annotation card specific to robotic-assisted sleeve gastrectomy. For each defined surgical segment, provided as its own row, the table includes the ontological granularity level, the segment name, its surgical objective, and the start and stop parameters for each. Shaded rows are the recommended annotation segments that balance clinical relevance and effort.
