# Supplementary material for: A Standardized Temporal Segmentation Framework and Annotation Resource Library in Robotic Surgery
Source: Mayo Clin Proc Digit Health. 2025 Aug 22;3(4):100257. doi: 10.1016/j.mcpdig.2025.100257 (PMC12492233; doi:10.1016/j.mcpdig.2025.100257)
Supplement: Supplementary Figures 4 [file mmc7.pdf]

Lobectomy

**Phases**	Exposure			Dissection						Transection				Extraction	
**Steps**	Tool Installation	Initial Exposure		Division of Inferior Pulmonary Ligament	Dissection of Lymph Nodes	Dissection of Lung Fissure	Skeletonization of Pulmonary Artery or Branches	Skeletonization of Pulmonary Vein or Tributaries	Skeletonization of Lobar Bronchus	Transection of Pulmonary Artery or Branches	Transection of Pulmonary Vein or Tributaries	Transection of Lobar Bronchus	Wedge Transection	Extraction of Lung Specimen	Extraction of Lymph Nodes
**Tasks**		Exploration of Thorax	Lysis of Adhesions												

eFigure 4. Temporal annotation card specific to robotic-assisted lobectomy. For each defined surgical segment, provided as its own row, the table includes the ontological granularity level, the segment name, its surgical objective, and the start and stop parameters for each. Shaded rows are the recommended annotation segments that balance clinical relevance and effort.
