# Supplementary material for: A Standardized Temporal Segmentation Framework and Annotation Resource Library in Robotic Surgery
Source: Mayo Clin Proc Digit Health. 2025 Aug 22;3(4):100257. doi: 10.1016/j.mcpdig.2025.100257 (PMC12492233; doi:10.1016/j.mcpdig.2025.100257)
Supplement: Supplementary Figures 5 [file mmc8.pdf]

Inguinal Hernia Repair

**Phases**	Exposure					Dissection			Reconstruction			
**Steps**	Tool Installation	Initial Exposure				Incision of Peritoneum	Exploration of Peritoneal Flap & Reduction of Hernia**		Measurement of Hernia Defect**	Placement & Fixation of Mesh over Myopectineal Orifice**		Closure of Peritoneum**
**Tasks**		Exploration of Pelvis	Bowel / Omentum Sweep	Lysis of Adhesions	Excision of Existing Mesh**		Exploration of Peritoneal Flap**	Reduction of Hernia**		Placement of Mesh over Myopectineal Orifice**	Fixation of Mesh over Myopectineal Orifice**	

eFigure 5. Temporal annotation card specific to robotic-assisted inguinal hernia repair. For each defined surgical segment, provided as its own row, the table includes the ontological granularity level, the segment name, its surgical objective, and the start and stop parameters for each. Shaded rows are the recommended annotation segments that balance clinical relevance and effort.
