# Supplementary material for: A Standardized Temporal Segmentation Framework and Annotation Resource Library in Robotic Surgery
Source: Mayo Clin Proc Digit Health. 2025 Aug 22;3(4):100257. doi: 10.1016/j.mcpdig.2025.100257 (PMC12492233; doi:10.1016/j.mcpdig.2025.100257)
Supplement: Supplementary Figures 6 [file mmc9.pdf]

Ventral Hernia Repair

**Phases**	Exposure					Dissection			Reconstruction							
**Steps**	Tool Installation	Initial Exposure				Incision of Peritoneum	Exploration of Peritoneal Flap & Reduction of Hernia		Creation of Fascial Flap			Measurement of Hernia Defect	Closure of Hernia Defect	Placement & Fixation of Mesh over Abdominal Wall		Closure of Peritoneum
**Tasks**		Exploration of Abdomen	Bowel /Omentum Sweep	Lysis of Adhesions	Excision of Existing Mesh		Exploration of Peritoneal Flap	Reduction of Hernia	Creation of Ipsilateral Fascial Flap	Creation of Contralateral Fascial Flap	Creation of Circumferential Fascial Flap			Placement of Mesh over Abdominal Wall	Fixation of Mesh over Abdominal Wall	

eFigure 6. Temporal annotation card specific to robotic-assisted ventral hernia repair. For each defined surgical segment, provided as its own row, the table includes the ontological granularity level, the segment name, its surgical objective, and the start and stop parameters for each. Shaded rows are the recommended annotation segments that balance clinical relevance and effort.
