# Supplementary material for: A Standardized Temporal Segmentation Framework and Annotation Resource Library in Robotic Surgery
Source: Mayo Clin Proc Digit Health. 2025 Aug 22;3(4):100257. doi: 10.1016/j.mcpdig.2025.100257 (PMC12492233; doi:10.1016/j.mcpdig.2025.100257)
Supplement: Supplementary Figures 7 [file mmc10.pdf]

Hysterectomy

**Phases**	Exposure					Dissection	Transection			Extraction	Reconstruction	Dissection		Extraction
**Steps**	Tool Installation	Initial Exposure				Dissection of Vesicouterine Pouch to Mobilize Bladder	D&T of Adnexal Structures	D&T of Uterine Vessels**	Transection of Cervicovaginal Junction to Free the Uterus	Extraction of the Uterus & Adnexal Structures	Closure of the Vaginal Cuff	Dissection of Lymph Nodes		Extraction of Lymph Nodes
**Tasks**		Exploration of Pelvis	Bowel / Omentum Sweep	Lysis of Adhesions	Mobilization of Colon or Rectum to Expose the Uterus							Dissection of Pelvic Lymph Nodes	Dissection of Para-Aortic Lymph Nodes	

eFigure 7. Temporal annotation card specific to robotic-assisted hysterectomy. For each defined surgical segment, provided as its own row, the table includes the ontological granularity level, the segment name, its surgical objective, and the start and stop parameters for each. Shaded rows are the recommended annotation segments that balance clinical relevance and effort. **Indicates right and left options. Abbreviations: D&T, dissection and transection.
