# Supplementary material for: A Standardized Temporal Segmentation Framework and Annotation Resource Library in Robotic Surgery
Source: Mayo Clin Proc Digit Health. 2025 Aug 22;3(4):100257. doi: 10.1016/j.mcpdig.2025.100257 (PMC12492233; doi:10.1016/j.mcpdig.2025.100257)
Supplement: Supplementary Figures 8 [file mmc11.pdf]

Hiatal Hernia Repair & Fundoplication

Phases	Steps	Tasks	Subtasks
Exposure	Tool Installation		
	Initial Exposure	Exploration of Abdomen	
		Bowel / Omentum Sweep	
		Lysis of Adhesions	
		Retraction of Liver	
	Reduction of Major Viscera into Abdominal Cavity	Reduction of Stomach	
		Reduction of Large Bowel	
		Reduction of Additional Organs	
Dissection	Mobilization of Hiatal Hernia	Dissection of Gastrohepatic Ligament & Phrenoesophageal Membrane to Access Diaphragmatic Crus	
		Dissection of Right Diaphragmatic Crus to Mobilize Hiatal Hernia	
		Dissection of Left Diaphragmatic Crus to Mobilize Hiatal Hernia	
		Dissection of Posterior Adhesions to Mobilize Hiatal Hernia	
		Dissection of Mediastinum to Mobilize Hiatal Hernia	Limited Dissection of Mediastinum to Mobilize Hiatal Hernia
			Extended Dissection of Mediastinum to Mobilize Hiatal Hernia
	Reduction of Hiatal Hernia		
Reconstruction	Creation of Relaxing Incision		
	Construction of Cruraplasty for Closure of Esophageal Hiatal Defect		
	Placement & Fixation of Mesh over Cruraplasty	Placement of Mesh over Cruraplasty	
		Fixation of Mesh over Cruraplasty	
Exposure	Takedown of Previous Fundoplication	Lysis of Adhesions on Previous Fundoplication	
		Removal of Suture Material or Staples on Previous Fundoplication	
		Unfolding of Previous Fundoplication Wrap	
Dissection	Mobilization of Gastric Fundus	Dissection of Gastrosplenic Ligament & Short Gastric Vessels	
		Dissection of Adhesions Posterior to Gastric Fundus	
	Dissection of Remnant Tissue in Preparation for Fundoplication		
Reconstruction	Placement of Marking Stitch on Posterior Wall of Stomach		
	Construction of Fundoplication Wrap	Construction of 360 Degree Posterior Fundoplication Wrap	
		Construction of 270 Degree Posterior Fundoplication Wrap	
		Construction of 180-200 Degree Anterior Fundoplication Wrap	
	Placement of Securing & Anchoring Sutures to the Fundoplication	Placement of Securing Sutures to Fundoplication Wrap	
		Placement of Anchoring Sutures between Fundoplication & Diaphragm	

eFigure 8. Temporal annotation card specific to robotic-assisted hiatal hernia repair and fundoplication. For each defined surgical segment, provided as its own row, the table includes the ontological granularity level, the segment name, its surgical objective, and the start and stop parameters for each. Shaded rows are the recommended annotation segments that balance clinical relevance and effort.
