# Supplementary material for: A Standardized Temporal Segmentation Framework and Annotation Resource Library in Robotic Surgery
Source: Mayo Clin Proc Digit Health. 2025 Aug 22;3(4):100257. doi: 10.1016/j.mcpdig.2025.100257 (PMC12492233; doi:10.1016/j.mcpdig.2025.100257)
Supplement: Supplementary Figures 9 [file mmc12.pdf]

Radical Prostatectomy

**Phases**	Exposure						Dissection		Transection				Dissection	Transection	Reconstruction			Dissection		Extraction	
**Steps**	Tool Installation	Initial Exposure					Dissection of Preperitoneal Space to Mobilize Bladder & Defat Prostate	Dissection of Endopelvic Fascia	D&T of Bladder Neck		D&T of Prostatic Pedicle**	D&T of Seminal Vesicles & Vas Deferens***	Dissection of Denonvilliers Fascia***	Apical Dissection & Transection of Urethra	Ligation of Dorsal Venous Complex	Reinforcement of Rhabdosphincter	Creation of Vesicourethral Anastomosis	Dissection of Lymph Nodes		Extraction of Prostate	Extraction of Lymph Nodes
**Tasks**		Exploration of Pelvis	Bowel / Omentum Sweep	Lysis of Adhesions	Mobilization of Rectum or Colon to Expose the Retropubic Space			D&T of Anterior Bladder Neck	D&T of Posterior Bladder Neck									Limited Dissection of Lymph Nodes	Extended Dissection of Lymph Nodes		

eFigure 9. Temporal annotation card specific to robotic-assisted radical prostatectomy. For each defined surgical segment, provided as its own row, the table includes the ontological granularity level, the segment name, its surgical objective, and the start and stop parameters for each. Shaded rows are the recommended annotation segments that balance clinical relevance and effort. **Indicates right and left options. ***Indicates Anterior approach / Posterior approach. Abbreviations: D&T, dissection and transection.
